# Polo/PLK1 phosphorylation relieves Centrosomin/Cnn autoinhibition to promote centrosome scaffold assembly

**DOI:** 10.1038/s44318-026-00878-x

**Published:** 2026-07-30

**Authors:** Nada Mohamad, Siu-Shing Wong, Anupa Majumdar, Alan Wainman, Ingelise Holland-Kaye, Lars Hubatsch, Zsofia Novak, Andrey Pozniakovsky, Martine Ruer-Gruss, Andreas F M Haensele, Anna Caballe, Steven Johnson, Susan M Lea, Anthony A Hyman, Jordan W Raff

**Affiliations:** 1 Sir William Dunn School of Pathology, University of Oxford, Oxford, UK; 2 The Max Planck Institute of Molecular Cell Biology and Genetics, Dresden, Germany; 3 St. Jude Children’s Research Hospital, 262 Danny Thomas Place, Memphis, TN USA

**Keywords:** Cell Adhesion, Polarity & Cytoskeleton, Cell Cycle, Post-translational Modifications & Proteolysis

## Abstract

Mitotic centrosome maturation requires Polo/PLK1-dependent expansion of the pericentriolar material (PCM). In *Drosophila*, Centrosomin (Cnn) assembles a scaffold around mitotic centrioles through interactions between its PReM and CM2 domains. Here, we show that PReM adopts an autoinhibited helical hairpin conformation that prevents CM2 binding. Polo/PLK1 phosphorylation relieves this autoinhibition, enabling scaffold assembly, whereas phospho-blocking mutations disrupt PReM–CM2 binding in vitro and Cnn scaffold assembly in vivo. Potential functionally analogous domains have been identified in the human and *C. elegans* Cnn homologues CDK5RAP2 and SPD-5. We find that the human protein appears to share a structurally similar mechanism for scaffold assembly, but the worm protein does not. Consistent with this, deletion of these domains alters the dynamics of Cnn condensates in vitro, but has little effect on SPD-5 condensate dynamics. We conclude that Polo/PLK1 promotes mitotic centrosome assembly, at least in part, by relieving autoinhibitory intramolecular interactions.

## Synopsis

Centrosome assembly is triggered by relief of autoinhibition. This work shows that Polo/PLK1 phosphorylation unlocks Centrosomin/Cnn scaffold formation, revealing a conserved mechanism for mitotic centrosome maturation.

- The Cnn PReM domain adopts an autoinhibited helical hairpin that prevents interaction with the CM2 scaffold-forming domain.
- Polo/PLK1 phosphorylation relieves PReM autoinhibition and promotes PReM–CM2 binding.
- Phospho-blocking mutations disrupt Cnn scaffold assembly in vitro and centrosome scaffold formation in vivo.
- A structurally similar autoinhibitory mechanism appears to operate in the human Cnn homologue CDK5RAP2, but not in the worm homologue SPD-5.

## Abstract (web summary)

Drosophila Cnn and its human homologue CDK5RAP2 constitute a structurally similar autoinhibitory mechanism for centrosome assembly, which is not conserved in the worm homologue SPD-5.

## Graphical abstract

**Figure:**
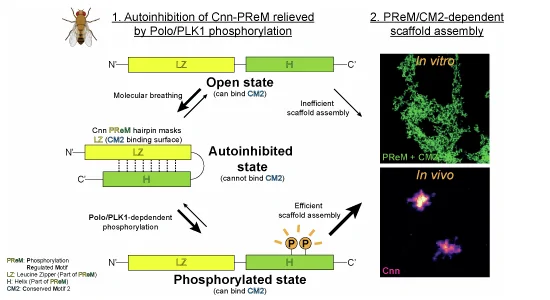

**Figure:**
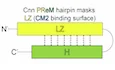

## Introduction

Centrosomes perform many important functions, and their dysfunction has been linked to a plethora of human pathologies, including cancer and microcephaly/primordial dwarfism (Nigg and Raff, 2009; Fernandes-Mariano et al, 2025). Centrosomes are formed when a central pair of centrioles recruits a matrix of pericentriolar material (PCM) around themselves (Conduit et al, 2015; Vasquez-Limeta and Loncarek, 2021; Gomes Pereira et al, 2021; Fernandes-Mariano et al, 2025). During cell division, the amount of PCM at the centrosomes increases dramatically in a process termed centrosome maturation (Palazzo et al, 2000). This allows centrosomes to serve as major sites of microtubule (MT) nucleation during cell division, forming the two poles of the mitotic spindle that segregate the cell’s genetic material in animal cells (Meraldi, 2016; Prosser and Pelletier, 2017).

The mitotic PCM comprises several hundred proteins (da Conceiçao Alves-Cruzeiro et al, 2014; Huang et al, 2015), yet despite this molecular complexity, it can assemble and disassemble rapidly as cells prepare to enter and exit mitosis (Enos et al, 2018; Magescas et al, 2019; Mittasch et al, 2020; Wong et al, 2024). This has prompted much debate about the biophysical nature of the mitotic PCM, which must be mechanically robust enough to withstand forces generated by the MTs it organises, while simultaneously providing an environment in which hundreds of proteins can be concentrated and potentially interact. In particular, it remains unresolved whether liquid-liquid phase separation (LLPS) plays a central part in mitotic centrosome assembly (Rale et al, 2017; Woodruff, 2018; Raff, 2019; Lee et al, 2021; Woodruff, 2021), as has been proposed for several other cellular condensates (Shin and Brangwynne, 2017; Alberti and Hyman, 2021).

Although flies and worms are separated by ~800–1000 million years of evolution (Erwin et al, 2011), a broadly similar set of proteins drives mitotic centrosome assembly in both species. In each case, the centriole and PCM protein Spd-2/SPD-2 (fly/worm nomenclature—CEP192 in humans) (O’Connell et al, 2000; Kemp et al, 2004; Dix and Raff, 2007; Giansanti et al, 2008) recruits the mitotic kinase Polo/PLK-1 (Decker et al, 2011; Cabral et al, 2019; Alvarez Rodrigo et al, 2019; Wong et al, 2022). Polo/PLK-1 then phosphorylates either Cnn (flies) or SPD-5 (worms)—CDK5RAP2 in humans—to stimulate assembly of a macromolecular “scaffold” that recruits most other PCM “client” proteins to mitotic centrosomes (Decker et al, 2011; Woodruff et al, 2015; Cabral et al, 2019; Ohta et al, 2021; Conduit et al, 2014a, 2014b). While Spd-2/SPD-2 and Polo/PLK-1 are clear fly/worm homologues, Cnn and SPD-5 share little obvious sequence homology, although both are large coiled-coil-rich proteins. Thus, it remains unclear whether Cnn and SPD-5 scaffolds assemble and function through similar molecular mechanisms.

The internal molecular interactions that drive Cnn and SPD-5 scaffold assembly are beginning to be elucidated (Feng et al, 2017; Nakajo et al, 2022; Rios et al, 2024). Cnn has two Conserved Motifs (Zhang and Megraw, 2007): CM1, which recruits and regulates the γ-tubulin ring complex (γ-TuRC) (Tovey et al, 2021; Serna et al, 2024; Dendooven et al, 2024; Gao et al, 2025), and CM2, which has been proposed to help target this family of proteins to centrosomes (Wang et al, 2010; Citron et al, 2018). In flies the C-terminal CM2 domain interacts with an internal Phospho-Regulated Multimerization (PReM) domain that contains several potential Polo/PLK1 phosphorylation sites and is centred on a Leucine Zipper (LZ). The crystal structure of this LZ when bound to CM2, forming a tetrameric LZ_dimer_::CM2_dimer_ complex, has been solved (Feng et al, 2017). When the full PReM and CM2 domains are mixed in vitro, they form large micron-scale assemblies and point mutations that perturb the LZ::CM2 tetramer, perturb PReM::CM2 scaffold assembly in vitro and Cnn scaffold assembly in vivo. CDK5RAP2, the human homologue of Cnn, has both CM1 and CM2 domains, but no PReM domain has yet been identified (Yoo et al, 2025). Nevertheless, CDK5RAP2 contains multiple coiled-coil regions, several of which contain potential Polo/PLK1 phosphorylation sites and so could function analogously to the Cnn-PReM domain.

SPD-5 contains an N-terminal region required to recruit γ-tubulin complexes (γ-TuCs) that shares some sequence homology with CM1 (Ohta et al, 2021, 2026), as well as an internal phosphorylated region and a C-terminal region that both promote SPD-5 scaffold assembly. These regions are therefore potentially analogous to the Cnn PReM and CM2 domains, respectively (Ohta et al, 2021; Nakajo et al, 2022; Rios et al, 2024). Thus, despite lacking obvious sequence homology, the internal interactions that promote Cnn and SPD-5 scaffold assembly may be similar, and, for simplicity, we hereafter refer to these SPD-5 regions as PReM and CM2. On the other hand, studies have suggested that the Cnn and SPD-5 scaffolds differ in their physical properties, with the Cnn scaffold being considerably less dynamic than the SPD-5 scaffold (Feng et al, 2017; Woodruff et al, 2017). Here, we investigate how Polo/PLK1 regulates the PReM/CM2 interaction to promote Cnn scaffold assembly, and whether the putative PReM and CM2 domains of CDK5RAP2 and SPD-5 function in an analogous manner.

## Results

### The Cnn PReM domain forms a double helical hairpin

**Figure 1:**
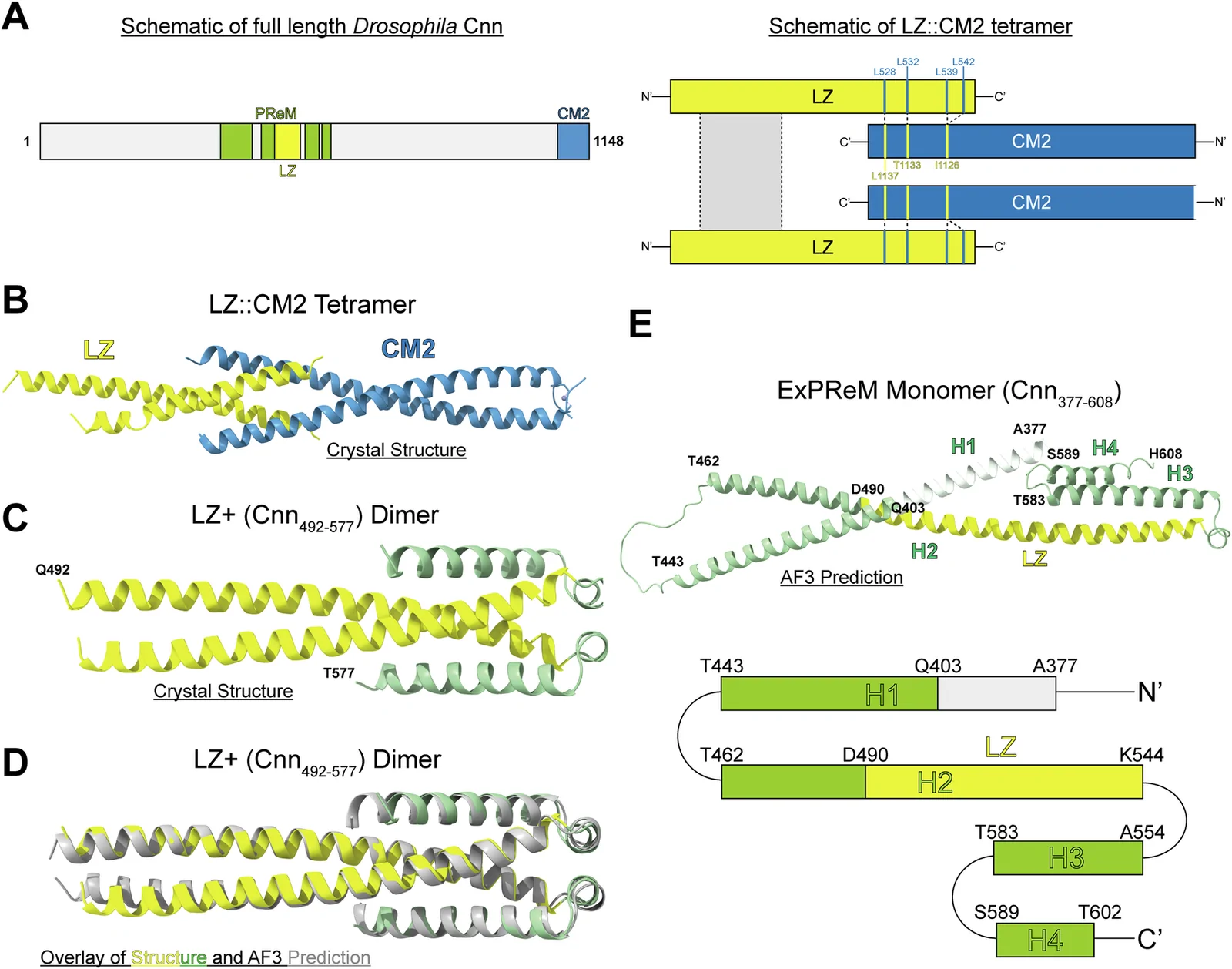
The Drosophila PReM domain adopts a helical hairpin fold. (**A**) Schematic representation of full-length Cnn (left). The PReM domain—containing several predicted helices (green), including the leucine zipper (LZ) (yellow)—and the C-terminal CM2 domain (blue) are highlighted. Schematic representation of the LZ::CM2 tetramer (right), highlighting LZ residues (blue) and CM2 residues (yellow) in the interaction interface; grey shaded region indicates the canonical leucine zipper dimer interface. (**B**) Ribbon diagram of the previously published LZ::CM2 tetramer crystal structure. (**C**) Crystal structure of an extended LZ+ (Cnn_490-579_) dimer, highlighting the original LZ helices (yellow) and the additional helices (green) that fold back on the LZ to form a hairpin. (**D**) An overlay of the LZ+ crystal structure (yellow/green) with the AF3 prediction of the same region (grey). (**E**) AF3 prediction (top) and schematic representation (bottom) of the Extended PReM (ExPReM) monomer comprising 4 helices (H1-H4)—with the H3 and H4 helices folding back on each other and on the C-terminal region of H2 to form a helical hairpin. The original PReM domain (green) and LZ (yellow) are highlighted. AF3 predicts that the N-terminal boundary of the original PReM (Q403) lies within a helix that extends to A377 (white). Note that for simplicity, we show the ExPReM domain as a monomer, although it is almost certainly usually a dimer (see Fig. EV1A).

**Figure EV1:**
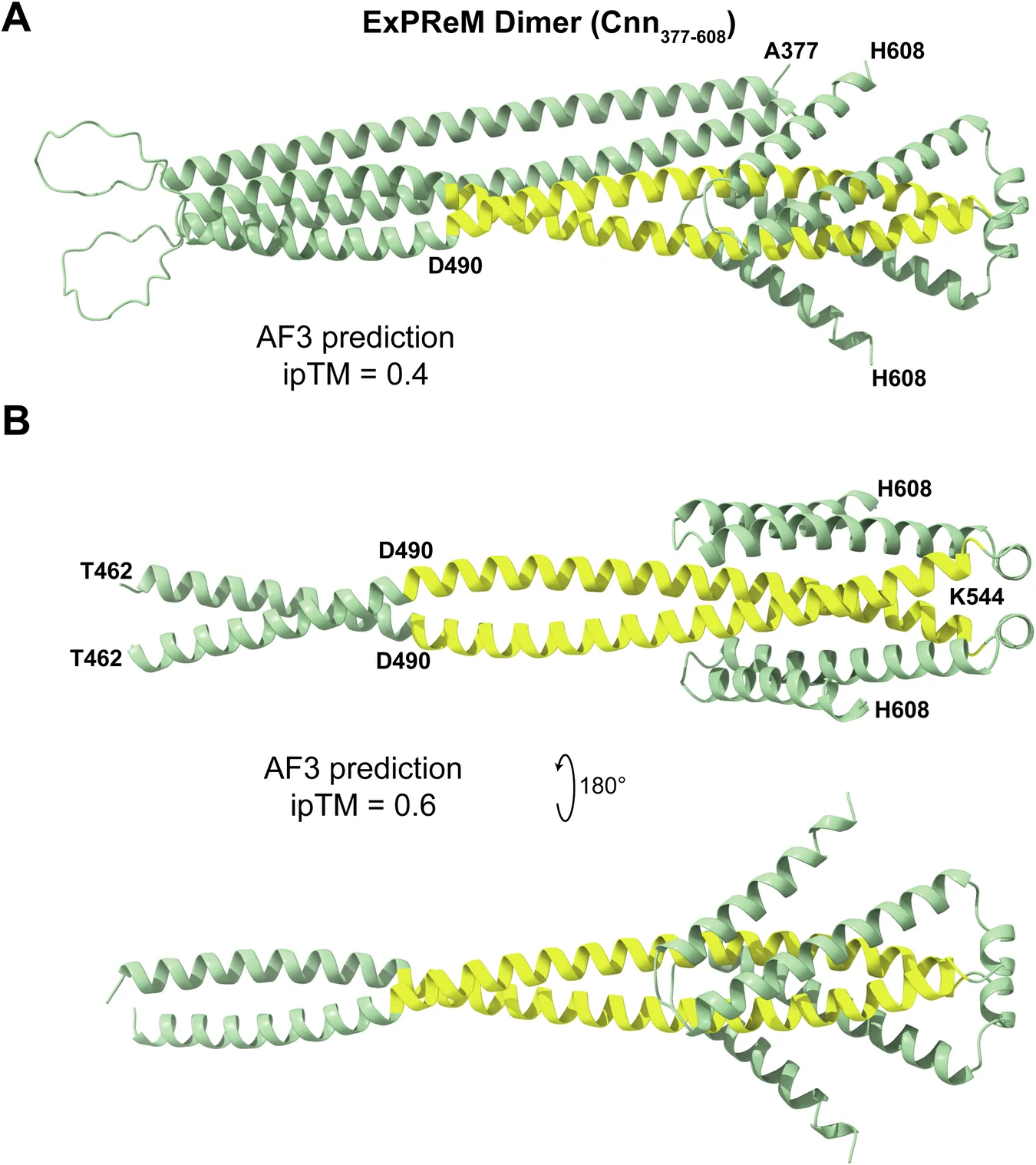
Predicted structures of PReM domain dimers. Ribbon representations of AF3 predictions of the Extended PReM (ExPReM) domain dimer (Cnn_377-608_) (**A**), and what appears to be the “core” helical hairpin structure of the PReM domain (Cnn_462-608_) (incorporating H2 + H3 + H4 from the ExPReM structure) (**B**). The LZ is highlighted in yellow, and additional helices in green. See also Movie EV1.

The original PReM domain (Cnn_403-608_) contains an internal leucine-zipper dimer (LZ; Cnn_490-544_), whose crystal structure in a tetrameric complex with a CM2 dimer has been solved (Fig. 1A,B) (Feng et al, 2017). To better understand how the additional sequences flanking the LZ enable the PReM and CM2 domains to form a PReM::CM2 macromolecular scaffold—rather than the simple tetramer formed by LZ::CM2—we designed constructs extending the LZ at its N- and/or C-termini. We successfully crystallised and solved the structure (to 1.96 Å resolution) of one such construct that extends the C-terminus of the LZ dimer by 35aa (LZ + ; Cnn_490-579_) (green helices, Fig. 1C). In this structure, the residues extending beyond the original LZ form helices that folded back onto the LZ dimer to form a helical hairpin. Despite extensive efforts, we were unable to generate additional informative constructs, so we turned to AlphaFold3 (AF3) for structural predictions (Abramson et al, 2024). AF3 generated a high-confidence prediction for LZ+ (ipTM = 0.73) that aligned closely with the crystal structure (86 Cα pairs with rmsd = 0.910 Å) (Fig. 1D). For the full PReM dimer, AF3 predicated a structure comprising several helical regions (ipTM = 0.4) (Fig. 1E). For clarity, we present the predicted PReM structure as a monomer to illustrate helix boundaries used in subsequent experiments (Fig. 1E). The predicted dimer structure (likely to be more biologically relevant) is shown in Fig EV1A.

AF3 predicted that the originally defined N-terminus of the PReM domain (Q403) lies within a longer helix that extends N-terminally to A377 (Helix H1, Cnn_377-443_) (Fig. 1E). Thus, the original PReM construct (Cnn_403-608_) (Feng et al, 2017) contained only ~2/3 of H1. Interestingly, H1 was not predicted to interact with the other helices in the PReM, suggesting that it does not contribute directly to PReM structural integrity. A second helix (H2, Cnn_462-544_) contains the original LZ (Cnn_490-544_—highlighted in yellow), followed by the helix observed in the LZ+ crystal structure (H3, Cnn_554-583_) and a fourth helix (H4, Cnn_589-602_ or Cnn_587-607_ depending on the input sequence). These 3 helices (H2-H4) formed a compact structural unit in which H3 and H4 fold back to interact with each other and with H2, generating a double helical hairpin (Fig. EV1B; Movie EV1).

### Defining the PReM domain regions required for scaffold assembly in vitro and in vivo

We previously showed that the LZ::CM2 interaction is important for the assembly of the PReM::CM2 scaffold in vitro and the endogenous Cnn scaffold in vivo (Feng et al, 2017). In addition, phosphorylation of the C-terminal region of the PReM—which corresponds to H3 and H4 of the helical hairpin—also appears to be critical for efficient scaffold assembly (Conduit et al, 2014a; Feng et al, 2017). Whether regions N-terminal to the LZ also contribute to these processes was unclear.

**Figure 2:**
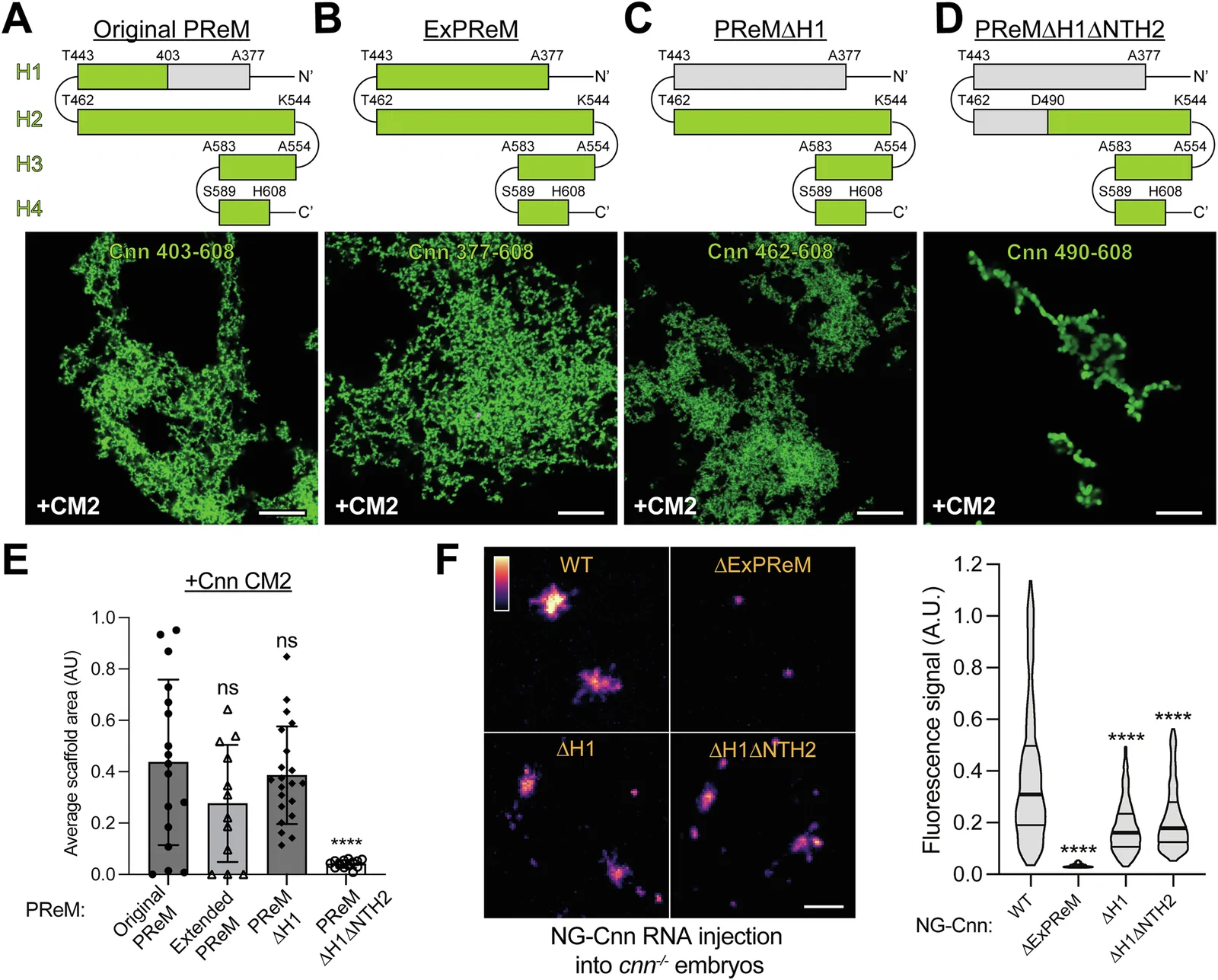
Analysis of PReM regions required for scaffold assembly in vitro and in vivo. (**A**–**D**) Images show a typical microscope field of the structures observed when NG-fusions of various PReM domain constructs—illustrated schematically above each image, with the region included in each construct shaded green—were mixed with CM2 in vitro. (**E**) Graph quantifies the area of NG-structure observed by microscopy for each construct shown in (**A**–**D**) (mean ± SD). Each point on the graph represents the average scaffold area in a region comprising 100 individual images; at least four different regions of a coverslip were sampled in this way, and the data pooled from three different coverslips. Statistical significance was assessed using one-way ANOVA with Dunnett’s multiple comparison test in GraphPad Prism (ns  = not significant: not significant; ****P < 0.0001). Scale bar =  10 μm. (**F**) Images illustrate, and violin plot quantifies, the in vivo centrosomal fluorescence intensity (bars indicate mean ± first and third quartile) when mRNA encoding a NG-fusion of each construct was injected into *cnn*^*−/−*^ embryos. *N* = 12–15 embryos; *n* = ~250–750 centrosomes per construct. Statistical significance was assessed using a one-way ANOVA analysis with Kruskal–Wallis test in GraphPad Prism (****P < 0.0001). Scale bar (**A**–**D**) = 10 μm; (**F**) = 2 μm.

**Figure EV2:**
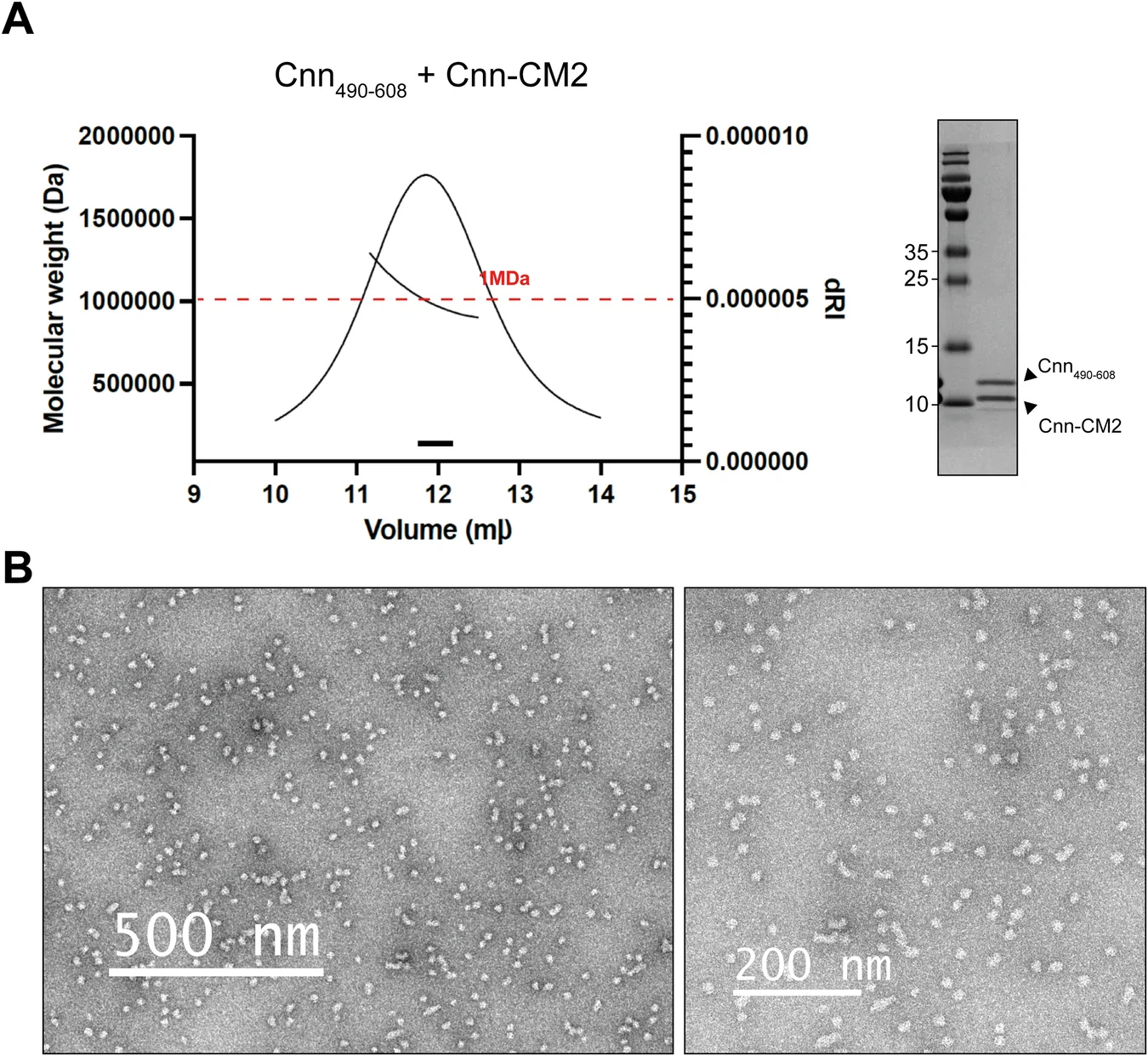
Cnn_490-608_ forms high MW (~1 MDa) complexes when mixed with CM2, rather than extended scaffolds. (**A**) Traces of a SEC MALS experiment (the fraction highlighted by the black bar is shown on the associated SDS gel) showing that Cnn_490-608_ forms high MW complexes of ~ 1 MDa when mixed with the CM2 domain in vitro. (**B**) Transmission EM images of the Cnn_490-608_::CM2 complexes.

To address this, we purified an N-terminally Extended PReM construct containing all of H1 (ExPReM, Cnn_377-608_). Monomeric-NeonGreen (NG) fusions of both the Original-PReM (Cnn_403-608_) and ExPReM formed macromolecular scaffolds when mixed with CM2 in vitro (Fig. 2A,B,E). Strikingly, the deletion of H1 (PReM∆H1, Cnn_462-608_) did not detectably impair scaffold formation (Fig. 2C,E). In contrast, further deletion of the N-terminal third of H2 (PReM∆H1∆NTH2, Cnn_490-608_), leaving only the LZ region intact, substantially altered assembly behaviour. Although large PReMΔH1ΔNTH2::CM2 complexes still formed, these appeared as aggregated particles rather than extended scaffolds (Fig. 2D). These particles had an average mass of ~1 MDa (Fig. EV2A) and could be visualised by negative stain EM (Fig. EV2B), but they lacked obvious structure and were not suitable for single particle Cryo-EM analysis. Together, these results indicate that H1 is dispensable for PReM::CM2 scaffold assembly in vitro, whereas removal of the N-terminal portion of H2 disrupts scaffold organisation even when the LZ and C-terminal hairpin remain intact.

To assess the in vivo relevance of H1, we injected mRNA encoding either WT or H1-deleted Cnn (Cnn∆H1) into embryos laid by *cnn*^*−/−*^ mothers lacking endogenous Cnn (*cnn*^*−/−*^ embryos) (Fig. 2F). As expected, deletion of the entire ExPReM region (Cnn-∆ExPReM) strongly suppressed scaffold assembly. In contrast, deletion of H1 alone (Cnn-∆H1) produced only a modest, though still significant, reduction in scaffold formation. Perhaps surprisingly, additional deletion of the N-terminal third of H2 (Cnn-∆H1∆NTH2) did not exacerbate this phenotype, producing a similar effect to the deletion of H1 alone. Together with published data (Conduit et al, 2014a; Feng et al, 2017), these data highlight the importance of the C-terminal helical hairpin region of the PReM for physiological Cnn scaffold assembly.

### The PReM helical hairpin appears to autoinhibit Cnn scaffold assembly

**Figure 3:**
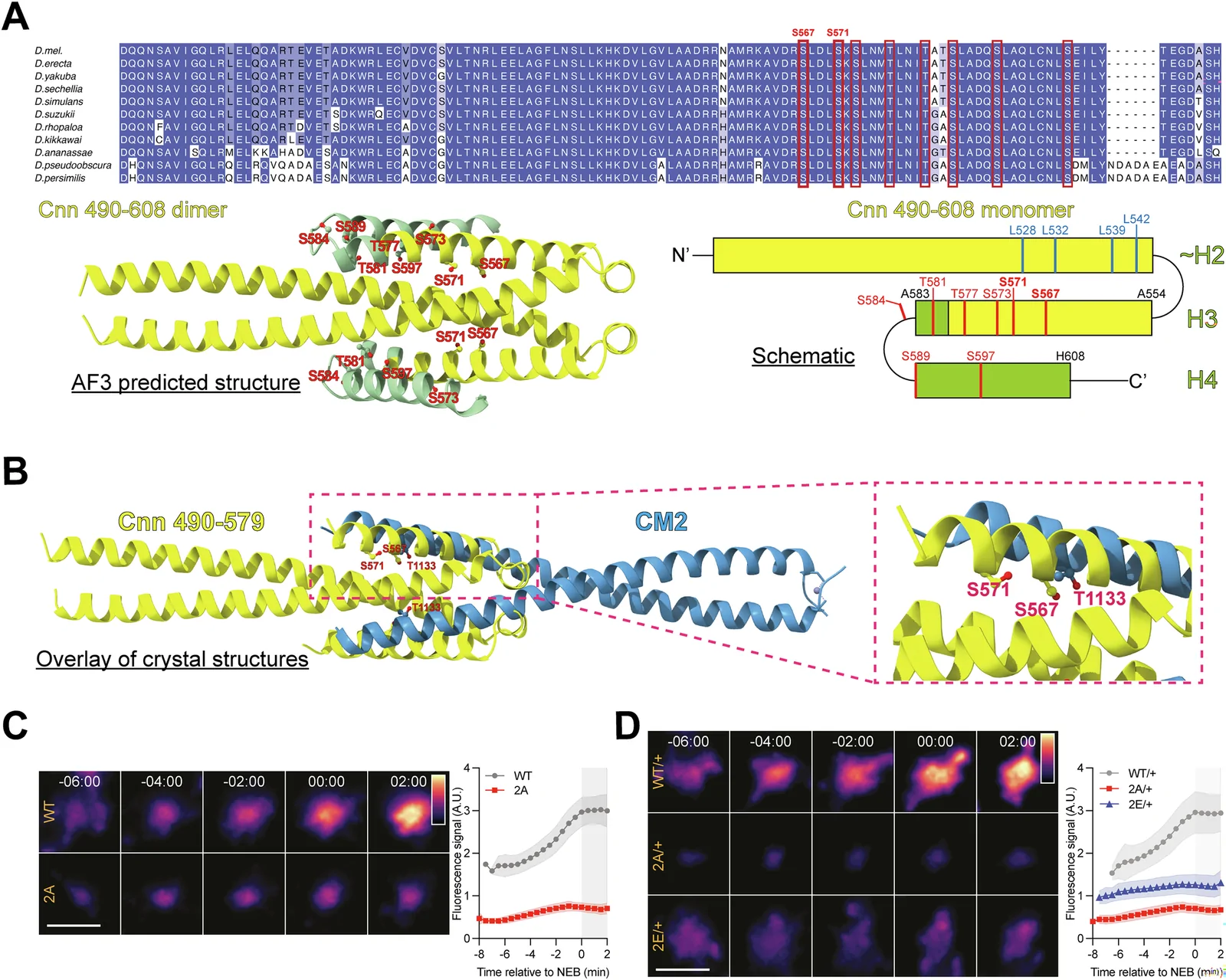
The PReM hairpin prevents CM2 binding, but this may be relieved by the phosphorylation of S567 and/or S571. (**A**) Multiple sequence alignment (top) of the PReM domain (H2-H4) from several *Drosophila* species, highlighting conserved Ser/Thr residues within the helical hairpin region (red boxes). These residues are also highlighted (red) in the ribbon diagram and schematic of the AF3-predicted structure (bottom). In these illustrations, crystal structures are shown in yellow, and the extended regions predicted by AF3 in green. Residues in the LZ that are important for the CM2 interaction are highlighted in the schematic in blue. (**B**) An overlay of the crystal structures of the LZ+ helical hairpin (Cnn_490-579_; yellow), and the CM2 domain (blue) taken from the LZ::CM2 crystal structure. The binding of H3 to H2 in LZ+ occludes the binding site on H2 for CM2. The inset shows the position of S567 and S571 within the H2::H3 PReM interface and of T1133 in the PReM::CM2 interface. (**C**, **D**) Images illustrate, and graphs quantify, the centrosomal fluorescence intensity (mean ± SD) of WT NG-Cnn or NG-Cnn-2A expressed in *cnn*^*−/−*^ embryos (**C**) or WT NG-Cnn, NG-Cnn-2A or NG-Cnn-2E laid by *cnn*^*+/−*^ heterozygous females (so the embryos contain some endogenous unlabelled WT Cnn) (**D**) during nuclear cycle 12. Time (min) relative to the start of mitosis (*t* = 0) is indicated on the *x* axis. *N* = 12 embryos; *n *= ~725–880 centrosomes analysed for each construct. Scale bars = 2 μm.

The helical hairpin C-terminal to the original LZ contains 8 conserved Ser/Thr residues whose surrounding amino acid context at least partially matches the minimal PLK1 consensus phosphorylation motif (N/D/E-X-S/T-Φ, where S/T is the phosphorylated Ser/Thr and Φ indicates a hydrophobic amino acid) (Nakajima et al, 2003; Santamaria et al, 2011) (red boxes, Fig. 3A). In vivo, mutating combinations of these residues to Ala disrupts Cnn scaffold assembly to varying degrees, suggesting that phosphorylation at multiple sites contributes to Cnn scaffold assembly (Conduit et al, 2014a). We therefore examined how phosphorylation might influence the structure and function of the helical hairpin.

Overlaying the crystal structure of the PReM helical hairpin (Cnn_490-579_), with our previous structure of the PReM-LZ bound to CM2 revealed that the folding-back of H3 occludes the normal CM2-binding site on H2 (Fig. 3B). Moreover, S567 in H3 occupies a position closely resembling that of the highly conserved T1133 in the CM2- bound complex (inset, Fig. 3B). This is striking, as a T1133E substitution in CM2 essentially abolishes the PReM::CM2 interaction in vitro and Cnn scaffold assembly in vivo (Feng et al, 2017), indicating that phosphorylation of the similarly positioned S567 within the PReM helical hairpin would likely disrupt the hairpin fold. These structural observations suggest that the H2/H3 hairpin normally sterically inhibits CM2 binding, and that phosphorylation of S567—and potentially the nearby S571, which is also buried at the hairpin interface—would disrupt this interaction, potentially “opening” the hairpin to allow CM2 binding to initiate scaffold assembly.

**Figure EV3:**
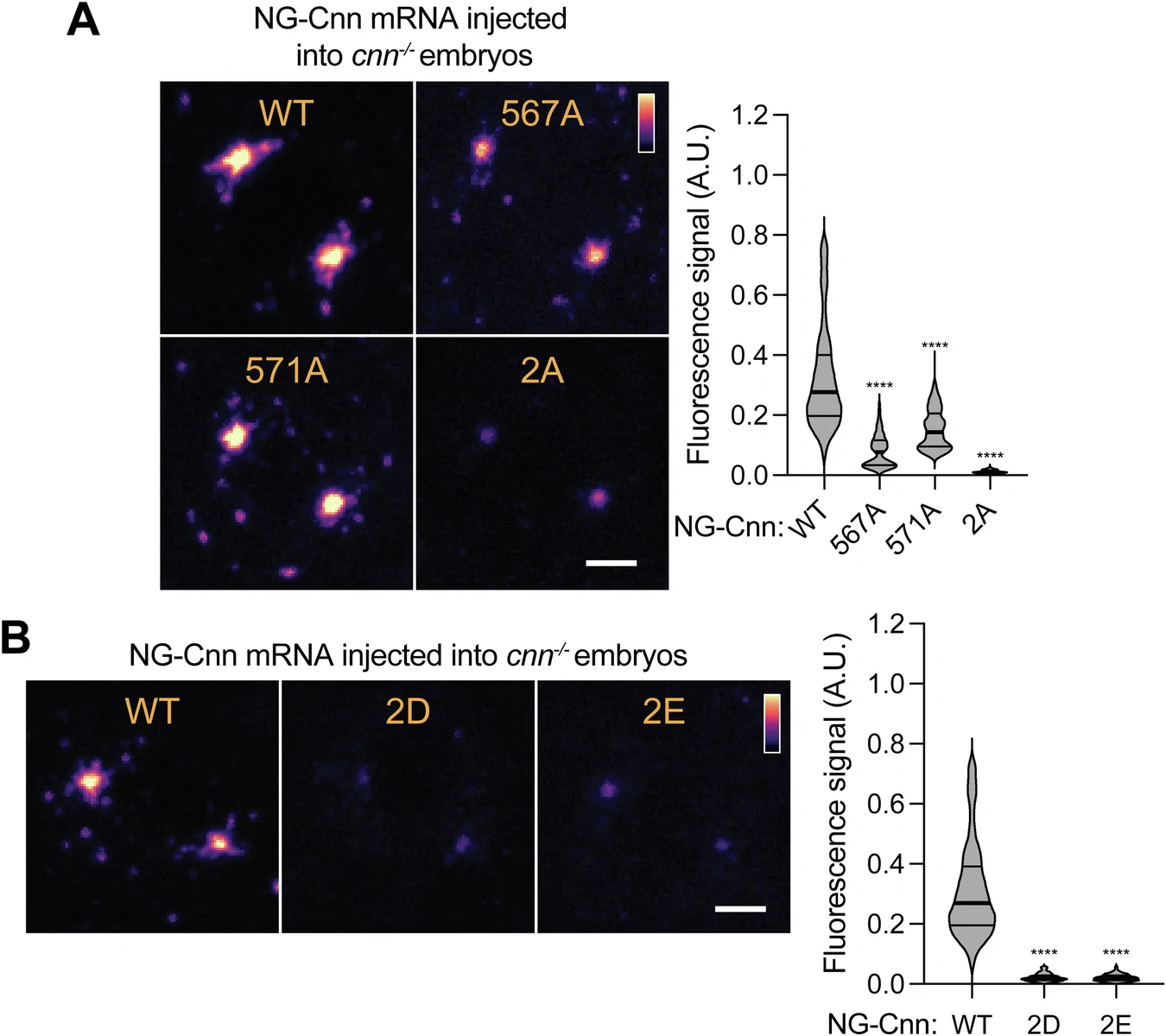
Single S567A and S571A substitutions perturb Cnn scaffold assembly in vivo. Images illustrate, and violin plots quantify, the in vivo centrosomal fluorescence intensity (mean±first and third quartile) of NG-fusions of WT, S567A, S571A or S567A,S571A (2A) Cnn proteins expressed from mRNAs injected into *cnn*^*−/−*^ embryos (**A**) or WT, S567D,S571D (2D), or S567E,S571E (2E) proteins expressed from mRNAs injected into embryos laid by heterozygous *cnn* mutant females (*cnn*^*+/−*^ embryos) (**B**). Note that image brightness was adjusted slightly differently for the 567A, 2A, 2D, and 2E constructs to allow visualisation of centrosomes, which were otherwise too dim to be clearly visualised. Statistical significance was assessed using a one-way ANOVA analysis with Dunnett’s multiple comparison test in GraphPad Prism (****P < 0.0001). *N* = > 10 embryos, *n* = >500 centrosomes for each NG-fusion. Scale bar = 2 μm.

**Figure EV4:**
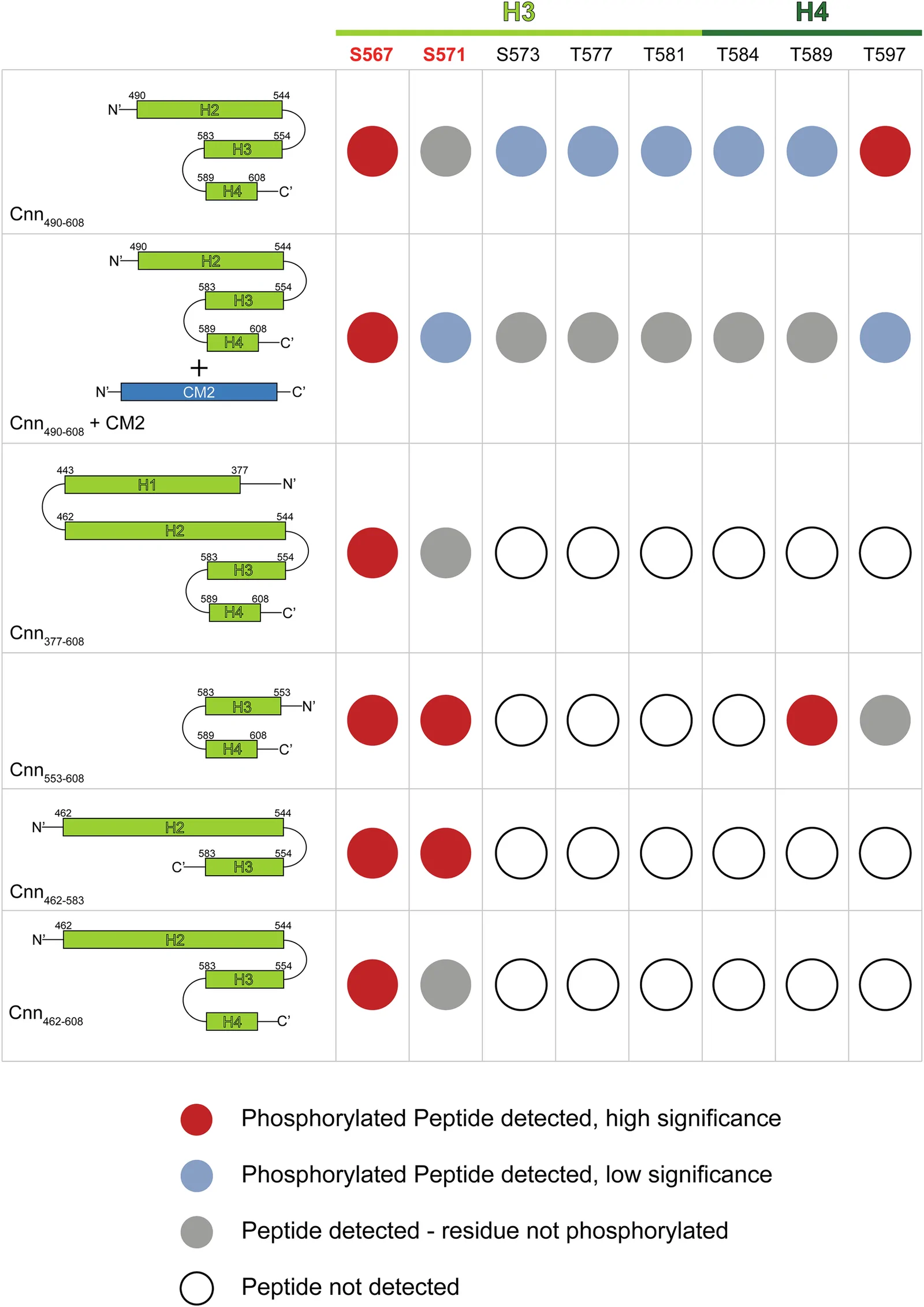
Summary of Mass Spec (MS) analysis of in vitro Polo-dependent phosphorylation of PReM domain constructs. Schematics on the left indicate the recombinant constructs that were mixed with purified *Drosophila* Polo kinase and then analysed by MS to identify phosphorylated residues. The position of the conserved Ser/Thr residues in the C-terminal region of the PReM (containing helix H3 and H4) are indicated above (S567 and S571, the most relevant for this study, are highlighted in red), and a coloured dot indicates the result of the MS analysis. Red dot indicates the phosphorylated peptide was identified with high significance; blue dot indicates the phosphorylated peptide was identified but with low significance; grey dot indicates that the peptide was identified with high significance, but it was not phosphorylated. No dot indicates that no peptide covering the residue was identified.

To test this hypothesis, we generated a double alanine mutant (Cnn-2A; S567A/S571A) and expressed NG-Cnn-2A in *cnn*^*−/−*^ embryos. As reported previously (Wong et al, 2022, 2024), WT-NG-Cnn levels accumulated at centrosomes in the run-up to mitosis, and peaked during mitosis (Fig. 3C). In contrast, NG-Cnn-2A still localised to centrosomes but exhibited a dramatic reduction in scaffold assembly (Fig. 3C). Single mutants (Cnn-S567A and Cnn-S571A) also impaired scaffold assembly although not as severely as the double mutant, with the S567A mutation having the greatest effect (Fig. EV3A). We previously showed with phospho-specific antibodies that Cnn-S567 is phosphorylated in vivo on Cnn molecules that incorporate into the centrosomal scaffold (Feng et al, 2017). Phospho-specific antibodies for S571 are not available, but in vitro phosphorylation assays with various PReM constructs using recombinant *Drosophila* Polo revealed consistent phosphorylation of S567 and detected phosphorylation of S571, although less consistently and less significantly (Fig. EV4; see “Methods” for links to the raw MS data). Together, these data support a model in which Polo-dependent phosphorylation of S567, and to a lesser extent S571, promotes the opening of the PReM helical hairpin, thereby enabling CM2 binding and scaffold assembly.

### S567/S571 phosphorylation appears to open the helical hairpin to allow CM2 binding

**Figure 4:**
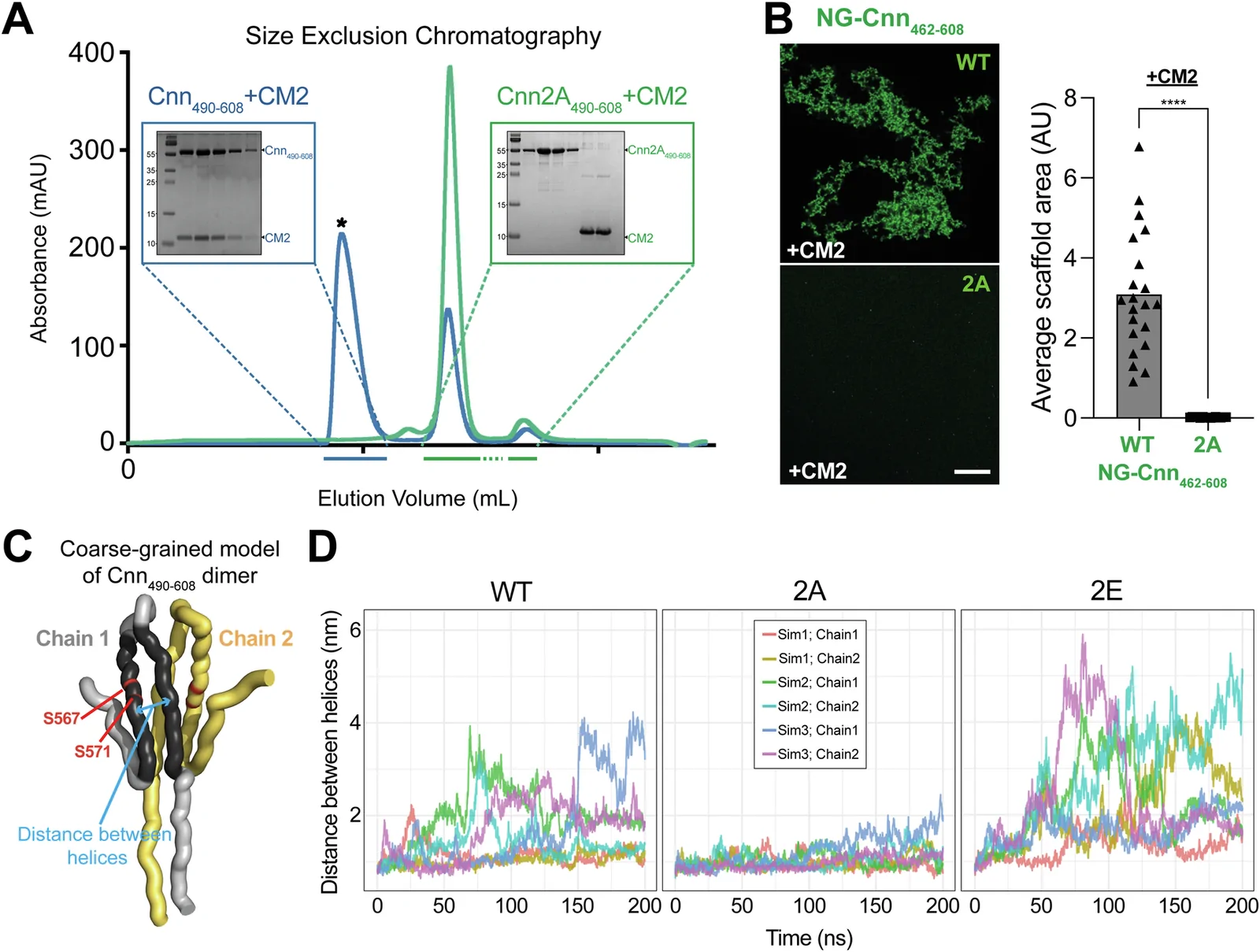
Cnn-2A mutations block the PReM::CM2 interaction and appear to lock the PReM helical hairpin in a closed conformation. (**A**) Panel shows traces from size-exclusion chromatography experiments (insets show the accompanying SDS-gels from the fractions indicated by the solid lines underneath each trace) illustrating the behaviour of the WT (blue) and 2A-mutant (green) PReM domains (Cnn_490-608_) when mixed with CM2 in vitro. WT PReM forms high-MW complexes with CM2 that elute in the void volume (asterisk), whereas the 2A mutant does not bind to CM2. (**B**) Images show a typical microscope field of the structures observed when NG-fusions of WT or 2A PReM domains (Cnn_462-608_) were mixed with CM2 in vitro. The bar-chart quantifies the area of scaffold observed by microscopy for each construct (mean ± SD). Each point on the graph represents the average scaffold area in a region comprising 100 individual images; at least four different regions of a coverslip were sampled, and the data were pooled from three different coverslips. Statistical significance was assessed using an unpaired Mann–Whitney *t* test in GraphPad Prism (****P < 0.0001). Scale bar = 10 μm. (**C**) Schematic illustration of the coarse-grain model of the PReM (Cnn_490-608_) helical hairpin dimer, highlighting the position of S567 and S571 and the point where the distance between the H2 and H3 helices in the hairpin structure was measured during molecular simulations. (**D**) Graphs plot the distance between the H2 and H3 helices during three separate simulations of 200 ns. This generated the six traces shown, as the distance between the helices was calculated for both chains of the dimer independently.

**Figure EV5:**
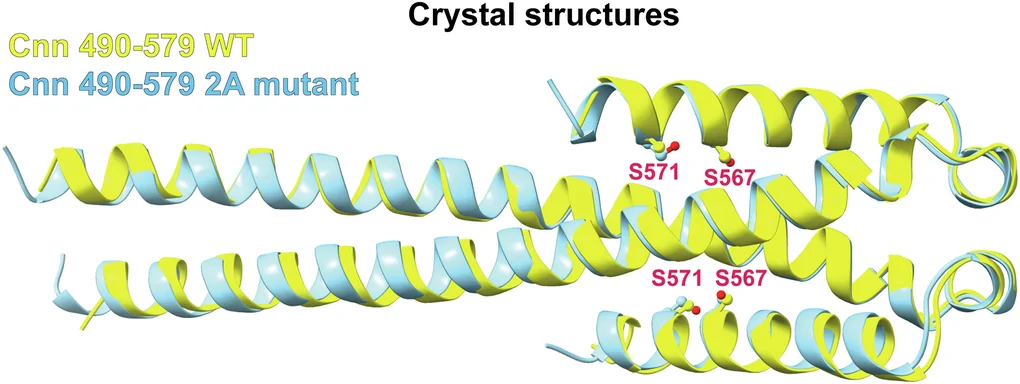
Cnn-2A mutations do not perturb the helical hairpin fold of the LZ+ domain. An overlay of the crystal structures of the WT (yellow) and 2A mutant (blue) Cnn_490-579_ LZ+ domains reveals that the mutations do not significantly alter the helical hairpin structure.

Although Polo/PLK1 phosphorylation enhances the PReM::CM2 interaction in vitro, unphosphorylated PReM can still bind CM2, suggesting that the non-phosphorylated hairpin can transiently “breathe” to allow CM2 binding—at least at these high concentrations in vitro (Feng et al, 2017). Unexpectedly, however, we detected no interaction between CM2 and the Cnn-2A_490-608_ (Fig. 4A), and Cnn-2A_462-608_ failed to form scaffolds when mixed with CM2 in vitro (Fig. 4B). Crystal structures of the Cnn_490-579_ WT and 2A mutants were nearly identical (81 Cα pairs, rmsd = 0.72 Å) (Fig. EV5), indicating that the double alanine substitutions do not disrupt the hairpin fold. We therefore hypothesised that the 2A substitutions might alter the intrinsic dynamics of the helical hairpin, stabilising the closed conformation and preventing the breathing that normally allows CM2 binding in vitro.

To assess this possibility, we performed coarse-grained molecular simulations with WT, 2A and phosphomimetic 2E (S567E/S571E) versions of Cnn_490-608_ constructs. In the coarse-grained representation, groups of ~3–4 heavy atoms are represented by a single “bead”, and an elastic network between backbone beads is applied to preserve the overall fold. This simplifies atomic interactions and constrains large-scale motions, so these simulations are interpreted in terms of relative breathing behaviour between PReM variants rather than atomistic mechanisms. Hairpin breathing was quantified by measuring the distance between the centres of H2 and H3 over time (Fig. 4C). WT PReM exhibited substantial fluctuations, consistent with breathing behaviour, whereas the 2A mutant showed markedly reduced dynamics; in contrast, the 2E mutant exhibited increased breathing relative to WT (Fig. 4D). These simulations suggest that the inability of PReM-2A to bind CM2 arises from excessive stabilisation of the closed hairpin conformation, and that the hairpin can be destabilised by phosphomimicking 2E mutations.

**Figure EV6:**
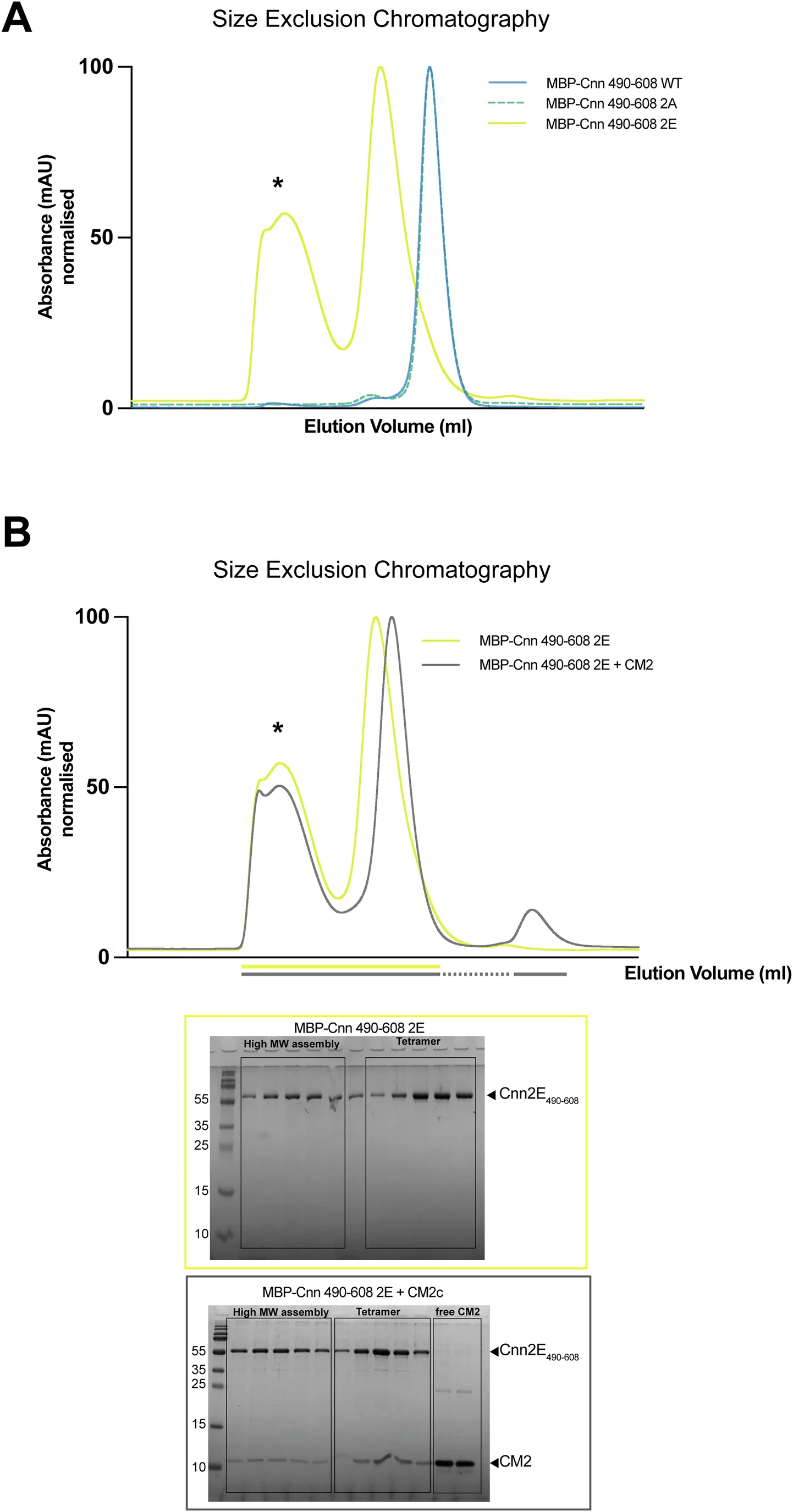
Size-exclusion chromatography analysis of MBP-Cnn_490–608_ 2A and 2E variants and their interaction with CM2. (**A**) Graph shows overlay of size-exclusion chromatography (SEC) profiles of WT (blue), 2A (dotted green) and 2E (yellow) variants of MBP-Cnn_490-608_. As shown previously, the WT protein elutes as a single well-behaved dimer species (Feng et al, 2017), and we found the 2A mutant behaved similarly. In contrast, the 2E mutant exhibits two main peaks: a high-molecular-weight peak that elutes close to the void volume (asterisk) and a second peak that runs close to the WT and 2A dimer peak, but is shifted slightly towards earlier elution volumes (indicating a higher molecular weight than a dimer—probably a tetramer). We conclude that the 2E substitutions allow MBP-Cnn_490-608_ to interact with itself in ways that the WT and 2A proteins do not. (**B**) Graph shows overlay of SEC profiles of MBP–Cnn_490-608_ 2E either alone (yellow)—same data as shown in (**A**)—or when incubated with CM2 (black). Insets below show SDS-gels of selected fractions from the elution profiles (as indicated by the solid lines underneath each SEC trace). The addition of CM2 alters the elution profile of MBP–Cnn_490-608_ 2E slightly. The void peak largely overlaps with the peak of the 2E protein alone, although the SDS-gel indicates that the CM2 protein is also incorporated into these higher molecular species. The “tetramer” peak also contains CM2 but is shifted slightly towards later elution volumes. This indicates a lower molecular weight than the postulated 2E tetramer (probably a 2E::CM2 tetramer). These data show that, in contrast to the 2A mutations, the 2E mutations do not prevent binding to CM2, and they are consistent with the possibility that the 2E mutations help to “open” the hairpin to promote self-interactions. For ease of comparison, absorbance at 280 nm is shown normalised in all graphs.

We therefore examined the behaviour of putative phospho-mimicking 2E mutants. In vitro, WT Cnn_490-608_ and Cnn-2A_490-608_ behaved predominantly as dimers, but Cnn-2E_490-608_ behaved as a mix of tetrameric and much larger species (that eluted in the void volume), indicating self-assembly even in the absence of CM2 (Fig. EV6A). In the presence of CM2, Cnn-2E_490-608_ formed tetrameric complexes and larger species containing CM2 that eluted similarly to the high-molecular-weight species observed for Cnn-2E_490-608_ alone (Fig. EV6B). These results are consistent with the possibility that the 2E substitutions open the hairpin to expose interaction surfaces that can promote PReM assembly into larger species even in the absence of CM2.

**Figure EV7:**
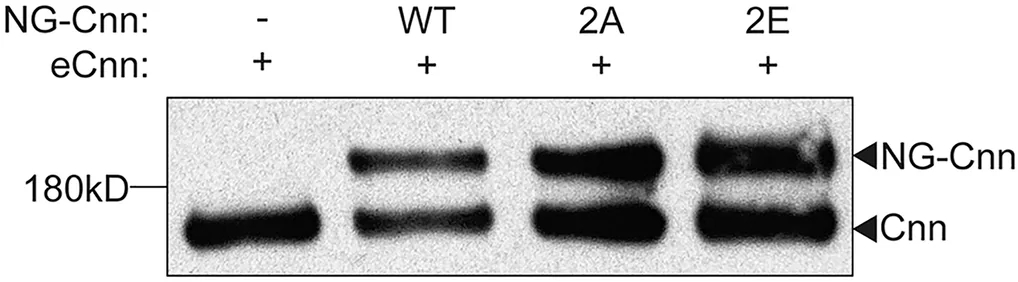
Cnn2A and Cnn2E are expressed at similar levels to WT Cnn. Western blot compares the expression levels of endogenous Cnn and the WT, 2A and 2E NG-fusions of Cnn in 0–2 h embryos laid by females expressing one copy of endogenous Cnn and one copy of the NG-fusion transgene. All the NG-fusions are present in embryos at similar levels to the endogenous protein.

In vivo, expression of full-length NG-Cnn-2E was dominant male sterile, preventing analysis in a homozygous *cnn*^*−/−*^ background. We therefore compared WT NG-Cnn-2E with NG-Cnn-2A and WT NG-Cnn in embryos containing one copy of endogenous (untagged) Cnn (Fig. EV7). Under these conditions, NG-Cnn-2E exhibited an intermediate phenotype, incorporating into centrosomal scaffolds more effectively than NG-Cnn-2A, but less efficiently than WT NG-Cnn (Fig. 3D). We observed a similar result when a potentially phospho-mimicking NG-Cnn-2D mutant was injected into *cnn*^*−/−*^ embryos (Fig. EV3B). These data suggest that the 2E and 2D substitutions do not fully mimic physiological phosphorylation and, although they appear to help open the helical hairpin, they cannot fully support Cnn scaffold assembly in vivo.

### The CM2 domain of human CDK5RAP2, but not worm SPD-5, can partially substitute for the fly CM2 domain

**Figure EV8:**
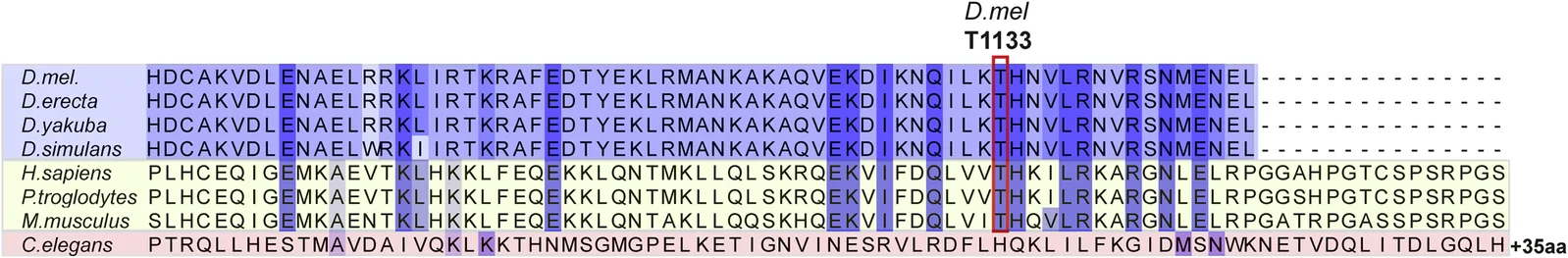
The *Drosophila* CM2 domain shows sequence homology to the CM2 domain of human CDK5RAP2, but not to the putative CM2 domain of worm SPD-5. A multiple sequence alignment of the CM2 (and putative CM2) domains of Cnn, CDK5RAP2 and SPD-5 from various species (*Drosophila* species boxed in blue, mammalian species in yellow and *C. elegans* in pink). The conserved Thr (T1133 in *Drosophila*) is highlighted (red box); in *Drosophila*, T1133 binds to the PReM in the same region that is occluded by S567 in the PReM helical hairpin structure (Fig. 4B), with S567 phosphorylation seeming to open the helical hairpin to allow CM2 to bind instead.

**Figure 5:**
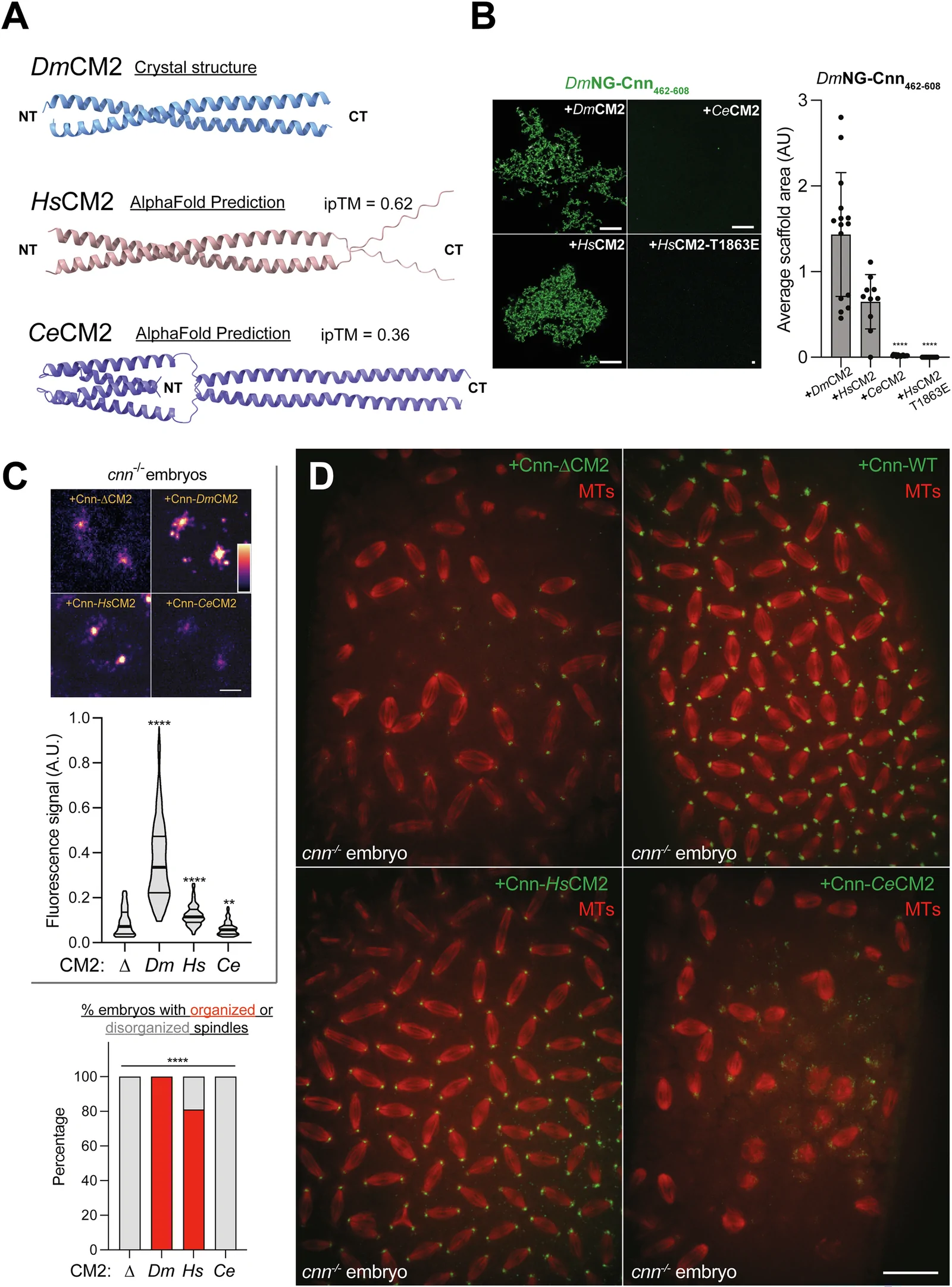
Human CDK5RAP2 CM2 can partially substitute for *Drosophila* Cnn CM2 in scaffold assembly in vitro and in vivo. (**A**) Crystal structures or AF3 predictions of *Drosophila*, Human or *C. elegans* CM2 dimers. (**B**) Images show a typical microscope field of the structures observed when fly (*Dm*), Human (*Hs*) (WT or T1863E mutant) or worm (*Ce*) CM2 were mixed with fly NG-PReM (Cnn_462-608_) in vitro; the bar-chart quantifies the area of scaffold observed by microscopy for each CM2 construct (mean ± SD). Statistical significance was assessed using an unpaired Mann–Whitney *t* test in GraphPad Prism (****P < 0.0001). Each point on the graph represents the average scaffold area in a region comprising 100 individual images; at least four different regions of a coverslip were sampled, and the data pooled from three different coverslips. Scale bar = 10 μm. Note that the *Hs*CM2-T1863E image is shown at a different magnification to capture a large field of view. (**C**) Images illustrate, and violin plot quantifies, the in vivo centrosomal fluorescence intensity (mean±first and third quartile) of each protein when mRNA encoding a NG-fusion of each construct was injected into *cnn*^*−/−*^ embryos. Statistical significance was assessed using a one-way ANOVA analysis with Kruskal–Wallis test in GraphPad Prism (****P < 0.0001; **P = 0.0031 < 0.01). N > 8 embryos; n > 400 centrosomes for each NG-fusion. (**D**) Images illustrate, and bar charts quantify, the ability of chimeric full-length Cnn molecules to rescue the embryonic defects in *cnn*^*−/−*^ embryos. These embryos exhibit a range of mitotic defects that were completely rescued by the injection of mRNA encoding Cnn containing the fly CM2 domain, partially rescued by Cnn containing the *Hs*CM2 domain, but were not detectably rescued by Cnn containing the *Ce*CM2 domain (*N* = 16 embryos analysed per construct). Images of embryos were randomised by one worker and analysed blind by another worker who had not previously seen any of the images but was familiar with the *cnn*^*−/−*^ embryo phenotype. Contingency significance (i.e., the significance of the difference in the proportion between the two groups) was calculated using a Fisher’s exact test (****P < 0.0001). Scale bar (**B**) = 10 μm; (**C**) = 2 μm; (**D**) = 20 μm.

Human CDK5RAP2 contains a C-terminal CM2 domain (Fig. EV8), and AF3 predicts that it adopts a dimeric structure similar to fly CM2 (Fig. 5A). In contrast, the putative CM2 domain of worm SPD-5 shows little sequence conservation (Ohta et al, 2021) (Fig. EV8) and is predicted by AF3 to adopt a distinct structure (Fig. 5A). Consistent with these predictions, both recombinant fly and human CM2 domains formed macromolecular networks when mixed with *Drosophila* PReM (NG-Cnn_462–608_), whereas worm CM2 did not (Fig. 5B). As discussed above, the *Drosophila* CM2-T1133E mutation abolishes PReM::CM2 scaffold assembly in vitro (Feng et al, 2017), and we found that the equivalent mutation in human CM2 (T1863E) also abolished *Dm*PReM::*Hs*CM2-T1863E scaffold assembly (Fig. 5B).

To test whether the human or worm CM2 domains could substitute for the fly CM2 domain in vivo, we generated chimeric NG-Cnn constructs. The fly–human–CM2 chimaera localised to centrosomes and substantially rescued centrosome function and embryonic development in *cnn*^*−/−*^ embryos, albeit less efficiently than WT Cnn (Fig. 5C,D). In contrast, the fly–worm–CM2 chimaera failed to localise properly or rescue function (Fig. 5C,D). Thus, human CM2 can cooperate with the *Drosophila* PReM to assemble scaffolds in vitro and in vivo, whereas worm CM2 cannot. Together, these data indicate that the fly Cnn-CM2 and human CDK5RAP2-CM2 domains function in a similar manner.

### Identification of a putative PReM domain in human CDK5RAP2

**Figure 6:**
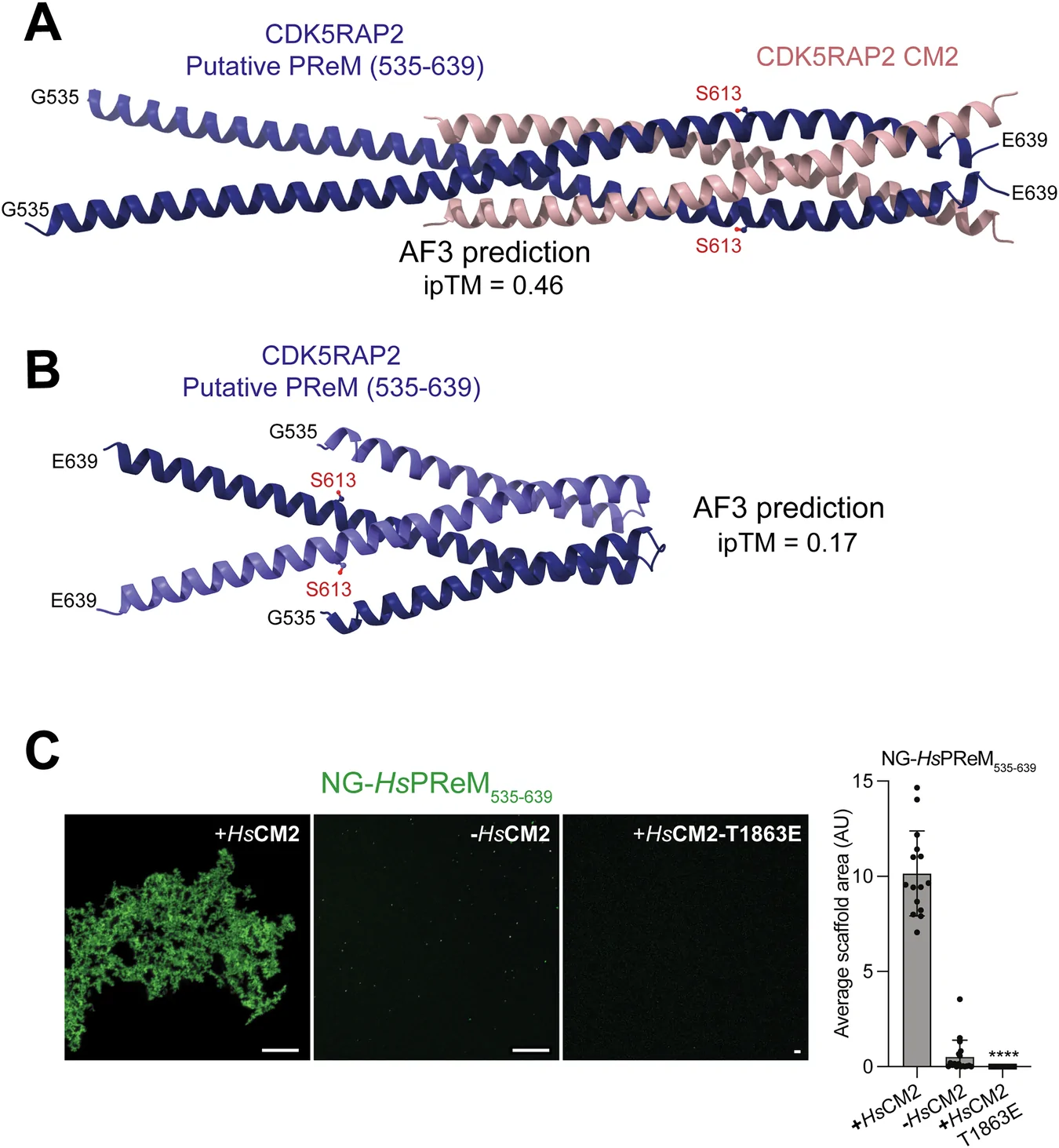
Identification of a potential PReM domain candidate in Human CDK5RAP2. (**A**, **B**) AF3 predictions of a potential PReM domain in Human CDK5RAP2 interacting with CM2 (**A**), and its potential folding as a helical hairpin dimer in isolation (i.e., when not bound to CM2) (**B**). S613, which is known to be phosphorylated in vivo and can be phosphorylated by PLK1 kinase in vitro, is highlighted (red). (**C**) Images show a typical microscope field observed with fluorescent NG-CDK5RAP2-PReM, either on its own or when mixed with non-fluorescent WT CDK5RAP2-CM2 or CDK5RAP2-T1863E in vitro. Graph quantifies the area of scaffold observed by microscopy for each condition (mean ± SD). Statistical significance was assessed using an unpaired Mann–Whitney *t* test in GraphPad Prism (****P < 0.0001). Each point on the graph represents the average scaffold area in a region comprising 100 individual images; at least four different regions of a coverslip were sampled, and the data were pooled from three different coverslips. Scale bar = 10 μm. Note that the *Hs*CM2-T1863E image is shown at a different magnification to capture a large field of view.

If this is the case, human CDKRAP2 should contain a functionally equivalent PReM domain. A recent study identified such a domain (CDK5RAP2_611-670_), but this did not detectably interact with CDK5RAP2 CM2 (Yoo et al, 2025). We therefore used AF3 to predict the plausibility of interactions between human CDK5RAP2-CM2 and all the other predicted coiled-coil domains within CDK5RAP2. This identified a candidate region (CDK5RAP2_535-639_) predicted to interact with CM2 in a manner somewhat reminiscent of the fly Cnn-PReM::CM2 interaction (Fig. 6A; ipTM = 0.46). In isolation, this putative PReM domain was predicted to adopt a helical hairpin structure, albeit with low confidence (ipTM  = 0.17) (Fig. 6B).

**Figure 7:**
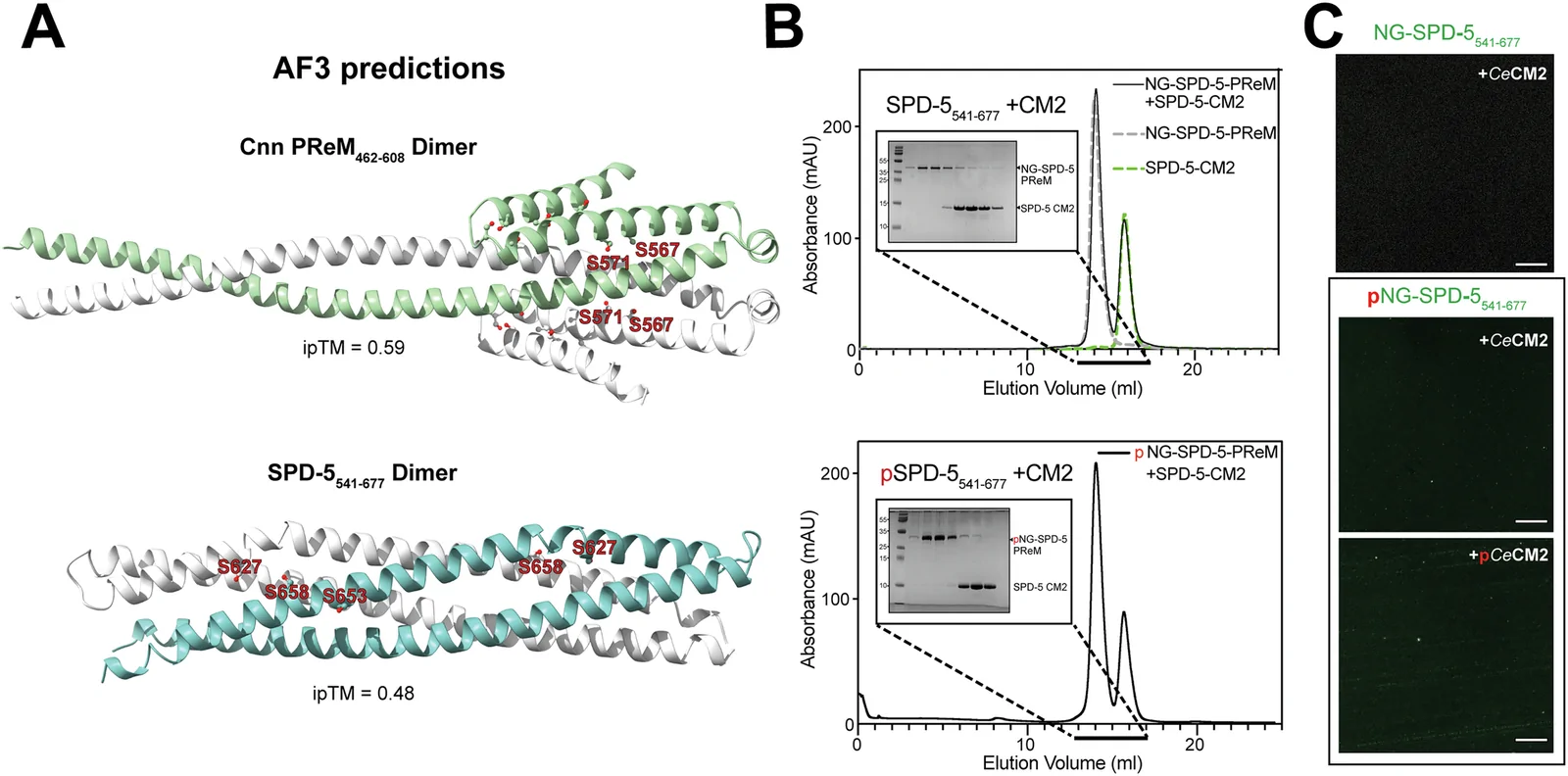
The putative PReM and CM2 domains in *C. elegans* SPD-5 do not detectably interact. (**A**) AF3 predictions of *Drosophila* Cnn or *C. elegans* SPD-5 PReM dimers. This region of SPD-5 is important for scaffold assembly in vivo, and the position of several Ser residues important for this function (S627, S653, S658) (Woodruff et al, 2015; Ohta et al, 2021; Rios et al, 2024) is indicated. Note that the previously predicted structure of the putative SPD-5 PReM (Rios et al, 2024) was generated by AF2/ColabFold, which predicted a parallel dimer, rather than the anti-parallel dimer shown here. We do not know the reason for this discrepancy, but note that the previous AF2 model is consistent with the X-linking MS data reported in that paper. Nevertheless, we show the AF3 prediction here to maintain consistency in our modelling (as all 5 top AF3 models predicted an anti-parallel dimer). (**B**, **C**) Panels show traces from size-exclusion chromatography experiments (insets show relevant SDS-gels) (**B**) or images from typical microscope fields (**C**) documenting the behaviour of NG-SPD-5-PReM_541-577_ when mixed with SPD-5-CM2 in vitro—in some cases after one or both proteins had been phosphorylated by recombinant worm PLK-1 kinase (indicated by red-p). MS analysis confirmed that PLK-1 phosphorylated the putative SPD-5 PReM domain on S627, S653 and S658 (Fig. EV9; see “Methods” for links to the raw MS data). Nevertheless, we detected no interaction between the SPD-5 putative PReM and Cnn domains. Scale bar  = 10 μm.

Intriguingly, this region contained several Ser/Thr residues and, although none of these was buried in the predicted interaction interface, it has previously been shown that S613 (highlighted in red in Fig. 7A,B) can be phosphorylated by recombinant PLK1 and that a phosphomimetic S613E substitution enhances condensate formation by full-length CDK5RAP2 in vitro (Rios et al, 2025). Moreover, in cells, S613 is phosphorylated specifically during mitosis, when centrosome scaffold assembly is occurring (Santamaria et al, 2011). Most importantly, we found that NG-CDK5RAP2_535-639_ formed large macromolecular structures when mixed with WT human CM2, and the assembly of these structures was abolished by the CM2-T1863E mutation (the equivalent of the *Drosophila* CM2-T1133E mutation) (Fig. 6C). This strongly suggests that the putative human CDK5RAP2 PReM and CM2 domains interact in a manner analogous to the equivalent fly domains.

### The worm SPD-5 PReM and CM2 domains do not detectably interact

**Figure EV9:**
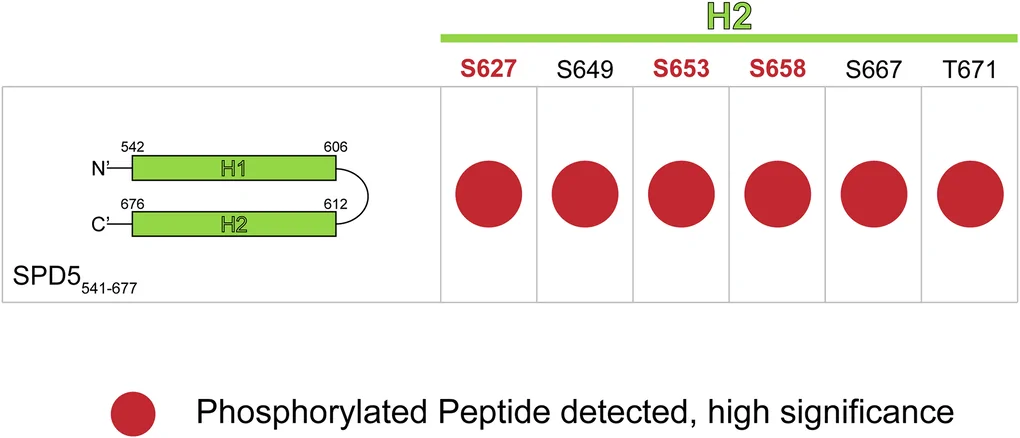
Summary of a Mass Spec (MS) analysis of the in vitro PLK-1-dependent phosphorylation of a worm SPD-5 PReM domain. The schematic on the left indicates the recombinant construct that was mixed with purified *C. elegans* PLK-1 kinase and then analysed by MS to identify phosphorylated residues. The position of Ser/Thr residues in the PReM is indicated above (S672, S635 and S658, whose phosphorylation has been shown to be important for SPD-5 scaffold assembly, are highlighted in red). The red coloured dot indicates peptides containing all of these phosphorylated Ser/Thr residues were identified with high significance.

Our data so far suggests that although the human PReM and CM2 domains appear to function in an analogous manner, this is not the case for the worm CM2 domain. We therefore analysed in more detail the putative worm SPD-5 PReM domain and its potential interaction with CM2. AF3 predicts that the worm SPD-5 putative PReM and fly Cnn PReM both form helical hairpins, but these structures differ markedly (Fig. 7A). Moreover, although the phosphorylation of these domains is clearly important for both Cnn and SPD-5 scaffold assembly (Conduit et al, 2014a; Woodruff et al, 2015; Wueseke et al, 2016; Ohta et al, 2021; Nakajo et al, 2022), none of the phosphorylation sites in the worm hairpin are buried in the predicted interaction interface in a manner analogous to S567 or S571. Consistent with this, we detected no interaction between worm PReM and CM2 domains in vitro, even after phosphorylation by PLK1 (Fig. 7B,C (Fig EV9—see “Methods” for links to the raw MS data)). This agrees with previous cross-linking MS data, which detected very few interactions between these domains (Rios et al, 2024). In sum, our results suggest that PReM–CM2 interactions are not structurally conserved between flies and worms.

### Full-length Cnn and SPD-5 form similar condensates but with distinct dynamics in vitro

The in vitro behaviour of full-length SPD-5 and CDK5RAP2 and of the PReM and CM2 domains of Cnn have been investigated in some detail (Woodruff et al, 2015, 2017; Feng et al, 2017; Rios et al, 2024, 2025). The ability of recombinant SPD-5 to form spherical condensates in vitro that can recruit other centrosomal proteins has been particularly influential in thinking about the biophysical nature of centrosomes (Woodruff et al, 2017). To date, however, no study has directly compared the behaviour of these recombinant proteins, nor assessed the importance of their PReM/CM2 domains for their interactions in vitro.

**Figure 8:**
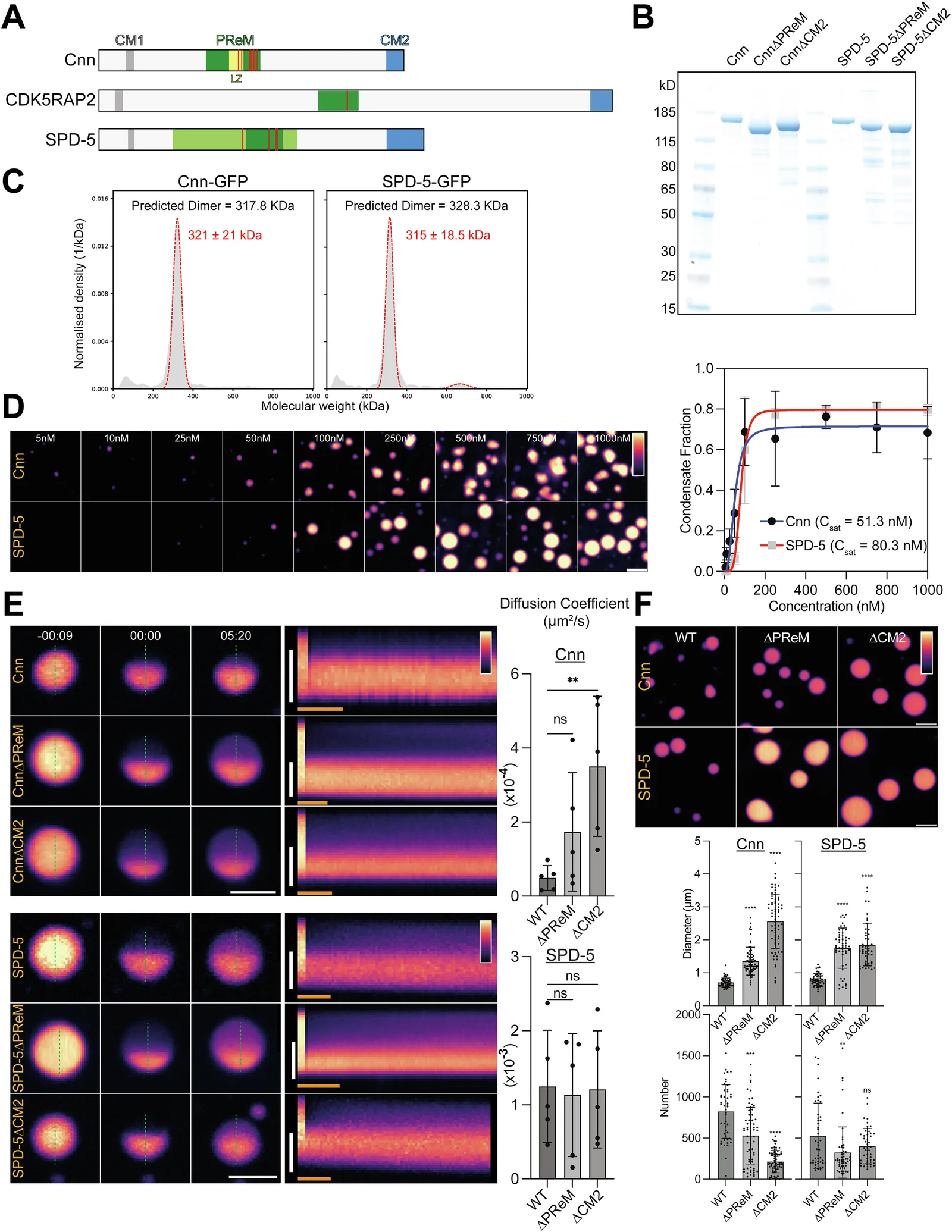
Analysis of full-length Cnn and SPD-5 behaviour in vitro. (**A**) Schematic illustration of Cnn, CDK5RAP2 and SPD-5, highlighting the position of the CM1 (grey), CM2 (blue) and putative PReM domains (green). The putative SPD-5 PReM domains identified by Nakajo et al, 2022 or Rios et al, 2024 are indicated in light green and dark green, respectively; red lines indicate potential phosphorylation sites. (**B**) Coomassie-stained gel of the purified Cnn and SPD-5 proteins analysed here. (**C**) A trace from a Mass photometry experiment showing the MW of the main species of full-length Cnn-GFP and SPD-5-GFP. A Gaussian model was fitted to obtain the molecular weight of the dimer (red dotted lines). (**D**) Images show the condensates formed by full-length Cnn-GFP and SPD-5-GFP at different concentrations; the graph quantifies the fraction of each protein within the condensate at each concentration (mean ± SD). A “Specific binding with Hill slope” equation (GraphPad Prism) was fitted, allowing the calculation of Csat; *n* = 6–20 measurements per concentration. (**E**) Images (left) and kymographs (right) from an experiment where the indicated condensates were half photobleached; graphs show calculated diffusion coefficients of fluorescent molecules moving across the bleached zone (mean ± SD). Statistical significance was assessed using a one-way ANOVA analysis with Kruskal–Wallis test in GraphPad Prism (Cnn WT vs ΔPReM **P = 0.0094 < 0.01, WT vs ΔCM2 ns = not significant: *P* = 0.3146; SPD-5 WT vs ΔPReM and WT vs ΔCM2 ns = not significant: *P* > 0.9999). *n* = 5 measurements per construct. (**F**) Images show, and graphs quantify the number and size of the condensates formed by full-length WT, ∆PReM and ∆CM2 versions of Cnn and SPD-5 at 2 μM (mean ± SD). Statistical significance was assessed using a one-way ANOVA analysis with Kruskal–Wallis test in GraphPad Prism (the number of Cnn condensates WT vs ΔPReM ***P = 0.0002 < 0.001, WT vs ΔCM2 ****P < 0.0001; the number of SPD-5 condensates WT vs ΔPReM **P = 0.0049 < 0.01, WT vs ΔCM2 ns = not significant: *P* > 0.9999). *n* = ~40–70 measurements per construct. Scale bar (**D**) = 2 μm; (**E**) = 2 μm (vertical), 50 s (horizontal).

We attempted to express and purify full-length Cnn, CDK5RAP2 and SPD-5 (Fig. 8A) using a baculovirus/insect-cell FlexiBac expression system (Lemaitre et al, 2019). We successfully purified Cnn and SPD-5 (Fig. 8B). CDK5RAP2 condensates have been studied in vitro previously (Rios et al, 2024), but we could not purify sufficient quantities of soluble CDK5RAP2 for our studies. Mass photometry revealed that Cnn and SPD-5 both behaved predominantly as dimers in solution, even at low nanomolar concentrations (Fig. 8C). In the presence of crowding agents (4% PEG, MW 20 kDa), both proteins formed spherical condensates (Fig. 8D), as reported previously for SPD-5 (Woodruff et al, 2017). We then performed half-compartment FRAP experiments to assess the diffusion of molecules within the condensates across the bleached zone. This analysis revealed that SPD-5 condensates were ~25-fold more dynamic than Cnn condensates (diffusion coefficients of 1.2 ± 0.8 × 10^−3^ and 4.9 ± 3.4 × 10^−5^ μm^2^/s, respectively) (Fig. 8E). This is consistent with previous reports that in vitro the Cnn PReM::CM2 scaffold (Feng et al, 2017) is less dynamic than the SPD-5 scaffold (Woodruff et al, 2017).

Perhaps surprisingly, the deletion of either the PReM or CM2 domains did not dramatically perturb the ability of either Cnn or SPD-5 to form condensates (Fig. 8F). Crucially, however, although the PReM- and CM2-deletions did not significantly alter the diffusion dynamics of SPD-5, they increased the dynamics of Cnn—by ~threefold and tenfold, respectively (although the increase in the PReM-deleted Cnn was not statistically significant) (Fig. 8E). Thus, although the PReM and CM2 domains are not strictly required for condensate formation in vitro, they appear to modulate the dynamic behaviour of the molecules within the Cnn condensates more than the molecules within the SPD-5 condensates.

## Discussion

The dramatic expansion of the PCM prior to mitosis is a conserved feature of centrosome behaviour (Palazzo et al, 2000; Conduit et al, 2015; Fernandes-Mariano et al, 2025). In both flies and worms, this expansion relies on Polo/PLK1-dependent phosphorylation of large coiled-coil proteins—Cnn and SPD-5, respectively—that can then assemble into extensive scaffolds that recruit additional components and confer mechanical integrity to the expanded PCM (Decker et al, 2011; Woodruff et al, 2015; Cabral et al, 2019; Ohta et al, 2021; Conduit et al, 2014a, 2014b). In flies, phosphorylation of the central PReM domain promotes interactions with the C-terminal CM2 domain to drive scaffold assembly (Feng et al, 2017). Here, we have dissected the structural basis of this regulation and tested whether similar mechanisms might operate in the equivalent worm and human proteins.

### The role of the PReM and CM2 domains in scaffold assembly

Our data support a model in which the *Drosophila* PReM adopts an autoinhibited helical hairpin conformation that occludes the CM2 binding site. Phosphorylation of S567 and S571—two residues buried at the hairpin interface—appears to disrupt this interaction, allowing the hairpin to open and enabling CM2 binding. Several lines of evidence support this model: (1) S567 is a major Polo phosphorylation site in vitro (with S571 also being phosphorylated, but less efficiently) and S567 is phosphorylated in vivo as Cnn incorporates into the centrosomal scaffold (Feng et al, 2017); (2) S567A and S571A substitutions impair scaffold *assembly* in vivo, and block CM2 binding in vitro; (3) Molecular dynamic simulations indicate that 2 A/2E substitutions stabilise/destabilise the closed hairpin, respectively.

Although phosphomimetic 2E substitutions appear to promote hairpin opening and enhance self-association in vitro, they only partially restore scaffold assembly in vivo. This indicates that the phosphorylation of these residues promotes scaffold assembly in a manner that is not fully mimicked by the 2E substitutions. Moreover, the phosphorylation of multiple additional Ser/Thr residues within the hairpin appears to contribute to robust scaffold assembly (Conduit et al, 2014a), and regions outside the hairpin—such as the N-terminal portion of H2 identified here—also have important roles. Thus, while phosphorylation of S567 and S571 may initiate Cnn scaffold assembly, full assembly requires additional phosphorylation within the PReM domain, as well as other interactions and regulatory inputs.

### Conservation and divergence of PReM/CM2 interactions

Human CDK5RAP2 contains a CM2 domain (Zhang and Megraw, 2007; Wang et al, 2010; Citron et al, 2018) that we show here can partially substitute for fly CM2 in PReM/CM2 scaffold assembly in vitro and Cnn scaffold assembly in vivo. We also identified a candidate PReM domain in human CDK5RAP2 that can assemble into a scaffold with human CM2 in vitro, and that contains a serine (S613) that is phosphorylated during mitosis in cells (Santamaria et al, 2011) and has been implicated in condensate formation in vitro (Rios et al, 2025). Moreover, the mutation of a highly conserved Thr within fly/human CM2 (T1133E/T1863E) abolishes PReM::CM2 in vitro scaffold assembly in both systems. These observations suggest that Cnn and CDK5RAP2 use similar PReM–CM2-dependent mechanisms to assemble a centrosomal scaffold.

In contrast, we found no evidence that the putative PReM and CM2 domains of worm SPD-5 interact in a comparable manner. The worm CM2 domain could not substitute for fly CM2 in scaffold assembly in vitro or in vivo, and we detected no interaction between the worm PReM and CM2 domains in vitro, even after phosphorylation by Polo/PLK-1—in agreement with previous cross-linking MS data (Rios et al, 2024). Both of these domains clearly play an important part in SPD-5 scaffold assembly in vivo (Woodruff et al, 2015; Ohta et al, 2021; Nakajo et al, 2022), but our data suggest that they do so using interactions that are structurally distinct from those used by the PReM and CM2 domains in flies and humans. We cannot rule out, however, that some other regions of worm SPD-5 interact in a manner that is more structurally analogous to the fly/human PReM and CM2 domains.

In the case of fly Cnn, our data are remarkably congruous. We have characterised the core of the PReM::CM2 interaction at the atomic level and shown that this interaction strongly influences the assembly of the Cnn scaffold in vivo (Feng et al, 2017). We now show here that these domains also strongly influence the dynamic behaviour of full-length Cnn condensates in vitro. This provides a rare example where the structure of an interaction that seems to contribute to condensate behaviour in vitro has been characterised at the atomic and macromolecular scale.

Importantly, the assembly of Cnn and SPD-5 condensates is not dramatically perturbed by the deletion of either the PReM or CM2 domains. It is only our analysis of diffusion rates within partially photobleached condensates that reveals that the deletion of the PReM or CM2 domains increases the diffusion rate of Cnn molecules within the condensates by ~threefold and ~tenfold, respectively. In contrast, the deletion of these putative domains in SPD-5 has little effect on condensate dynamics, further supporting our suggestion that these domains contribute to scaffold assembly in structurally different ways in flies and worms. This emphasises the need for biophysical analyses to assess condensate dynamics rather than relying simply on condensate assembly as a readout of phenotypes.

Our observation that both proteins can still form robust condensates in vitro without the PReM or CM2 domains suggests that additional interactions can support the in vitro self-assembly of both proteins under these conditions. These additional interactions may be physiologically relevant but are perhaps not strong enough to support efficient PCM assembly in vivo in the absence of the PReM or CM2 domains (for instance, in the presence of microtubule pulling forces). Alternatively, they may be non-physiological and arise from interactions promoted by the buffer conditions and crowding agents used in the assay. Further work will be needed to determine which features of the in vitro condensates reflect the organisation of the PCM scaffold in vivo.

Our data reveal both conservation and divergence in the mechanisms of PCM scaffold assembly. Flies and humans appear to rely on related PReM–CM2 interactions, whereas worms use distinct structural solutions to achieve a similar functional outcome. This evolutionary plasticity underscores the robustness of centrosomal scaffold assembly and highlights multiple molecular paths to building a mechanically resilient PCM.

### Polo/PLK1-dependent relief of autoinhibition as a general mechanism promoting centrosome assembly and function

Although the PReM::CM2 interaction does not appear to be conserved between fly Cnn and worm SDP-5, there is strong evidence that Polo/PLK-1-dependent phosphorylation helps to “open” a closed helical conformation of the PReM domain in both proteins—data from this study for Cnn, and the previously reported X-linking data for SPD-5 (Rios et al, 2024). Intriguingly, recent work indicates that the γ-TuC–binding region of SPD-5 also adopts an autoinhibited helical fold, and that Polo/PLK-1 phosphorylation is required both to relieve autoinhibition and to promote γ-TuC binding and activation (Ohta et al, 2026). The γ-TuC–binding region in Cnn—Conserved Motif 1 (CM1)—seems to behave analogously to SPD-5, requiring phosphorylation to relieve autoinhibition and permit γ-TuC engagement (Tovey et al, 2021), and this interaction is also autoinhibited in CDK5RAP2 (Yang et al, 2023).

Thus, these proteins may all employ a similar “double-lock” strategy, where phosphorylation removes inhibitory contacts *and* enhances productive interactions, to regulate γ-TuC binding/activation and scaffold assembly. Such a double-lock mechanism may be a conserved feature of Cnn/SPD-5/CDK5RAP2 proteins, and perhaps other centrosomal proteins. This helps to ensure that mitotic PCM assembly and MT nucleation occur preferentially at centrosomes, where Polo/PLK1 activity appears to be highest in the run-up to mitosis (Wong et al, 2022).

## Methods

**Table:** Reagents and tools table

Category	Reagent/resource	Reference or source	Identifier or catalogue number
Experimental Models	*Drosophila melanogaster* cnnf04547	Exelixis stock no. f04547, Exelixis Stock Centre (Harvard Medical School, Boston, MA)	Table EV1
Experimental Models	*Drosophila melanogaster* cnnHK21	Megraw et al, 1999; Vaizel-Ohayon and Schejter, 1999	Table EV1
Experimental Models	*Drosophila melanogaster* Ubq-NG-Cnn	Wong et al, 2022	Table EV1
Experimental Models	*Drosophila melanogaster* Ubq-NG-Cnn-2A	Generated in this study	Table EV1
Experimental Models	*Drosophila melanogaster* Ubq-NG-Cnn-2E	Generated in this study	Table EV1
Experimental Models	*Drosophila melanogaster* Jupiter-mCherry	Generated by the Daniel St Johnston lab	Table EV1
Experimental Models	cnnf04547/cnnHK21		Table EV2
Experimental Models	cnnf04547, Ubq-NG-Cnn/cnnHK21		Table EV2
Experimental Models	cnnf04547, Ubq-NG-Cnn-2A/cnnHK21		Table EV2
Experimental Models	Ubq-NG-Cnn/+		Table EV2
Experimental Models	Ubq-NG-Cnn-2A/+		Table EV2
Experimental Models	Ubq-NG-Cnn-2E/+		Table EV2
Experimental Models	cnnf04547/cnnHK21 ; Jupiter-mCherry/+		Table EV2
Experimental Models	*Escherichia coli* BL21 (DE3)	Used for recombinant protein expression in this study	
Experimental Models	Sf9 cells	Used for baculovirus-mediated full-length protein expression	
Experimental Models	Sf+ cells	Used for baculovirus-mediated full-length protein expression	
Recombinant DNA	pRNA-NG-Cnn	Generated in this study	Table EV4
Recombinant DNA	pRNA-NG-CnnΔ376–609	Generated in this study	Table EV4
Recombinant DNA	pRNA-NG-CnnΔ376–402	Generated in this study	Table EV4
Recombinant DNA	pRNA-NG-CnnΔ376–462	Generated in this study	Table EV4
Recombinant DNA	pRNA-NG-CnnΔ376–490	Generated in this study	Table EV4
Recombinant DNA	pRNA-NG-CnnΔ551–609	Generated in this study	Table EV4
Recombinant DNA	pRNA-NG-CnnΔ583–609	Generated in this study	Table EV4
Recombinant DNA	pRNA-NG-CnnΔCM2	Generated in this study	Table EV4
Recombinant DNA	pRNA-NG-Cnn-[CDK5RAP2::CM2]	Generated in this study	Table EV4
Recombinant DNA	pRNA-NG-Cnn-[SPD5::CM2]	Generated in this study	Table EV4
Recombinant DNA	pUbq-NG-Cnn-2A	Generated in this study	Table EV4
Recombinant DNA	pUbq-NG-Cnn-2E	Generated in this study	Table EV4
Recombinant DNA	pRNA-NG-Cnn-2A	Generated in this study	Table EV4
Recombinant DNA	pRNA-NG-Cnn-2E	Generated in this study	Table EV4
Recombinant DNA	pRNA-NG-Cnn-S567A	Generated in this study	Table EV4
Recombinant DNA	pRNA-NG-Cnn-S571A	Generated in this study	Table EV4
Recombinant DNA	pRNA-NG-Cnn-2D	Generated in this study	Table EV4
Recombinant DNA	NG-pETM14-Dm_Cnn 377–608	Generated in this study	Table EV4
Recombinant DNA	NG-pETM14-Dm_Cnn 403–608	Generated in this study	Table EV4
Recombinant DNA	NG-pETM14-Dm_Cnn 462–608	Generated in this study	Table EV4
Recombinant DNA	NG-pETM14-Dm_Cnn 490–608	Generated in this study	Table EV4
Recombinant DNA	pETM44-Dm_Cnn 377–608	Generated in this study	Table EV4
Recombinant DNA	pETM44-Dm_Cnn 462–608	Generated in this study	Table EV4
Recombinant DNA	pETM44-Dm_Cnn 490–608	Generated in this study	Table EV4
Recombinant DNA	pETM44-Dm _Cnn 490–579	Generated in this study	Table EV4
Recombinant DNA	pETM44-Dm _Cnn 490–579-2 A	Generated in this study	Table EV4
Recombinant DNA	NG-pETM14-Hs_CDK5RAP2- PReM 535–639	Generated in this study	Table EV4
Recombinant DNA	pETM44-Hs_CDK5RAP2-CM2 1812–1893	Generated in this study	Table EV4
Recombinant DNA	pETM44- Hs_CDK5RAP2 T1863E CM2 1812–1893	Generated in this study	Table EV4
Recombinant DNA	pGEX-6P-1-Ce_SPD5- CM2 1061–1198	Generated in this study	Table EV4
Recombinant DNA	NG-pETM14-SPD5-PReM 541–677	Generated in this study	Table EV4
Recombinant DNA	pETM44-Dm_Cnn-CM2 1082-1148	Generated in this study	Table EV4
Recombinant DNA	pOEM1-MBP-long-linker_Cnn-WT-mGFP-long-linker-His10 (TH2036-pOCC502)	Generated in this study	Table EV4
Recombinant DNA	pOEM1-MBP-long-linker_Cnn-delPReM-mGFP-long-linker-His10 (TH2253-pOCC502)	Generated in this study	Table EV4
Recombinant DNA	pOEM1-MBP-linker_Cnn-delCM2-mGFP-linker-His6 (TH1592-pOCC120)	Generated in this study	Table EV4
Recombinant DNA	pOEM1-MBP-linker_SPD-5-WT-mGFP-linker-His6 (TH0860-pOCC27)	Woodruff et al, 2015	Table EV4
Recombinant DNA	pOEM1-MBP-linker_SPD-5-delPReM (541–677)-mGFP-linker-His6 (TH2475-pOCC27)	Generated in this study	Table EV4
Recombinant DNA	pOEM1-MBP-linker_SPD-5-delCM2L(1161-1198)-mGFP-linker-His6 (TH2077-pOCC27)	Generated in this study	Table EV4
Recombinant DNA	pOEM1-worm_PLK-1-mGFP-linker-His6 (TH1091-pOCC8)	Woodruff et al, 2015	Table EV4
Recombinant DNA	pOEM1-MBP-long-linker_Dm_polo-T182D(CA)-mGFP-long-linker-His10 (TH2194-pOCC502)	Generated in this study	Table EV4
Recombinant DNA	pOEM1-MBP--linker_Dm_polo-WT-mcherry-linker-His6 (TH1547-pOCC216)	Generated in this study	Table EV4
Recombinant DNA	pOEM1-MBP--linker_Dm_polo-T182D(CA)-untagged-linker-His6 (TH1553-pOCC-127)	Generated in this study	Table EV4
Recombinant DNA	pRNA-mNeonGreen (pRNA-NG) Gateway destination vector	Thermo Fisher Scientific/Gateway LR recombination	
Recombinant DNA	pUbq-NG-Cnn	Wong et al, 2022	
Recombinant DNA	pUbq-NG-Cnn-2A	Generated in this study	
Recombinant DNA	pUbq-NG-Cnn-2E	Generated in this study	
Recombinant DNA	Full-length Drosophila Cnn baculovirus expression plasmid	Synthesised by GenScript Biotech, Twist Bioscience, or IDT; subcloned into pOEM1-based vectors	
Recombinant DNA	Human CDK5RAP2 baculovirus expression plasmid	Synthesised by GenScript Biotech, Twist Bioscience, or IDT; subcloned into pOEM1-based vectors	
Recombinant DNA	*C. elegans* SPD-5 baculovirus expression plasmid	Synthesised by GenScript Biotech, Twist Bioscience, or IDT; subcloned into pOEM1-based vectors	
Oligonucleotides and other sequence-based reagents	s1	Generated in this study	Table EV3
Oligonucleotides and other sequence-based reagents	s2	Generated in this study	Table EV3
Oligonucleotides and other sequence-based reagents	s3	Generated in this study	Table EV3
Oligonucleotides and other sequence-based reagents	s4	Generated in this study	Table EV3
Oligonucleotides and other sequence-based reagents	s5	Generated in this study	Table EV3
Oligonucleotides and other sequence-based reagents	s6	Generated in this study	Table EV3
Oligonucleotides and other sequence-based reagents	s7	Generated in this study	Table EV3
Oligonucleotides and other sequence-based reagents	s8	Generated in this study	Table EV3
Oligonucleotides and other sequence-based reagents	s9	Generated in this study	Table EV3
Oligonucleotides and other sequence-based reagents	s10	Generated in this study	Table EV3
Oligonucleotides and other sequence-based reagents	s11	Generated in this study	Table EV3
Oligonucleotides and other sequence-based reagents	s12	Generated in this study	Table EV3
Oligonucleotides and other sequence-based reagents	s13	Generated in this study	Table EV3
Oligonucleotides and other sequence-based reagents	s14	Generated in this study	Table EV3
Oligonucleotides and other sequence-based reagents	s15	Generated in this study	Table EV3
Oligonucleotides and other sequence-based reagents	s16	Generated in this study	Table EV3
Oligonucleotides and other sequence-based reagents	s17	Generated in this study	Table EV3
Oligonucleotides and other sequence-based reagents	s18	Generated in this study	Table EV3
Oligonucleotides and other sequence-based reagents	s19	Generated in this study	Table EV3
Oligonucleotides and other sequence-based reagents	s20	Generated in this study	Table EV3
Oligonucleotides and other sequence-based reagents	s21	Generated in this study	Table EV3
Oligonucleotides and other sequence-based reagents	s22	Generated in this study	Table EV3
Oligonucleotides and other sequence-based reagents	s23	Generated in this study	Table EV3
Oligonucleotides and other sequence-based reagents	s24	Generated in this study	Table EV3
Oligonucleotides and other sequence-based reagents	s25	Generated in this study	Table EV3
Oligonucleotides and other sequence-based reagents	s26	Generated in this study	Table EV3
Oligonucleotides and other sequence-based reagents	s27	Generated in this study	Table EV3
Oligonucleotides and other sequence-based reagents	s28	Generated in this study	Table EV3
Oligonucleotides and other sequence-based reagents	s29	Generated in this study	Table EV3
Oligonucleotides and other sequence-based reagents	s30	Generated in this study	Table EV3
Oligonucleotides and other sequence-based reagents	s31	Generated in this study	Table EV3
Oligonucleotides and other sequence-based reagents	s32	Generated in this study	Table EV3
Oligonucleotides and other sequence-based reagents	s33	Generated in this study	Table EV3
Oligonucleotides and other sequence-based reagents	s34	Generated in this study	Table EV3
Oligonucleotides and other sequence-based reagents	s35	Generated in this study	Table EV3
Oligonucleotides and other sequence-based reagents	s36	Generated in this study	Table EV3
Oligonucleotides and other sequence-based reagents	s37	Generated in this study	Table EV3
Oligonucleotides and other sequence-based reagents	s38	Generated in this study	Table EV3
Oligonucleotides and other sequence-based reagents	s39	Generated in this study	Table EV3
Oligonucleotides and other sequence-based reagents	s40	Generated in this study	Table EV3
Oligonucleotides and other sequence-based reagents	s41	Generated in this study	Table EV3
Oligonucleotides and other sequence-based reagents	s42	Generated in this study	Table EV3
Oligonucleotides and other sequence-based reagents	s43	Generated in this study	Table EV3
Oligonucleotides and other sequence-based reagents	s44	Generated in this study	Table EV3
Oligonucleotides and other sequence-based reagents	s45	Generated in this study	Table EV3
Oligonucleotides and other sequence-based reagents	s46	Generated in this study	Table EV3
Oligonucleotides and other sequence-based reagents	s47	Generated in this study	Table EV3
Oligonucleotides and other sequence-based reagents	s48	Generated in this study	Table EV3
Oligonucleotides and other sequence-based reagents	s49	Generated in this study	Table EV3
Oligonucleotides and other sequence-based reagents	s50	Generated in this study	Table EV3
Oligonucleotides and other sequence-based reagents	s51	Generated in this study	Table EV3
Chemicals, Enzymes and other reagents	Gateway LR recombination system	Thermo Fisher Scientific	
Chemicals, Enzymes and other reagents	KLD Enzyme Mix	NEB	M0554S
Chemicals, Enzymes and other reagents	NEBuilder HiFi DNA Assembly	NEB	
Chemicals, Enzymes and other reagents	Q5 site-directed mutagenesis reagents	NEB	
Chemicals, Enzymes and other reagents	AscI restriction enzyme	NEB	R0558S
Chemicals, Enzymes and other reagents	PCR Purification Kit	QIAGEN	
Chemicals, Enzymes and other reagents	T3 mMESSAGE mMACHINE kit	Thermo Fisher Scientific	AM1348
Chemicals, Enzymes and other reagents	RNeasy MinElute Cleanup Kit	Qiagen	74204
Chemicals, Enzymes and other reagents	Drosophila culture medium	Prepared in-house	
Chemicals, Enzymes and other reagents	Embryo collection plate medium	Prepared in-house	
Chemicals, Enzymes and other reagents	Yeast paste/yeast suspension	Prepared in-house	
Chemicals, Enzymes and other reagents	Voltalef oil	Arkema	H10S PCTFE
Chemicals, Enzymes and other reagents	Ultrafree 0.22 µm PVDF filters	Merck Millipore	
Chemicals, Enzymes and other reagents	Revvity PhenoPlate/ CellCarrier Ultra 384-well ULA-coated microplates	Revvity	
Chemicals, Enzymes and other reagents	FluoSpheres Carboxylate-Modified microspheres, 0.1 µm	Invitrogen	F8801
Chemicals, Enzymes and other reagents	LB broth	Commercial	
Chemicals, Enzymes and other reagents	IPTG	Commercial	
Chemicals, Enzymes and other reagents	HisTrap HP	Cytiva	
Chemicals, Enzymes and other reagents	MBP affinity chromatography resin/columns	Cytiva / NEB	
Chemicals, Enzymes and other reagents	GSTrap	Cytiva	
Chemicals, Enzymes and other reagents	Benzonase	Commercial	
Chemicals, Enzymes and other reagents	Protease inhibitor cocktail tablet, EDTA-free	Commercial	
Chemicals, Enzymes and other reagents	Protino Ni-NTA	Macherey-Nagel	
Chemicals, Enzymes and other reagents	MBP-TRAP columns	Cytiva HP	
Chemicals, Enzymes and other reagents	Amicon 100 kDa filters	Cytiva	
Chemicals, Enzymes and other reagents	Maltose	Commercial	
Chemicals, Enzymes and other reagents	PEG 20 kDa	Commercial	
Chemicals, Enzymes and other reagents	HEPES	Commercial	
Chemicals, Enzymes and other reagents	Tris-HCl/Tris	Commercial	
Chemicals, Enzymes and other reagents	NaCl	Commercial	
Chemicals, Enzymes and other reagents	KCl	Commercial	
Chemicals, Enzymes and other reagents	DTT	Commercial	
Chemicals, Enzymes and other reagents	TCEP	Commercial	
Chemicals, Enzymes and other reagents	ATP	Commercial	
Chemicals, Enzymes and other reagents	MgCl_2_	Commercial	
Chemicals, Enzymes and other reagents	ZnCl_2_	Commercial	
Chemicals, Enzymes and other reagents	Imidazole	Commercial	
Chemicals, Enzymes and other reagents	CHAPS	Commercial	
Chemicals, Enzymes and other reagents	Glycerol	Commercial	
Chemicals, Enzymes and other reagents	BSA standard	Commercial	
Chemicals, Enzymes and other reagents	Human IgG standard	Commercial	
Chemicals, Enzymes and other reagents	Silicone gaskets	Grace BioLabs	103250
Chemicals, Enzymes and other reagents	Isopropanol	Commercial	
Chemicals, Enzymes and other reagents	Milli-Q water	Commercial	
Chemicals, Enzymes and other reagents	Uranyl acetate	Commercial	2% (w/v)
Chemicals, Enzymes and other reagents	Polyethylene glycol (20 kDa)	Commercial	
Chemicals, Enzymes and other reagents	Maltose-containing buffer	In-house prepared	
Chemicals, Enzymes and other reagents	Bsu36I	Commercial	
Chemicals, Enzymes and other reagents	PspOMI	Commercial	
Chemicals, Enzymes and other reagents	Artificial SpeI site generated by silent mutagenesis	Generated in this study	
Chemicals, Enzymes and other reagents	GST-3C protease	Lab purified	
Chemicals, Enzymes and other reagents	His-3C protease	Lab purified	
Chemicals, Enzymes and other reagents	*C. elegans* GFP-PLK1	Purified protein	
Chemicals, Enzymes and other reagents	NG-SPD5-PReM / SPD5 1061–1198	Purified protein	
Chemicals, Enzymes and other reagents	CDK5RAP2 PReM	Purified protein	
Chemicals, Enzymes and other reagents	CDK5RAP2 CM2	Purified protein	
Chemicals, Enzymes and other reagents	SPD-5 CM2	Purified protein	
Chemicals, Enzymes and other reagents	Cnn CM2	Purified protein	
Chemicals, Enzymes and other reagents	Cnn fragments 403–608/377–608/462–608/490–608	Purified proteins	
Chemicals, Enzymes and other reagents	Superdex 200 Increase 10/300 GL column	Cytiva	
Chemicals, Enzymes and other reagents	Superdex 75 10/300 Increase column	Cytiva	
Chemicals, Enzymes and other reagents	Superose 6 Increase 10/300 column	Cytiva	
Chemicals, Enzymes and other reagents	HiLoad 16/600 Superose 6 column	Cytiva	
Software	Volocity	PerkinElmer	
Software	NIS Elements	Nikon	v5.42.03
Software	cellSens	Olympus	
Software	Andor iQ	Andor	v3.6
Software	TrackPy	Allan et al, 2016	
Software	Fiji	Open source	
Software	StackReg plugin	Fiji	
Software	MATLAB	MathWorks	2023b or later
Software	AlphaFold3	Abramson et al, 2024	
Software	UCSF ChimeraX	Meng et al, 2023	v1.9
Software	MUSCLE	Open source	
Software	Clustal Omega	Open source	
Software	Jalview	v2.11.4.1	
Software	AcquireMP	Refeyn	v2.5.0
Software	DiscoverMP	Refeyn	v2.5.0
Software	CCP4 suite	CCP4	
Software	Phenix	Adams et al, 2010	
Software	Coot	Emsley and Cowtan, 2004	
Software	Xia2 dials	DLS	
Software	Xia2 3dii	DLS	
Software	Molrep	CCP4	
Software	Phaser	Phenix	
Software	Refmac	CCP4	
Software	Buccaneer	CCP4	
Software	Astra	Wyatt Technologies	v7
Software	GraphPad Prism	GraphPad	v10.4.2
Software	GROMACS	2022.4	
Software	CHARMM-GUI Martini Solution Maker	Jo et al, 2008; Qi et al, 2015	
Other	PerkinElmer ERS spinning disk confocal system	PerkinElmer	
Other	Zeiss Axiovet 200 M microscope	Carl Zeiss	
Other	63x / 1.4 NA oil objective	Carl Zeiss	
Other	ImmersolT 518 F immersion oil	Carl Zeiss	
Other	Orca ER CCD camera	Hamamatsu Photonics	
Other	405, 488, 561, and 642 nm solid-state lasers	Oxxius S.A.	
Other	Nikon Eclipse Ti motorised stand	Nikon	
Other	Yokogawa CSU-X1 spinning disk scan head	Yokogawa	
Other	Nikon Plan Apo 100×/1.45 NA oil objective	Nikon	
Other	Andor iXON 897 EMCCD camera	Andor	
Other	Olympus IXplore SpinSR microscope	Olympus	
Other	Yokogawa CSU-W1 spinning disk	Yokogawa	
Other	SoRa module	Olympus	
Other	U ApoN O-TIRF 100×/1.49 NA oil objective	Olympus	
Other	ORCA-Fusion BT sCMOS camera	Hamamatsu	
Other	Zeiss 880 microscope	Carl Zeiss	
Other	Airyscan detector	Carl Zeiss	
Other	20×/0.8 NA Plan-Apochromat lens	Carl Zeiss	
Other	Andor spinning-disk FRAPPA system	Andor	
Other	Olympus IX81 inverted motorised microscope	Olympus	
Other	UPlanSApo 60×/1.2 NA water-immersion objective	Olympus	
Other	Refeyn TwoMP instrument	Refeyn	Oxford, UK
Other	24 × 50 mm coverslips	High-precision coverslips	
Other	Mosquito LCP	ttplabtech	
Other	Diamond Light Source beamline i04	DLS	
Other	Diamond Light Source beamline i03	DLS	
Other	LM-20 microfluidizer	Microfluidics	
Other	XPN-90 ultracentrifuge	Beckman Coulter	
Other	Ti45 rotor	Beckman Coulter	
Other	0.22 µm PES filter	Commercial	
Other	JEOL Flash 120 kV TEM	JEOL	
Other	Gatan Rio camera	Gatan	
Other	DAWN HELEOS-II 18-angle light scattering detector	Wyatt Technologies	
Other	U9-M UV/Vis detector	Cytiva	
Other	Optilab T-rEX refractive index monitor	Wyatt Technologies	
Other	AktaPure 25 system	Cytiva	
Other	C267 TEM grids	TAAB	
Other	BioImage Archive	Hartley et al, 2022	
Other	ProteomeXchange	ProteomeXchange Consortium	
Other	Advanced Proteomics Facility, Department of Biochemistry, University of Oxford	University of Oxford	
Other	Dunn School Bioimaging facility	University of Oxford	
Other	Protein Expression and Purification (PEPC) facility	MPI-CBG	
Other	Light Microscopy facility (LMF)	MPI-CBG	
Antibodies	Rabbit anti-Cnn	Lucas and Raff, 2007	
Antibodies	HRP-conjugated donkey anti-rabbit IgG	Cytiva Life Sciences; NA934V (lot 17876631)	

### *Drosophila melanogaster* stocks and husbandry

The *Drosophila* stocks used, generated and/or tested in this study are listed in Table EV1. The precise stocks used in each experiment (and the relevant Fig) are listed in Table EV2. Flies were maintained on *Drosophila* culture medium (0.68% agar, 2.5% yeast extract, 6.25% cornmeal, 3.75% molasses, 0.42% propionic acid, 0.14% tegosept, and 0.7% ethanol) in 8 cm × 2.5 cm plastic vials or 0.25-pint plastic bottles. For microscopy and immunoblot experiments, flies were placed in embryo collection cages on fruit juice plates (see below) with a drop of yeast paste. Fly handling was performed as previously described (Roberts, 1998).

### Transgenic fly line generation

Transgenic fly lines were generated via random P-element insertion (injected, mapped, and balanced by ‘The University of Cambridge Department of Genetics Fly Facility’). For transgene selection, the *w*^*+*^ gene marker was included in the transformation vectors, and constructs were injected into the *w*^*1118*^ genetic background.

### Molecular biology

All primer and plasmid sequences are listed in Tables EV3 and  EV4. Full-length *Drosophila* Cnn was cloned into the pRNA-mNeonGreen (pRNA-NG) Gateway destination vector by LR recombination (Thermo Fisher Scientific) to generate pRNA-NG-Cnn (p1). Truncation mutants were derived from p1 by PCR linearisation with the indicated primer pairs, followed by re-circularisation using the KLD Enzyme Mix (NEB, M0554S). The following deletion constructs were generated: Δ376–609 (p2), Δ376–402 (p3), Δ376–462 (p4), Δ376–490 (p5), Δ551–609 (p6), and Δ583–609 (p7). A CM2 deletion mutant (ΔCM2; p8) was generated by linearising p1 with primers s8/s9. For domain-replacement constructs, the fly CM2 region was replaced with either the human CDK5RAP2 CM2 or the CM2-like region of *C. elegans* SPD-5. These inserts were amplified using primers s10/s11 (CDK5RAP2) or s12/s13 (SPD-5) and assembled into the s8/s9-linearised backbone using NEBuilder HiFi DNA Assembly (NEB) to generate plasmids p9 (fly–human CM2 chimaera) and p10 (fly–worm CM2 chimaera).

Non-phosphorylatable (S567A/S571A; p11) and phosphomimetic (S567E/S571E; p12) mutants were generated in pRNA-NG-Cnn (p1) and pUbq-NG-Cnn by Q5 site-directed mutagenesis (NEB) using primer pairs s14/s15 and s16/s15, respectively. Single-site mutants (S567A or S571A) and additional phosphomimetic variants (e.g., S567D/S571D) were generated by analogous PCR-based mutagenesis of p1 using the primer pairs listed in Table EV3. All constructs were verified by Sanger sequencing across the full coding region prior to downstream applications.

For bacterial expression, *Drosophila* Cnn fragments (residues 403–608, 377–608, 462–608 [WT or S567A/S571A], and 490–608) were cloned into a modified pETM14 vector encoding an N-terminal His₆–mNeonGreen tag. Equivalent constructs were generated for human CDK5RAP2 (residues 535–639) and *C. elegans* SPD-5 (residues 541–677) using the same backbone. For MBP-tagged constructs, Cnn fragments (403–608, 377–608, 462–608, 490–608 and 490–579 [WT or S567A/S571A]) were cloned into NcoI-digested pETM44 to generate N-terminal His₆–MBP fusions, and the same strategy was used for CDK5RAP2 CM2 (residues 1812–1893) and CDK5RAP2 CM2 T1863E mutant. For GST-tagged constructs, *C. elegans* SPD-5 (residues 1061–1198) was cloned into BamHI-digested pGEX-6P-1 to generate an N-terminal His₆–GST fusion.

Full-length *Drosophila* Cnn, human CDK5RAP2, and *C. elegans* SPD-5 were synthesised (GenScript Biotech, Twist Bioscience, or Integrated DNA Technologies) and subcloned into pOEM1-based baculovirus expression plasmids using NotI and AscI restriction sites. All cloning steps were performed using standard molecular biology procedures.

### mRNA synthesis

For in vitro transcription, the constructs were digested and linearised by AscI (R0558S, NEB), and purified with the PCR Purification Kit (QIAGEN). Around 1.6–3.2 μg of digested and purified DNA was used to synthesise mRNA with the T3 mMESSAGE mMACHINE kit (AM1348, ThermoFisher Scientific). The mRNA product was purified with the RNeasy MinElute Cleanup Kit (74204, Qiagen). All RNAs were stored at -70 °C. The final concentrations of RNA are used at 2 μg/μL.

### Embryo collection and injection

Embryos were collected from plates (40% cranberry-raspberry juice, 2% sucrose, and 1.8% agar) supplemented with fresh yeast suspension. For live-imaging experiments, embryos were collected for 1 h at 25 °C, and aged at 25 °C for 45–60 min. Embryos were dechorionated by hand, mounted on a strip of glue on either a 35-mm glass-bottom Petri dish with 14 mm micro-well (MatTek) and desiccated for 1 min at 25 °C before covering with Voltalef oil (H10S PCTFE, Arkema) to avoid further desiccation.

### Immunoblotting

Protein expression level was estimated as described previously (Aydogan et al, 2018). Rabbit anti-Cnn was used as a primary antibody (Lucas and Raff, 2007). HRP-conjugated donkey anti-rabbit (NA934V lot:17876631, Cytiva Lifescience) secondary antibodies were used.

### In vitro condensate formation assay

Condensates of Cnn-WT, SPD-5-WT, and the corresponding deletion mutants were generated by diluting concentrated proteins, stored in high-salt buffer [50 mM HEPES pH 7.25, 500 mM KCl, 5% (v/v) glycerol, 1 mM DTT], into assay buffer of physiological ionic strength [50 mM HEPES pH 7.25, 150 mM KCl, 1 mM ATP/Mg²⁺, 2 mM TCEP] supplemented with 4% (v/v) polyethylene glycol (20 kDa). Prior to use, proteins were clarified by filtration through Ultrafree 0.22 µm PVDF filters (Merck Millipore) to remove residual aggregates. For imaging experiments, assay buffer was dispensed into 384-well ULA-coated microplates (Revvity PhenoPlate™, formerly CellCarrier Ultra), followed by the addition of the respective protein solutions to induce condensate formation.

### In vitro phosphorylation

Purified NG-SPD5-PReM or SPD5_1061-1198_ was phosphorylated using *C. elegans* GFP-PLK1 in a time-course assay. Reactions (400 µl total volume) contained 100 µM purified protein, 10 µl GFP-PLK1, 2 mM ATP, and 1× kinase buffer (from a 10× stock). Protein, PLK1, and ATP stocks were thawed on ice prior to use.

Reactions were incubated at 23 °C (optimal for *C. elegans* PLK1 activity) and sampled at *T *= 0, 1, 2, 3, and 4 h. At each time point, 10 µl was withdrawn for analysis by 20% SDS-PAGE, and ATP was replenished. Following the time course, samples were further purified on a pre-equilibrated Superdex 200 Increase 10/300 GL column (Cytiva) in buffer containing 20 mM Tris-HCl (pH 7.5), 150 mM NaCl, and 0.5 mM TCEP. Phosphorylation was confirmed by mass spectrometry (Advanced Proteomics Facility, Department of Biochemistry, University of Oxford).

### Live imaging and microscopy

Live embryo imaging was performed at 23 °C using a PerkinElmer ERS spinning disk confocal system mounted on a Zeiss Axiovet 200 M microscope and controlled with Volocity software (PerkinElmer). Images were acquired with a 63×/1.4 NA oil objective (Carl Zeiss) using ImmersolT 518 F immersion oil (RI = 1.518) to minimise spherical aberration. Fluorescence was detected with a 15-bit Orca ER CCD camera (Hamamatsu Photonics) operated at a gain of 200 V. Excitation was provided by 405, 488, 561, and 642 nm solid-state lasers (Oxxius S.A.). For dual-colour acquisitions, red and green channels were imaged sequentially in each z-slice using the UltraVIEW ERS “Emission Discrimination” setting with 520 nm long-pass (green) and 620 nm long-pass (red) emission filters. Z-stacks were collected at 0.5 µm intervals, with imaging parameters (section number, time step, laser power, exposure) optimised for each experiment.

For estimation of protein saturation concentrations (C_sat_, Fig. 8D), imaging was performed at room temperature on a Nikon Eclipse Ti motorised stand equipped with a Yokogawa CSU-X1 spinning disk scan head and a Nikon Plan Apo 100×/1.45 NA oil objective. Samples were imaged with immersion oil (RI = 1.518) and illuminated using a 488 nm diode-pumped solid-state laser (Omicron) at 2% power. Emission was collected through a 525/30 band-pass filter. Images were acquired on an Andor iXON 897 EMCCD camera (16 µm pixel size) at 16-bit depth with no binning and a fixed amplifier gain of 200 V. Acquisition was controlled with NIS Elements software (v5.42.03), and imaging parameters were held constant across conditions for quantification.

Condensate diameters were measured using an Olympus IXplore SpinSR microscope equipped with a Yokogawa CSU-W1 spinning disk and SoRa (Super-resolution via Optical Re-assignment) module. Samples were excited with a 488 nm diode-pumped solid-state laser at 50% intensity, and emission was collected with a 525/50 nm band-pass filter. Imaging was performed using a ×3.2 SoRa magnification lens combined with an Olympus U ApoN O-TIRF 100×/1.49 NA oil objective, yielding a final system magnification of ×320. Fluorescence was recorded with a Hamamatsu ORCA-Fusion BT sCMOS camera (16-bit depth, 23.0 MHz pixel clock). For each condition, 20–22 randomly selected single-plane images were acquired per well. Image acquisition was managed with Olympus cellSens software.

For the experiment shown in Fig. 2, purified NG-Cnn fragments (residues 403–608, 377–608, 462–608, or 490–608) were mixed with purified Cnn CM2 at a 1:10 molar ratio in a total reaction volume of 30 µl. In Fig. 4, purified NG-Cnn 462–608 (WT or S567A/S571A mutant) was mixed with purified Cnn CM2 at the same ratio. As for Fig. 5, NG-Cnn 462–608 was mixed with either Cnn CM2, CDK5RAP2 CM2, CDK5RAP2 T1863E CM2 or SPD5 CM2. In Fig. 6, CDK5RAP2 PReM (residues 535–639) was mixed with purified CDK5RAP2 CM2 (residues 1812–1893) or its CDK5RAP2 T1863E CM2 mutant. NG-SPD5 541–677 (WT or in vitro PLK1-phosphorylated) was mixed with either non-phosphorylated or phosphorylated SPD5 CM2 for Fig. 7.

In all reactions, red beads (FluoSpheres Carboxylate-Modified microspheres, 0.1 µm; Invitrogen F8801) were diluted 1:1000 in buffer and added to the reactions at a final dilution of 1:10,000. The reaction buffer contained 20 mM Tris-HCl (pH 7.5, room temperature), 150 mM NaCl, 0.5 mM TCEP, and 10 µM ZnCl₂. Reactions were incubated at room temperature for 30 min, after which 8 µl was applied to a slide (pipette tip cut to minimise shear) and immediately covered with an 18 × 18 mm coverslip. Networks were visualised in detail using a Zeiss 880 microscope fitted with an Airyscan detector using a 63×1.4NA lens. The resulting Z-stacks were Airyscan-processed in 3D with a strength value of Auto (~ 6) and visualised using a maximum intensity projection.

### In vitro half-compartment FRAP (fluorescence recovery after photobleaching) experiments

FRAP experiments on in vitro condensates were carried out using an Andor spinning-disk FRAPPA system mounted on an Olympus IX81 inverted motorised microscope equipped with a Yokogawa CSU-X1 scan head. Condensates were prepared from 3 µM protein (as in the droplet formation assay) and left to settle for 15 min before bleaching. Photobleaching was performed with a 488 nm laser line (via AOTF) at 15% power using the integrated FRAPPA module (30 ms dwell time, 2 repeats), targeting ~50% of each condensate after acquiring 3–5 pre-bleach frames. Images were collected with an Olympus UPlanSApo 60×/1.2 NA water-immersion objective and recorded on an Andor iXon 897 EMCCD camera (SN 3880) at 30 ms exposure with EM gain set to 200 V. All acquisition and bleaching steps were controlled by Andor iQ software (v3.6).

### Image processing and analysis

For centrosome analysis, raw images were maximum-intensity projected, and background was corrected for uneven illumination using a custom Python script. Centrosomes were identified using the Crocker–Grier centroid-finding algorithm (Crocker and Grier, 1996) implemented in TrackPy (Allan et al, 2016). Signal-to-background thresholds were determined using Otsu’s method (Otsu, 1979). Centrosome area and integrated intensity were then calculated from the segmented regions.

For condensate quantification (Fig. 8), single-plane fluorescence images were converted to grayscale, denoised with a Gaussian filter, and segmented by Otsu thresholding. Binary masks were generated, and small objects below a defined size cutoff were excluded. Masks were overlaid on the original images for quality control. Quantitative features—including condensate number, size distribution, mean fluorescence intensity inside versus outside condensates, and condensate fraction—were extracted and exported in CSV format for downstream analysis. Additional processing was performed in Fiji. Custom Python scripts are available on request.

For network quantification (Figs. 2A–D, 4B, 5B and 7C), five grids of 100 z-stacks per coverslip were acquired using a 20×/0.8 NA Plan-Apochromat lens, with three coverslips analysed per construct. Stacks were maximum-intensity projected and thresholded above background using a bespoke Python script, and the number of pixels above threshold was quantified per image.

Fluorescence recovery analysis followed the procedure described previously (Hubatsch et al, 2021) using custom software validated in Matlab 2023b or later. Briefly, droplet positions were registered in Fiji with the StackReg plugin, and recovery dynamics were extracted by drawing a three-pixel-wide stripe ROI perpendicular to the bleach boundary at each time point. The stripe was collapsed into 1D by averaging across its width and then used as the initial condition for FRAP analysis. A one-dimensional diffusion equation with experimentally determined boundary conditions was fit globally to the recovery curves of each droplet to obtain diffusion coefficients. To account for proteins that were only transiently rather than permanently bleached (Sinnecker et al, 2005), a uniform exponential recovery term was included in the model.

### Recombinant protein expression and purification

Recombinant proteins were expressed in *Escherichia coli* BL21 (DE3) cells cultured in LB broth. Protein expression was induced with 0.8-1 mM IPTG after cells reached an O.D of ~0.6-0.7. Cells were left expressing overnight at 18 °C before being harvested. Cells were lysed in a buffer containing 20 mM TRIS, 500 mM NaCl, 0.5 mM TCEP using an Emulsiflex C5 homogeniser (Avestin) at 15,000 psi and at 4 °C. The soluble protein fraction was then separated from the insoluble fraction by centrifugation at 5000 rpm for 30 min. Proteins of interest were then purified using Ni-NTA affinity chromatography (HisTrap HP; Cytiva), MBP affinity chromatography, GSTrap (Cytiva), depending on the tag, followed by size-exclusion chromatography in buffer containing 20 mM Tris (pH 7.5), 150 mM NaCl, and supplied with 0.5 mM TCEP. The protein concentration was measured using a NanoDrop Spectrophotometer ND-1000 (ThermoFisher).

For SEC-MALS analysis and crystallisation trials or further use, the N-terminal tag was cleaved off using a lab-purified GST-3C protease. The resulting untagged protein was further purified via reverse Ni-NTA chromatography and a second round of size-exclusion chromatography in the same SE buffer.

### Full-length protein purification and condensate assays

The baculoviruses for protein expression in Sf9 cells were generated using the FlexiBAC system (Lemaitre et al, 2019), and subsequently 1%(vol/vol) of the respective P2 stocks of these baculoviruses were used to infect Sf + /Sf9 cultures for 72 h at 28 °C. The cells were harvested and resuspended in lysis buffer (50 mM HEPES pH 7.25, 500 mM KCl, 20 mM imidazole, 5% Glycerol, 0.1% CHAPS, 1 mM MgCl_2_, 1 mM DTT, 30 µL Benzonase/100 mL Buffer, 1× protease inhibitor cocktail tablet EDTA-free/ 50 mL buffer), either flash-frozen in liquid N_2_, and stored at −80 °C till further purification, or directly used for purification. The cells were subjected to lysis on a LM-20 microfluidizer, with 2 passes on ice at 11,000 p.s.i. The lysates were centrifuged in a Ti45 rotor in an XPN-90 (Beckman Coulter) ultracentrifuge, at 29,000 rpm for 45 min at 4 °C. The supernatants were carefully decanted and filtered on a 0.22 µm PES filter and allowed to bind to 5 mL Protino Ni-NTA (Macherey-Nagel) columns, pre-equilibrated with the lysis buffer, at a rate of 2 ml/min. The columns were washed with 15 column volumes (CV) of lysis buffer, 5 CVs of 1 M KCl containing lysis buffer, and then proteins were eluted using 250 mM imidazole. The eluted solutions were then purified over either amylose resin (NEB) or 5 mL MBP-TRAP columns (Cytiva HP), pre-equilibrated with MBP-binding buffer (50 mM HEPES pH 7.25, 500 mM KCl, 5% Glycerol, 0.1% CHAPS, 1 mM DTT), followed by 5 CVs wash with the same buffer. Then 15 mM maltose-containing buffer was used to elute the proteins from the MBP-TRAP columns. The obtained protein solutions were concentrated on 100 kDa amicon filters (Cytiva) and the N-terminal MBP and C-terminal His tags were cleaved off, using an in-house purified His-3C protease, before being subjected to a final size-exclusion step, either on a 24 mL Cytiva superose 6 increase 10/300 column or a 120 mL Cytiva HiLoad 16/600 Superose 6 column, pre-equilibrated with storage buffer (50 mM HEPES pH 7.25, 500 mM KCl, 5% Glycerol, 1 mM DTT). Peak fractions containing the proteins were pooled and further concentrated on 100 kDa Amicon filters. The protein concentrations were determined at 280 nm and flash-frozen for storage at −80 °C.

After a series of trial experiments, 20 kDa PEG at 4% (w/vol) was chosen to compare the properties of the Cnn and SPD-5 condensates, as this supported the most robust formation of the various WT and mutant condensates. While lower molecular weight PEGs have been commonly used, recent work showed that smaller crowders exhibit partitioning into the dense condensed phase, resulting in a combination of both homotypic interactions between the protein molecules as well as heterotypic interactions between the crowder and protein molecules (Chauhan et al, 2024). In the case of higher molecular weight PEGs, the partition coefficients of the crowders were found to be <1, making them more effective depletants, aiding condensation, without contributing directly to the properties of the condensates. The use of 20 kDa PEG was therefore intended to minimise such crowder contributions, though we note that these partitioning behaviours have been characterised only in different model condensate systems.

### Mass spectroscopy

All Mass Spectroscopy was performed by the Advanced Proteomics Facility at the Department of Biochemistry, University of Oxford.

#### Sample preparation

Protein samples were diluted in 4 M urea and 0.1 M ammonium bicarbonate, reduced with 10 mM tris(2-carboxyethyl)phosphine (TCEP) for 30 min and alkylated with 50 mM 2-chloroacetamide in the dark for 30 min at room temperature with continuous shaking (750–850 rpm). Proteins were digested sequentially with LysC (1:100, 2 h, 37 °C) followed by trypsin (1:40, 16–18 h, 37 °C) overnight at 37 °C in the presence of 2 mM calcium chloride. Following digestion, samples were acidified to a final concentration of 5% formic acid, desalted using C18 StageTips pre-equilibrated with acetonitrile and 0.1% trifluoroacetic acid (TFA), washed with 0.1% TFA, and peptides were eluted using 50% acetonitrile containing 0.1% TFA. Eluted peptides were dried by vacuum centrifugation prior to LC-MS/MS analysis.

#### LC–MS/MS

Peptide analysis was performed by nano-flow liquid chromatography coupled to tandem mass spectrometry. For Orbitrap Q Exactive analyses, peptides were separated on an Easy-Spray RSLC C18 analytical column following trapping on a PepMap100 C18 precolumn using either an EASY-nLC 1000 or Ultimate 3000 RSLC system. Peptides were resolved using 15–30 min linear gradients of 15–35% acetonitrile containing 0.1% formic acid at a flow rate of 200 nL/min. Data-dependent acquisition (DDA) was performed with full MS scans acquired at 70,000 resolution over an *m/z* range of 350–1500, followed by HCD fragmentation of the 5 or 10 most intense precursor ions using 30% normalised collision energy. MS/MS spectra were acquired at 17,500 resolution with dynamic exclusion of 5–10 s. For Astral Zoom analyses, peptides were separated using a Vanquish Neo UHPLC system equipped with a PepMap Neo C18 trapping column and a 25 cm × 150 μm PepMap RSLC C18 analytical column using a 37 min gradient at 50 °C and 1.0 μL/min. Data-independent acquisition (DIA) was performed over an *m/z* range of 380–980 using 300 isolation windows of 2 Th, Orbitrap resolution of 240,000, AGC target of 5 × 10⁶, and 3 ms maximum injection time for both MS and MS/MS scans. Fragmentation was achieved using higher-energy collisional dissociation (HCD) with 28% normalised collision energy.

#### Data analysis

DDA data were processed using Sequest HT implemented in Proteome Discoverer v1.4 against customised protein databases containing the relevant target protein sequences together with 283 common contaminant proteins. Carbamidomethylation ( + 57,0215) of cysteine was specified as a fixed modification, while methionine oxidation ( + 15,9949), protein N-terminal acetylation ( + 42,0106), and phosphorylation ( + 79,9663) of serine, threonine and tyrosine residues were included as variable modifications. Up to two missed cleavages were permitted, precursor and fragment mass tolerances were set to 20 ppm and 0.6 Da, respectively, and peptide-spectrum matches were filtered to a 1% false discovery rate (FDR). Phosphorylated peptide spectra were manually inspected to confirm site assignments. DIA datasets were analysed using DIA-NN (v2.2), employing an in silico spectral library generated from the Fasta protein database supplemented with the relevant recombinant protein sequences. Search parameters included fixed carbamidomethylation of cysteine, variable oxidation of methionine, protein N-terminal acetylation, and phosphorylation of serine, threonine and tyrosine, allowing up to two missed cleavages. Match-between-runs was enabled, protein quantification was performed using QuantUMS, and identifications were filtered at a 1% FDR using the default DIA-NN settings.

### Mass photometry

Mass photometry experiments were conducted using a Refeyn TwoMP instrument (Refeyn, Oxford, UK) to determine the molecular masses of full-length purified proteins. The technique relies on converting interferometric scattering contrast values into molecular weights using calibration curves generated from known protein standards, including bovine serum albumin (BSA) and human immunoglobulin G (IgG). Briefly, high-precision 24 × 50 mm coverslips were cleaned by alternating between isopropanol and Milli-Q water in a bath sonicator (3 min per wash), then dried under nitrogen. Silicone gaskets (Grace Biolabs, Cat. No. 103250) were cleaned in the same solvents (without sonication) and mounted centrally on the dried coverslips prior to loading onto the microscope stage. Standard stocks of BSA and IgG were diluted to 100 nM in MP dilution buffer (20 mM HEPES, 150 mM KCl, pH 7.25). For each measurement, 18 µL of this buffer was added to the gasket chamber and used to focus the microscope objective. Then, 2 µL of the 100 nM standard solution was added to achieve a final concentration of 10 nM. After gentle mixing, MP measurements were acquired immediately using AcquireMP software (Refeyn, v2.5.0). Contrast histograms were generated and fitted with Gaussian functions using DiscoverMP software (Refeyn, v2.5.0). The peaks corresponding to monomeric BSA (66 kDa), dimeric BSA (132 kDa), and IgG (150 kDa) were used to create a calibration curve for molecular weight determination. The same procedure was applied to the purified full-length protein samples. Molecular weight distributions were fitted with Gaussian curves using the previously generated calibration curve. A minimum of three technical replicates were performed for each protein sample to ensure reproducibility.

### Crystallisation, data collection and processing

#### Cnn 490–579 WT and 2A mutant

Crystallisation was performed using the sitting-drop vapour-diffusion method. Cnn 490–579 WT crystals were seen four days after setting the screening plates at 6 mg/ml, 12 mg/ml or 18 mg/ml concentration in mother condition containing 0.8 m potassium/sodium phosphate (pH 7.0). Tag cleaved Cnn 490–579 2 A mutant purified protein was used to set crystallisation screens at a concentration of 10 mg/ml. Cnn 490–579 2A crystals grew in several conditions with the one giving the best diffraction growing in 1.32 M potassium/sodium phosphate pH 7 in the drop containing 75% protein sample and 25% of the buffer. Pipetting was carried out using mosquito LCP (ttplabtech). Crystal hits were screened by X-ray diffraction, and datasets were collected at beamline I04 for Cnn 490–579 WT protein and I03 for the Cnn 490–579 2A mutant at the Diamond Light Source (DLS; Oxford, UK).

For Cnn 490–579 WT, Xia2 dials data reduction files were used for the initial structure model. As a homology model for phaser, a truncated version of CnnD490-K544(L535E) based on its complex structure with the Cnn C-terminus (PDB code: 5MW9) was used, which only retained the core dimer and lacked the termini (as well as its binding partner in the structure, the Cnn C-terminus). It was hypothesised that the dimeric core might be structurally conserved in CnnD490-N579. Indeed, a single solution with a translation function Z-score of 9.6 was obtained when using this search model. After some minor refinement involving changing a few side chain orientations, the model was fixed, and phaser instructed to search for a straight, 20 amino acids long, polyalanine α-helix (adapted from PDB code: 5AL6). This resulted in 14 solutions, with the top hit having a translational function Z-score of 11.2. This cycle of placing a short stretch of a polyalanine α-helix via phaser or molrep followed by refinement via Coot and refmac was repeated several times until no satisfactory placements were possible anymore. At this point, buccaneer was used with this partial solution and the expected protein sequence in order to build in the correct sequence of amino acids. The obtained model was used for refinement in phenix.refine, including rigid body refinement to account for minor differences in the unit cell dimensions.

MTZ file from Cnn 490–579 2A mutant was processed with Xia2 3dii and was further indexed to space group P6_1_ using CCP4 suite. Structure was solved by molecular replacement using Phenix (Adams et al, 2010) using Cnn 490–579 WT structure (see above). Successful MR solutions were subjected to iterative manual building/refinement using Coot (Emsley and Cowtan, 2004) and Phenix.refine. Data processing and refinement statistics are summarised in Table 1.

**Table 1:** Data collection and refinement statistics of Cnn 490–579 WT and 2A mutant.

Protein	Cnn_490-579_ WT	Cnn_490-579_ 2 A
Beamline	Diamond i04	Diamond i03
Wavelength	0.9795	0.9763
Resolution limit (Å)	107.84 − 2.04 (2.07 − 2.04)	46.45 − 2.00 (2.05 − 2.00)
Space group	P61	P61
Unit cell dimensions	58.98, 58.98, 107.84 90, 90, 120	59.45, 59.45, 107.69 90, 90, 120
Unique reflections	13590 (634)	14638 (1118)
Multiplicity	11.2 (11.4)	19.1 (12.5)
Completeness	100.0 (96.8)	100.0 (100)
I/sigma	8.1 (1.4)	15.5 (1.0)
Rmerge (%)	15.8 (175.4)	8.6 (226.2)
Rmeas (%)	16.6 (183.7)	8.8 (235.8)
Rpim (%)	5.0 (54.3)	2.1 (66.1)
CC1/2	1.0 (0.7)	0.999 (0.499)
Processing pipeline	Xia2 dials (DLS)	Xia2 3dii (DLS)

Crystal	Cnn_490-579_ WT	Cnn_490-579_ 2 A
Resolution (Å)	2.04	2
Rwork/Rfree (%)	20.55/21.82	22.9/27.2
Asymmetric unit	Dimer	Dimer
Observed residues	Chain A: 493–576 Chain B: 492–577	Chain A: 494–577 Chain B: 495–574
% Data completeness (In resolution range)	100	100
Total number of atoms	1452	1333
Average B factors (Å)	59	68
Ramachandran statistics (%): Favoured	100	100
Rotamer outliers (%)	0	2.2
PDB code		9S7L

Statistics from the highest resolution shell are indicated in parentheses.

### Protein structure prediction and visualisation

To identify the PReM region of *H. sapiens* CDK5RAP2, we first screened for potential interactions between the full N-terminus of CDK5RAP2 and its CM2 domain (residues 1812–1893) using AlphaFold3 with default settings (Abramson et al, 2024). Given prior evidence suggesting dimerisation, the input was designed to account for potential protein-protein interfaces. Although the initial prediction yielded a low ipTM score, the output was systematically analysed for plausible interaction interfaces.

Based on this analysis, the N-terminal region was fragmented into smaller domains while preserving its helical structural integrity. The ipTM score served as a preliminary filter for promising candidates. After iterative refinements of the input parameters, the CDK5RAP2 535–639 region was decided on as a potential binding target for the CM2 domain among others, despite its ipTM score remaining below 0.5. UCSF Chimaera X-1.9 (Meng et al, 2023) was used for structural analysis and Fig generation.

For the monomeric and dimeric structural predictions of the different Cnn deletions, CDK5RAP2 CM2 and SPD5 CM2, the AlphaFold3 server was utilised with the default settings applied. The AlphaFold3 server was also used for SPD5 541–677 anti-parallel dimer prediction in Fig. 7. UCSF Chimaera X-1.9 was used for structural analysis and Figure generation.

### Multiple sequence alignment (MSA)

Protein sequences of interest were obtained from UniProt using the following accession numbers: Cnn (*D.* *melanogaster*): UniProt ID P54623; CDK5RAP2 (*H.* *sapiens*): UniProt ID Q96SN8; and Spd5 (*C.* *elegans*): UniProt ID P91349.

MSAs were generated for the PReM region of Cnn across *Drosophila* species using MUSCLE with default parameters (gap opening penalty = −400, gap extension penalty = 0). The CM2 domain was aligned across a range of species using Clustal Omega with default settings (iterations = 3, guide tree clustering). All alignments were manually inspected for conserved motifs.

Sequence conservation was quantified using Jalview (v2.11.4.1) and visualised with colour mapping based on percentage identity (thresholds: ≥90% = dark blue, ≤30% = white).

### SEC-MALS analysis

SEC-MALS experiments were performed using a Superdex 75 10/300 Increase column or Superdex 200 10/300 increase column (Cytiva) and an AktaPure 25 System (Cytiva). The protein sample (100 μL) was loaded onto the gel filtration column and eluted with one column volume (24 mL) of buffer containing 20 mM Tris pH 7.5, 150 mM NaCl, and supplemented with 0.5 mM TCEP, at a flow rate of 0.5 mL/min. The eluting protein was monitored using a DAWN HELEOS-II 18-angle light scattering detector (Wyatt Technologies), a U9-M UV/Vis detector (Cytiva), and an Optilab T-rEX refractive index monitor (Wyatt Technologies). Data were analysed by using Astra v7 (Wyatt Technologies) with a refractive increment value of 0.185 mL/g. Figs were generated using GraphPad Prism (version 10.4.2).

### Negative staining electron microscopy

Freshly glow-discharged TEM grids (C267, TAAB) were placed carbon-side down onto 10 µl droplets of sample containing the complex Cnn 490–608/Cnn CM21077-1148 at a 0.05 mg/ml concentration, resulting after SEC purification and incubated for 2 min. The grids (TAAB) were then blotted and incubated for 10 s on a 20 µl droplet of aqueous uranyl acetate 2% (w/v), then blotted and allowed to air dry. Grids were imaged using a JEOL Flash 120 kV TEM equipped with a Gatan Rio camera.

### Molecular dynamics simulations

Coarse-grained molecular dynamics (MD) simulations were performed using the Martini 3.0.0 force field for proteins and solvent and the corresponding Martini 3.0.0_v1 ion parameters (Souza et al, 2021), as implemented in the CHARMM-GUI Martini Solution Maker (Jo et al, 2008; Qi et al, 2015). We used the AlphaFold2-predicted structure of the Cnn PReM domain dimer (residues 490–608) as the starting model for all simulations, as this contained all three helices of the helical hairpin structure. Point mutations were introduced in CHARMM-GUI to generate the 2A and 2E variants from this common wild-type template. Protein termini were set to be neutral, side-chain protonation states chosen to correspond to pH 7.5, and each system was solvated in Martini water with NaCl added to a final concentration of 0.15 M to match the pH and buffer conditions used experimentally. An elastic network between protein backbone beads was applied using the default CHARMM-GUI settings to preserve the overall fold while allowing relative motions of the helices.

Simulations were carried out using GROMACS 2022.4 (Berendsen et al, 1995) following the default Martini 3 protocol provided by CHARMM-GUI. Systems first underwent two steepest-descent energy minimisation stages (3000 steps each, 20 fs timestep). The first used a soft-core free-energy scheme to gently relieve bad contacts, and the second was a conventional steepest-descent minimisation. No position restraints were applied during either minimisation stage. Minimisation was followed by a 1 ns NPT equilibration run (50,000 steps, 20 fs timestep), with no NVT stage, using position restraints on protein beads at a force constant of 4000 kJ mol⁻¹ nm⁻². Production simulations were performed in the NPT ensemble without position restraints, for 200 ns per replicate using a 20 fs timestep.

Temperature was maintained at 303.15 K using the velocity-rescale (V-rescale) thermostat with a coupling constant of 1.0 ps applied separately to protein and solvent. Pressure was controlled at 1 bar using an isotropic Berendsen barostat with a coupling constant of 5.0 ps during minimisation and equilibration, and an isotropic Parrinello–Rahman barostat with a coupling constant of 12.0 ps during production. Non‑bonded interactions followed the standard Martini 3 settings, using Verlet neighbour lists, reaction‑field electrostatics and 1.1 nm potential‑shifted Lennard–Jones cutoffs. Periodic boundary conditions were applied in all three dimensions. For each construct (WT, 2 A and 2E), three replicas were simulated. All replicas started from the same prepared and solvated structure with the same initial velocities, and were each independently equilibrated and run as separate simulations.

### Statistical analysis

The details of statistical tests, sample size, and definition of the centre and dispersion are provided in individual figure legends.

## Supplementary information


Table EV1
Table EV2
Table EV3
Table EV4
Peer Review File
Movie EV1
Expanded View Figures


## Data Availability

All raw imaging data have been deposited at the BioImage Archive (Hartley et al, 2022) (https://www.ebi.ac.uk/biostudies/bioimages/studies/S-BIAD3684) and all non-imaging source data at (https://zenodo.org/records/20834842). The mass spectrometry proteomics data have been deposited at the ProteomeXchange Consortium (https://www.proteomexchange.org/) via the PRIDE (Perez-Riverol et al, 2025) partner repository with the dataset identifier PXD080319. The atomic coordinates of Cnn_490-579_ WT and Cnn_490-579_ 2A dimers have been deposited into the PDB (https://www.rcsb.org/) with the accession numbers: 9T4T and 9S7L, respectively. The source data of this paper are collected in the following database record: biostudies:S-SCDT-10_1038-S44318-026-00878-x.
