# Supplementary material for: Polo/PLK1 phosphorylation relieves Centrosomin/Cnn autoinhibition to promote centrosome scaffold assembly
Source: EMBO J. 2026 Jul 30;45(17):6320–56. doi: 10.1038/s44318-026-00878-x (PMC13534702; doi:10.1038/s44318-026-00878-x)
Supplement: Supplementary file 1 [file 44318_2026_878_MOESM5_ESM.pdf]

Polo/PLK1 phosphorylation relieves Centrosomin/Cnn autoinhibition to promote centrosome scaffold assembly

Nada Mohamad, Siu-Shing Wong, Anupa Majumdar, alan wainman, Ingelise Holland-Kaye, Lars Hubatsch, Zsofia Novak, Andrei Pozniakovsky, Martine Ruer-Gruss, Andreas Haensele, Anna Caballe, Steven Johnson, Susan Lea, Anthony Hyman, and Jordan Raff

Corresponding author(s): Jordan Raff (jordan.raff@path.ox.ac.uk) , Susan Lea (susan.lea@stjude.org), Anthony Hyman (hyman@mpi-cbg.de)

Review Timeline:

Transfer from Review Commons:	15th Mar 26
Editorial Decision:	12th May 26
Revision Received:	23rd Jun 26
Accepted:	9th Jul 26

Editor: Hartmut Vodermaier

Transaction Report: **This manuscript was transferred to The EMBO JOURNAL following peer review at Review Commons.**

Review #1

1. Evidence, reproducibility and clarity:

Evidence, reproducibility and clarity (Required)

The study by Mohamad et al. investigates the structural basis and regulatory role of phosphorylation in the assembly of the mitotic pericentriolar material (PCM) scaffold, which nucleates microtubules and organizes the poles of the mitotic spindle. They use structure determination, biochemical reconstitution and in vivo experiment in flies to address how fly, worm, and human homologs of a key scaffold protein (Cnn, SPD-5, and CDK5RAP2, respectively) are relieved from auto-inhibition in a phosphorylation-dependent manner to form extended scaffolds through interactions between PReM and CM2 domains. An important discovery is a helical hairpin structure in the PReM domain that is the basis of autoinhibition and is regulated by phosphorylation. The work addresses the fundamental question how the centrosome matures in preparation for mitosis, by increasing the size and activity of the PCM scaffold that surrounds the centrioles. It also addresses how conserved the underlying molecular mechanism are among flies, worms, and humans. The study is overall of high quality, building on previous works by the authors and other groups, and adding new structural and biochemical insight. Most of the conclusions are supported by the data. I have a few concerns though that should be addressed.
An important issue is the analysis of phosphorylation sites, which appears incomplete. For example, it lacks demonstration that both of the two studied phosphorylation sites are indeed phosphorylated. Kinase motif identification and mutation is not sufficient, considering that phosphorylation is integral to the proposed model of how autoinhibitory intra-molecule interactions are relieved, and considering that phospho-mimetics have not been tested in vitro and function poorly in vivo.

Main:

1) From previous studies, it seems to me that for the residues potentially relevant for the hairpin regulation there is direct evidence of phosphorylation only for S567 (mass spec, phospho-antibody). Have the authors tested single site mutants (S567A and E)? Also, have they tested D mutations? If so, this should be commented on and shown. If not, it should be tested, in particular since the 2E phospho-mimetic is not functioning properly in vivo. If S571 is indeed crucial, it should be demonstrated that it is also phosphorylated. Otherwise it is possible that the mutation of this residue simply impairs important interactions (e.g. PReM-CM2, others), independent of phosphorylation.

2) It is unclear why in vitro only A mutations have been tested and not phospho-mimetics. This should be tested for the interaction between PReM and CM2. This would allow to probe the model that phosphorylation opens the hairpin to allow interaction. Currently, such proof is missing in the study. Alternatively, the authors could phosphorylate the recombinant protein in vitro. The in vivo data is harder to interpret due to the complexity of the model and the authors should take advantage of the in vitro system.

3) Regarding the worm PReM and CM2 domains, the authors mention that they have tested in vitro phosphorylation by PLK-1, but I could not find any data showing this. They should demonstrate successful phosphorylation or test candidate site by phospho-mimetic mutation. It is possible that the worm proteins depend more strongly on phosphorylation to relieve autoinhibition compared to the fly proteins.

Minor:

4). Fig. 6C, D: the labeling of the chimeric constructs using "+" symbols is confusing, since it suggests that separate proteins were expressed. If I understand this correctly, with the current labeling, deltaCM2+DmCM2 means WT? The authors should write the full name of the wildtype or chimeric construct in each case and use a more standard/less confusing nomenclature. Also, I suggest to start the panels and graphs with the WT sample.

2. Significance:

Significance (Required)

The study's strength is the use of a combination of structural and biochemical approaches with in vivo model testing. Its main limitation is that the analyses of the role of phosphorylation lacks depth and is not fully conclusive, despite its importance for centrosomal scaffold assembly.
The study advances our understanding of centrosomal scaffold assembly and maturation at a molecular level, and how specific molecular aspects of these processes are conserved or differ among different organisms.
The findings are of interest to cell biologists. My expertise is in centrosome and microtubule biology.

3. How much time do you estimate the authors will need to complete the suggested revisions:

Estimated time to Complete Revisions (Required)

(Decision Recommendation)

Between 3 and 6 months

4. *Review Commons* values the work of reviewers and encourages them to get credit for their work. Select 'Yes' below to register your reviewing activity at [Web of Science Reviewer Recognition Service]() (formerly Publons); note that the content of your review will not be visible on Web of Science.

Web of Science Reviewer Recognition

Yes

Review #2

1. Evidence, reproducibility and clarity:

Evidence, reproducibility and clarity (Required)

Summary:

Mohamed et al. set out to compare the assembly mechanisms of pericentriolar material (PCM) in flies and nematodes. They reveal that the main PCM scaffold protein in each species (Cnn in flies, SPD-5 in nematodes) are sufficient to form supramolecular droplets (with a crowding agent) or networks (without a crowding agent). However, they diverge in one key aspect: Cnn scaffold assembly relies on the interaction between a C-terminal CM2 domain and a central phospho-regulated domain (PReM), whereas SPD-5 does not. The authors solve the crystal structure of a region within Cnn's PReM. With the help of modeling, they speculate that this region is auto-inhibited through backfolding of alpha helices, thus preventing its interaction with the CM2 domain. This auto-inhibition would be relieved by phosphorylation, which modeling suggests would increase "breathing" of the backfolded structure. The author end by presenting evidence to suggest that the human PCM scaffold protein CDK5RAP2 may assemble through a PReM-CM2 interaction.

Major Comments:

1. The title is too vague. Any number of existing papers could be said to provide "structural insights into mitotic centrosome assembly". The authors need to narrow down to a defined

conclusion and state this as the title.
2. I think the strongest and most novel aspects of this study relate to the mechanism of Cnn assembly via relief of the auto-inhibited PReM. The effort to elucidate assembly mechanisms of SPD-5 and CDK5RAP2 are comparatively light and there are no accompanying experiments in worms or human cells. Without the in vivo experiments, it's hard to know if the in vitro experiments are valid. It's speculative for the authors to say they found the true PReM for CDK5RAP2; they do not demonstrate that PLK-1 phosphorylation potentiates assembly in Figure 8.
Thus, I suggest re-writing the paper to focus on Cnn. Experiments in Figure 6 are still valid if reframed. For example, substituting Cnn's CM2 with the CM2 from CDK5RAP2 vs. the C-term of SPD-5 illustrates that a simple coiled-coil with open ends (H.s.CM2) is sufficient to interact with PReM whereas a coiled-coil with a closed end (SPD-5 C-term, predicted by Figure 6A) cannot.
3. The purpose of Figure 1 is unclear. None of the other figures examine SPD-5 and CNN in the condensate form, which required using 4% PEG in this paper. The other assays look at the network form, which could behave differently and have different dependence on specific domains. I think they should perform the condensate assay for all other figures, otherwise leave it out. Furthermore, CDK5RAP2 is mentioned, yet not examined in Figure 1. It must be noted that CDK5RAP2 will also condense into droplets under crowding conditions or with a synthetic nucleator (Rios et al., 2025 J Cell Sci). Thus, it seems that condensation potential is a universal feature of known PCM scaffold proteins.
4. The study uses different species without doing the same types of experiments on each. Sometimes human CDK5RAP2 is thrown in, sometimes not. They solve crystal structures of PReM from Cnn but not from the other proteins. This gets confusing, especially since the authors state that they seek to test if fly Cnn and worm SPD-5 assemble through different mechanisms (see last sentence of the intro). Also, if the focus is on worm vs. fly PCM assembly mechanisms, why include the human protein, especially Figure 8?
5. The conclusion that SPD-5's narrow PReM and "CM2" domains don't interact is consistent with the cross-linking mass spectrometry data from Rios et al. 2024. They showed only one X-link with low occurrence (1 out of 6 samples) between these two regions, even in the phosphorylated state (Fig. 1G). However, Nakajo et al (2022) claimed the opposite, showing that a larger PReM-containing construct (a.a. 272-732) interacts with a C-terminal construct (a.a. 1061-1198) after PLK-1 phosphorylation. Can the authors comment on this? Perhaps there is another site in SPD-5, outside of a.a. 541-677, that acts like the Cnn PReM?
6. I have serious doubts that the C-terminus of SPD-5 has a CM2 domain. To me, there is no real sequence homology with the traditional CM2's from humans and flies, and the AF3 predictions support this. Ohta et al. (2021) called this region "CM2-like" based on very poor

homology, which a is questionable practice. Any coiled-coil region will appear somewhat homologous due to the heptad repeat pattern that defines them (e.g., leucines line up quite nicely). Thus, is it fair to say that SPD-5 doesn't assemble through a PReM-CM2 interaction? There may be a different region in SPD-5 that looks more like the canonical CM2. I think the authors have compelling evidence to give the C-terminal coiled-coil region in SPD-5 its own name rather than calling it CM2.
7. Figure 3E. Would measuring scaffold mass be more appropriate? The PReM(deltaH1,NTH2) leads to more compact scaffolds, but maybe they assemble just as well as the deltaH1 mutant. As it stands, there is a discrepancy between panel E and F in terms of what is measured (area vs. intensity) and the outcome.

Minor Comments

1. In one version of the PDF there are images missing in Fig 1F, 4C, 4D. I opened another version (source version) and the images were there. Just FYI.
2. Figure 4A. The blue coloration makes it difficult to read the black letters.
3. Figure 4A. Why is part of the protein colored in green? This coloration isn't defined, nor does it show up again in panel B.
4. The layout of Figure 4 is confusing. It took me a few minutes to realize that the big red box inset belonged to panel B and not panel A.
5. Figure 4C,D. The sample size is not mentioned in the legend.
6. The title for Figure 4 seems too speculative. How can the authors say that phosphorylation relieves the autoinhibition without structural data?
7. Figure 5B. The sample size is not mentioned in the legend.
8. Figure 6B,D. The sample size is not mentioned in the legend.
9. The text in Figure 7B is hard to read because it is too small. Please make this bigger.
10. Figure 8C. What is colored in magenta? Is there an additional labeled protein besides mNG-CM2?
11. Figure 8C. What is the sample size? How many images were taken? Also, why are there data points off to the right of the last column?
12. The wording of these sections needs improving. I found them complicated and difficult to understand.

"Fly and worm Spd-2/SPD-2 and Polo/PLK-1 are clear homologues, but Cnn and SPD-5 share little sequence homology-although they are both predicted to be large coiled-coil-rich proteins. Thus, it remains unclear whether these two, largely unrelated, molecules form mitotic-PCM scaffolds that assemble and function in a similar manner"

"We first focused on Drosophila Cnn as, although the full structure of the original PReM domain (Cnn403-608) is unknown, this domain contains an internal leucine-zipper (LZ) dimer (Cnn490-544) whose crystal structure, in a tetrameric complex with a CM2 dimer, had been solved (Figure 2A) (Feng et al., 2017)."

"When the full PReM and CM2 domains are mixed in vitro, they form large micron-scale assemblies and point mutations that perturb the LZ::CM2 tetramer perturb PReM::CM2 scaffold assembly in vitro and Cnn scaffold assembly in vivo."

2. Significance:

Significance (Required)

Overall Assessment:

While I find the premise of this study to be interesting, its execution and presentation are not fully convincing. The study is a collection of experiments connected by a thread that can be difficult to follow. One concern is the lack of focus and a clearly stated conclusion, which is ultimately embodied by the vague title. For example, the research question at the beginning doesn't match with the outcome in the end. At the end of the introduction, the authors state they wish to compare assembly mechanisms of Cnn and SPD-5. However, at the end of the results, they present data on CDK5RAP2 and speculate on its assembly. Why introduce the human protein here? Another concern is the lack of symmetry in the experiments. There is much more in vitro characterization of Cnn than SPD-5 or CDK5RAP2, and all in vivo work is performed in flies. Finally, this study does not address if the best-established model for SPD-5 assembly-multimerization via specific, multivalent coiled-coil interactions-applies to fly Cnn. Thus, to me, this is study is a deeper dive into the mechanism of Cnn assembly, not necessarily a fair cross-species comparison. I do not have major issues with the results, but I recommend that this paper undergo significant re-writing before being re-reviewed. There are also issues with data display and reporting of experimental details (e.g., sample sizes) that should be easily fixed.

Advance: this study provides new insight into how two specific domains interact within PCM scaffold proteins to promote scaffold assembly. It provides some new structural insight into the mechanism of Cnn auto-inhibition. However, there is limited conceptual advance, as the bigger ideas (e.g., auto-inhibition as a regulatory control, PCM scaffold assembly through condensation of coiled-coil proteins) were already established.

Audience: this study will be of interest to cell biologists studying centrosome assembly, mitosis, and evolution.

3. How much time do you estimate the authors will need to complete the suggested revisions:

Estimated time to Complete Revisions (Required)

(Decision Recommendation)

Between 1 and 3 months

4. *Review Commons* values the work of reviewers and encourages them to get credit for their work. Select 'Yes' below to register your reviewing activity at [Web of Science Reviewer Recognition Service]() (formerly Publons); note that the content of your review will not be visible on Web of Science.

Web of Science Reviewer Recognition

No

Review #3

1. Evidence, reproducibility and clarity:

Evidence, reproducibility and clarity (Required)

This study by Mohamad et al. builds on prior work by Conduit et al. (2014, PMID: 24656740) and Feng et al. (2017, PMID: 28575671), which established the essential role of intramolecular interactions between the phospho-regulated multimerization (PReM) domain and centrosomin motif 2 (CM2) of Drosophila Cnn in pericentriolar matrix (PCM) expansion during mitosis. Extending these studies, the authors investigate the structural properties of Cnn's PReM and CM2 domains and compare them with homologous proteins in C. elegans (SPD-5) and humans (CDK5RAP2). Their analyses suggest a phosphorylation-dependent mechanism that relieves Cnn autoinhibition, with particular emphasis on Ser567 and Ser571 within the PReM domain. The authors further propose that, whereas Cnn and CDK5RAP2 share conserved CM2-PReM interactions, SPD-5 has diverged to employ distinct mechanisms for PCM scaffold assembly.

Although these conclusions rely heavily on AlphaFold3-predicted models (Abramson et al.,

2024, PMID: 38718835), they are supported by a combination of in vitro and in vivo experiments, including live-cell imaging and molecular dynamics simulations. However, inconsistencies between in vitro and in vivo observations weaken some interpretations and warrant more careful discussion. Addressing the concerns below would substantially strengthen the manuscript.

Major Comments

1. The title, "Structural Insights into Mitotic-Centrosome Assembly," is overly broad. The study primarily focuses on CM2-PReM intramolecular interactions in D. melanogaster Cnn and does not comprehensively address mitotic centrosome assembly across species. A more specific title reflecting the fly-centric and structural focus would better align with the manuscript's scope and conclusions.
2. The authors analyze condensate formation by Cnn and SPD-5 but overlook condensate formation by CDK5RAP2, which was recently reported by Rios et al. (2025, PMID: 40454523). Including CDK5RAP2 would enable a more balanced and informative comparison across fly, worm, and human homologs.
3. In Figure 3, reconstitution of Cnn scaffolds using purified CM2 and PReM fragments yields "macromolecular scaffolds," but their physical properties are not defined. It remains unclear whether these assemblies are ordered or amorphous, and whether they exhibit solid- or gel-like behavior. Moreover, the heterogeneous, scattering particles observed by negative-stain EM (Figure S3B), likely corresponding to the Cnn490-608-CM2 complex, raise the possibility of nonspecific aggregation rather than organized scaffold formation. Appropriate controls lacking CM2 are needed to exclude spontaneous aggregation of PReM fragments. In addition, testing shorter truncations of the PReM H2 helix could help define the minimal requirements for scaffold assembly. Finally, the rationale for including the CnnΔExPReM construct only in vivo (Figure 3F), but not in the in vitro assays (Figure 3A-E), should be clarified.
4. The coarse-grained (CG) simulation methodology is insufficiently described. Given that CG approaches sacrifice atomic detail and may oversimplify interactions, readers require more information to evaluate the model's reliability and limitations. A comparison with the framework used by Ramirez et al. (2024, PMID: 38356260) would be informative. It is also unclear why available crystal structures of WT and 2A Cnn (Figure 2C; Figure S4) were not used as simulation inputs, or why the structure of Cnn490-579 2E was not determined to complete the structural comparison.

Furthermore, mutation of Ser567 and Ser571 to alanine markedly stabilizes the PReM domain (Figure 5C, D), implying that these residues maintain domain flexibility. Back-

mapping CG models to atomic resolution could reveal the interactions altered by these mutations. The exclusive focus on double mutants (2A and 2E) is also limiting; analysis of single-point mutants at S567 or S571 would clarify whether both residues contribute equally or play distinct roles.
5. The discussion lacks sufficient integration with prior studies and often presents conclusions without adequate citation. For example, the claim that flies and humans rely on related PReM-CM2 interactions whereas worms use distinct phosphorylation-regulated mechanisms is not supported by appropriate references. In addition, limited cross-referencing to the manuscript's own data weakens the connection between results and conclusions. Expanding and better grounding the discussion in existing literature would significantly enhance its depth and clarity.

Minor Comments

1. In Figure 1B, the molecular weight units for the protein marker are missing and should be included.
2. In Figures 1E and 1F, readability would be improved by including x-axis labels on all graphs, rather than only on the bottom panels.
3. The protein structures shown in Figures 2C and 2D should be explicitly labeled as dimers to avoid confusion.
4. In Figures 3A-D, using fluorescently labeled CM2 would help validate both the interaction with the PReM domain and its localization within the scaffold.
5. In Figure 3E, no statistical comparisons are presented between the original PReM construct and other samples. In addition, information regarding sample size and the number of experimental replicates is missing from the figure legend.
6. In Figure 3F, the absence of a pixel intensity scale bar makes the data difficult to interpret, as color values corresponding to high and low signal intensities are unclear. Moreover, no additional centrosome marker is included, nor is there evidence that PReM fragment expression levels are comparable across samples. These concerns also apply to Figures 4C and 4D.
7. In Figure 4A, the interacting residues of PReM and CM2 shown in the red inset would be clearer if residue annotations for each domain were displayed in distinct colors. Additionally, the legends for Figures 4C and 4D do not specify the scale bar length.
8. The authors state that interactions between CM2 and PReM-2A462-608 could not be detected in vitro based on SEC chromatograms (Figure 5A), yet the figure does not clearly show this result. The accompanying SDS-PAGE images are too small and lack lane labels, making interpretation difficult (a similar issue applies to Figure 7B). Furthermore, the SEC chromatogram x-axis lacks volume annotations, hindering correlation between

chromatographic peaks and SDS-PAGE results (in contrast to Figure 7B, which provides an appropriate example).

2. Significance:

Significance (Required)

This work will be of interest not only to cell biologists studying centrosomes, but also to molecular biologists investigating how protein modifications regulate protein behavior.

3. How much time do you estimate the authors will need to complete the suggested revisions:

Estimated time to Complete Revisions (Required)

(Decision Recommendation)

Between 1 and 3 months

4. *Review Commons* values the work of reviewers and encourages them to get credit for their work. Select 'Yes' below to register your reviewing activity at Web of Science Reviewer Recognition Service (formerly Publons); note that the content of your review will not be visible on Web of Science.

Web of Science Reviewer Recognition

Yes

Full Revision

Manuscript number: RC-2025-03287
Corresponding author(s): Jordan, Raff; Anthony, Hyman; Susan, Lea

1. General Statements [optional]

We thank the Reviewers for their comments on our manuscript "Structural insights into mitotic-centrosome assembly". As described below, we have substantially revised the manuscript in response to their comments and are hoping you would consider the revised manuscript "Phosphorylation relieves autoinhibition to drive Cnn centrosome scaffold assembly" at *The EMBO Journal*. Our specific responses (*black text*) to the Reviewer's comments (*blue text*) are detailed below

2. Point-by-point description of the revisions

This section is mandatory. Please insert a point-by-point reply describing the revisions that were already carried out and included in the transferred manuscript.

Reviewer #1

Main Points:

1) From previous studies, it seems to me that for the residues potentially relevant for the hairpin regulation there is direct evidence of phosphorylation only for S567 (mass spec, phospho-antibody). Have the authors tested single site mutants (S567A and E)? Also, have they tested D mutations? If so, this should be commented on and shown. If not, it should be tested, in particular since the 2E phospho-mimetic is not functioning properly in vivo. If S571 is indeed crucial, it should be demonstrated that it is also phosphorylated. Otherwise it is possible that the mutation of this residue simply impairs important interactions (e.g. PReM-CM2, others), independent of phosphorylation.

As requested, we have now tested individual S567A and S571A mutations and found that they both perturb Cnn scaffold assembly, but to a lesser extent than the 2A double mutant (New Fig.S3A). We also now confirm by MS that recombinant Polo can phosphorylate both S567 and S571 *in vitro*, and we have examined the behaviour of a 2D mutant and find that it behaves very similarly to the 2E mutant (New Fig.S3B).

2) It is unclear why in vitro only A mutations have been tested and not phospho-mimetics. This should be tested for the interaction between PReM and CM2. This would allow to probe the model that phosphorylation opens the hairpin to allow interaction. Currently, such proof is missing in the study. Alternatively, the authors could phosphorylate the recombinant protein in vitro. The in vivo data is harder to interpret due to the complexity of the model and the authors should take advantage of the in vitro system.

As requested, we now show in New Fig.S5 that whereas *in vitro* WT $Cnn_{490-608}$ and $Cnn\text{-}2A_{490-608}$ behave as dimers, $Cnn\text{-}2E_{490-608}$ elutes in two major fractions—a tetramer species and a much larger species that elutes in the void volume (meaning that 2E can form very large species even in the absence of CM2) (Figure S5A). In the presence of CM2, $Cnn\text{-}2E_{490-608}$ forms a tetramer (that eluted slightly later than the $Cnn\text{-}2E_{490-608}$ tetramer) and larger complexes that contained CM2 and eluted in the void volume with a profile similar to $Cnn\text{-}2E_{490-608}$ on its own (Figure S5B). These results are consistent with the possibility that the 2E substitutions open the helical hairpin to allow self-interactions that drive homo-tetramer and larger complex assembly *in vitro*.

3) Regarding the worm PReM and CM2 domains, the authors mention that they have tested in vitro phosphorylation by PLK-1, but I could not find any data showing this. They should demonstrate successful phosphorylation or test candidate site by phospho-mimetic mutation. It is possible that the worm proteins depend more strongly on phosphorylation to relieve autoinhibition compared to the fly proteins.
This is a good point, and we apologise for this omission. We now state that we confirmed by MS analysis that the recombinant worm PLK-1 we used in these *in vitro* experiments phosphorylates the putative SPD-5 PReM domain on the three sites (S627, S653 and S658) known to be important for promoting SPD-5 scaffold assembly *in vivo* (Figure Legend, Figure 6). Thus, the lack of detectable binding between these proteins is not due to the lack of phosphorylation.

Minor Point:
4). Fig. 6C, D: the labeling of the chimeric constructs using "+" symbols is confusing, since it suggests that separate proteins were expressed. If I understand this correctly, with the current labeling, deltaCM2+DmCM2 means WT? The authors should write the full name of the wildtype or chimeric construct in each case and use a more standard/less confusing nomenclature. Also, I suggest to start the panels and graphs with the WT sample.
We thank the Reviewer for this suggestion and have re-labelled this Figure to clarify this point. We understand the point about putting the WT panels first in Figure 6C,D (now Figure 5C,D) but think that this is not the correct comparison to emphasise. We are testing the ability of the various CM2 domains to “rescue” the lack of a CM2 domain, so we feel *Drosophila* Cnn lacking CM2 is the correct baseline for this comparison.

Reviewer #2

Main Comments:
1. The title is too vague. Any number of existing papers could be said to provide "structural insights into mitotic centrosome assembly". The authors need to narrow down to a defined conclusion and state this as the title.
2. I think the strongest and most novel aspects of this study relate to the mechanism of Cnn assembly via relief of the auto-inhibited PReM. The effort to elucidate assembly mechanisms of SPD-5 and CDK5RAP2 are comparatively light and there are no accompanying experiments in worms or human cells. Without the in vivo experiments, it's hard to know if the in vitro experiments are valid. It's speculative for the authors to say they found the true PReM for CDK5RAP2; they

do not demonstrate that PLK-1 phosphorylation potentiates assembly in Figure 8. Thus, I suggest re-writing the paper to focus on Cnn. Experiments in Figure 6 are still valid if reframed. For example, substituting Cnn's CM2 with the CM2 from CDK5RAP2 vs. the C-term of SPD-5 illustrates that a simple coiled-coil with open ends (H.s.CM2) is sufficient to interact with PReM whereas a coiled-coil with a closed end (SPD-5 C-term, predicted by Figure 6A) cannot.
We thank the Reviewer for these helpful comments and have re-written and re-organised the manuscript in accord with these suggestions—most importantly providing a more specific title and re-ordering the data to better focus the paper on the relief of Cnn autoinhibition.

3. The purpose of Figure 1 is unclear. None of the other figures examine SPD-5 and CNN in the condensate form, which required using 4% PEG in this paper. The other assays look at the network form, which could behave differently and have different dependence on specific domains. I think they should perform the condensate assay for all other figures, otherwise leave it out. Furthermore, CDK5RAP2 is mentioned, yet not examined in Figure 1. It must be noted that CDK5RAP2 will also condense into droplets under crowding conditions or with a synthetic nucleator (Rios et al., 2025 J Cell Sci). Thus, it seems that condensation potential is a universal feature of known PCM scaffold proteins.
The original Figure 1 has been moved to end of the paper (now Figure 8) and we now more thoroughly explain the logic of these experiments. Briefly, given that the PReM and CM2 domains in flies and worms seem to function in different ways *in vivo*, we sought here to test whether this was also the case *in vitro*—where the behaviour of full-length SPD-5 and of these domains of Cnn have been extensively studied, but never directly compared. We believe such a direct comparison will be of some interest to the field (the Woodruff et al., 2017 paper describing these *in vitro* SPD-5 condensates has been cited >700 times). We now also cite the Rios et al., 2025 paper but note that, despite extensive efforts, we were unable to purify enough well-behaved CDK5RAP2 for our experiments and so could not include it in this analysis. We think Rios et al., used an MBP-fusion of CDK5RAP2 in their experiments, which may explain this difference.

4. The study uses different species without doing the same types of experiments on each. Sometimes human CDK5RAP2 is thrown in, sometimes not. They solve crystal structures of PReM from Cnn but not from the other proteins. This gets confusing, especially since the authors state that they seek to test if fly Cnn and worm SPD-5 assemble through different mechanisms (see last sentence of the intro). Also, if the focus is on worm vs. fly PCM assembly mechanisms, why include the human protein, especially Figure 8?
On re-reading our original manuscript we appreciate this confusion. We hope that in re-writing the manuscript along the lines suggested by the Reviewer the logical flow of our experiments will be clearer.

5. The conclusion that SPD-5's narrow PReM and "CM2" domains don't interact is consistent with the cross-linking mass spectrometry data from Rios et al. 2024. They showed only one X-link with low occurrence (1 out of 6 samples) between these two regions, even in the phosphorylated state (Fig. 1G). However, Nakajo et al (2022) claimed the opposite, showing that a larger PReM-containing construct (a.a. 272-732) interacts with a C-terminal construct (a.a. 1061-1198) after

PLK-1 phosphorylation. Can the authors comment on this? Perhaps there is another site in SPD-5, outside of a.a. 541-677, that acts like the Cnn PReM?
These are good points and we now mention this last possibility in the Discussion. We also now mention the supporting cross-linking Mass Spec data from Rios et al., 2024.

6. I have serious doubts that the C-terminus of SPD-5 has a CM2 domain. To me, there is no real sequence homology with the traditional CM2's from humans and flies, and the AF3 predictions support this. Ohta et al. (2021) called this region "CM2-like" based on very poor homology, which a is questionable practice. Any coiled-coil region will appear somewhat homologous due to the heptad repeat pattern that defines them (e.g., leucines line up quite nicely). Thus, is it fair to say that SPD-5 doesn't assemble through a PReM-CM2 interaction? There may be a different region in SPD-5 that looks more like the canonical CM2. I think the authors have compelling evidence to give the C-terminal coiled-coil region in SPD-5 its own name rather than calling it CM2.
This is a fair point, although the literature is already quite confusing on the nomenclature for the C-terminal region of SPD-5 (e.g., Ohta et al., JCB, 2021; Nakajo et al., JCS, 2022), so we are reluctant to add another name to the mix. Given that we draw comparisons with the fly and human CM2 domains (that are clearly related by sequence), we think it is easiest for readers if we use the “CM2” nomenclature throughout, although making clear our conclusion that SPD-5 “CM2” does not appear to function in the same way as fly/human CM2.

7. Figure 3E. Would measuring scaffold mass be more appropriate? The PReM(deltaH1,NTH2) leads to more compact scaffolds, but maybe they assemble just as well as the deltaH1 mutant. As it stands, there is a discrepancy between panel E and F in terms of what is measured (area vs. intensity) and the outcome.
In several previous papers we use fluorescence intensity to measure the “amount” of protein at centrosomes *in vivo* but, in our original paper (Feng et al., *Cell*, 2017), we quantified PReM::CM2 scaffold assembly *in vitro* by measuring the area of scaffold assembly. Thus, we prefer to present the current data in this way for consistency across publications, and we believe either measure is valid. We could measure the area and intensity of the PReMΔH1 and PReMΔH1ΔNTH2 scaffolds to compare scaffold density, but we think this would unnecessarily complicate this data. The main point is not how much or how dense each scaffold is, but rather that the PReMΔH1ΔNTH2 protein doesn’t really make a scaffold at all—but rather makes smaller “blobs” that tend to bunch together (further characterised in Fig.S2).

Minor Comments:

1. In one version of the PDF there are images missing in Fig 1F, 4C, 4D. I opened another version (source version) and the images were there. Just FYI.
2. Figure 4A. The blue coloration makes it difficult to read the black letters.
3. Figure 4A. Why is part of the protein colored in green? This coloration isn't defined, nor does it show up again in panel B.
4. The layout of Figure 4 is confusing. It took me a few minutes to realize that the big red box inset belonged to panel B and not panel A.

5. Figure 4C,D. The sample size is not mentioned in the legend.
6. The title for Figure 4 seems too speculative. How can the authors say that phosphorylation relieves the autoinhibition without structural data?
7. Figure 5B. The sample size is not mentioned in the legend.
8. Figure 6B,D. The sample size is not mentioned in the legend.
9. The text in Figure 7B is hard to read because it is too small. Please make this bigger.
10. Figure 8C. What is colored in magenta? Is there an additional labeled protein besides mNG-CM2?
11. Figure 8C. What is the sample size? How many images were taken? Also, why are there data points off to the right of the last column?
12. The wording of these sections needs improving. I found them complicated and difficult to understand.

We thank the Reviewer for taking the time to make these helpful comments. We have addressed all these points in the revised manuscript. On point 10, the magenta objects were fiduciary beads that were inadvertently included on this panel (and are no longer shown).

Reviewer #3

Major Comments:

1. The title, "Structural Insights into Mitotic-Centrosome Assembly," is overly broad. The study primarily focuses on CM2-PReM intramolecular interactions in D. melanogaster Cnn and does not comprehensively address mitotic centrosome assembly across species. A more specific title reflecting the fly-centric and structural focus would better align with the manuscript's scope and conclusions.

As described at the start of our response to Reviewer #2, the title and focus of the manuscript have been extensively revised along these lines.

2. The authors analyze condensate formation by Cnn and SPD-5 but overlook condensate formation by CDK5RAP2, which was recently reported by Rios et al. (2025, PMID: 40454523). Including CDK5RAP2 would enable a more balanced and informative comparison across fly, worm, and human homologs.

As described in point 3 of our response to Reviewer #2, we now cite Rios et al., 2025 but note that, despite extensive efforts, we were unable to purify enough well-behaved CDK5RAP2 for our experiments and so could not include it in this analysis. We believe Rios et al., used a full-length MBP-fusion of CDK5RAP2 in their experiments, which may explain this difference as MBP is very good at keeping proteins soluble (but would not be appropriate in our experiments where we compare full-length untagged proteins).

3. In Figure 3, reconstitution of Cnn scaffolds using purified CM2 and PReM fragments yields "macromolecular scaffolds," but their physical properties are not defined. It remains unclear whether these assemblies are ordered or amorphous, and whether they exhibit solid- or gel-like

behavior. Moreover, the heterogeneous, scattering particles observed by negative-stain EM (Figure S3B), likely corresponding to the Cnn490-608-CM2 complex, raise the possibility of nonspecific aggregation rather than organized scaffold formation. Appropriate controls lacking CM2 are needed to exclude spontaneous aggregation of PReM fragments. In addition, testing shorter truncations of the PReM H2 helix could help define the minimal requirements for scaffold assembly. Finally, the rationale for including the CnnΔExPReM construct only in vivo (Figure 3F), but not in the in vitro assays (Figure 3A-E), should be clarified.

We apologise, as our presentation of this data has clearly led to some confusion on these points.

First, as we now clarify, the amorphous solid-like physical properties of the PReM::CM2 scaffolds were described in our previous paper where we also showed that these scaffolds are not simply non-specific aggregates—as several single point mutations that disrupt the LZ::CM2 tetramer also prevent PReM::CM2 scaffold assembly *in vitro* as well as Cnn scaffold assembly *in vivo* (see Fig.5, Feng et al., *Cell*, 2017). Also, in all *in vitro* scaffolding experiments we always perform a negative control (-CM2) to confirm that none of the scaffolds are aggregates of the PReM domain being tested. We don't usually show this control now as there would be lots of empty black boxes on the Figures. We do, however, show this control for the human putative PReM domain (Figure 7C), as we are testing this here for the first time.

Second, the request to test shorter truncations of the PReM H2 helix to define the minimal requirements for scaffold assembly is unnecessary as PReMΔH1ΔNTH2 already cuts H2 at the start of the LZ, and we previously showed the LZ is required for PReM::CM2 scaffold assembly *in vitro* (Feng et al., *Cell*, 2017). Thus, any further truncation of H2 will start to remove the LZ, which we already know is essential. We have now made this point more clearly.

Finally, the CnnΔExPReM construct the Reviewer mentions was tested in both the *in vitro* (now Figure 2B) and *in vivo* (now Figure 2F) assays, but the labelling was confusing so this was not clear. We have now clarified this point.

4. The coarse-grained (CG) simulation methodology is insufficiently described. Given that CG approaches sacrifice atomic detail and may oversimplify interactions, readers require more information to evaluate the model's reliability and limitations. A comparison with the framework used by Ramirez et al. (2024, PMID: 38356260) would be informative. It is also unclear why available crystal structures of WT and 2A Cnn (Figure 2C; Figure S4) were not used as simulation inputs, or why the structure of Cnn490-579 2E was not determined to complete the structural comparison. Furthermore, mutation of Ser567 and Ser571 to alanine markedly stabilizes the PReM domain (Figure 5C, D), implying that these residues maintain domain flexibility. Back-mapping CG models to atomic resolution could reveal the interactions altered by these mutations. The exclusive focus on double mutants (2A and 2E) is also limiting; analysis of single-point mutants at S567 or S571 would clarify whether both residues contribute equally or play distinct roles.

We performed coarse-grained simulations because although they simplify atomic interactions and capture overall conformational dynamics, which is what we are trying to assess here (Fig.4C,D). We now clarify this point and provide more detail of our simulation methodology in the main text and Materials and Methods. We used the full helical hairpin (i.e., H2+H3+H4) prediction in these simulations—rather than the crystal structure of the partial helical hairpin (i.e., H2+most of H3)—as we reasoned that the presence of the full H3 and H4 might influence breathing, and the full helical hairpin (see Video S1) seems likely to be the relevant biological fold. As we now show (new Figure S5), and as discussed above, the 2E mutants do not behave well *in vitro* so we were unable to solve their structure. We agree that we could perform atomic resolution simulations to better understand how the 2A/E and single A/E mutations might suppress/enhance breathing, but we believe such an analysis is beyond the scope of the current manuscript and would distract from our main conclusions.

5. The discussion lacks sufficient integration with prior studies and often presents conclusions without adequate citation. For example, the claim that flies and humans rely on related PReM-CM2 interactions whereas worms use distinct phosphorylation-regulated mechanisms is not supported by appropriate references. In addition, limited cross-referencing to the manuscript's own data weakens the connection between results and conclusions. Expanding and better grounding the discussion in existing literature would significantly enhance its depth and clarity.
We thank the Reviewer for this general point and have tried to better integrate our results with prior studies—particularly in the Discussion section.

<u>Minor Comments:</u>
1. In Figure 1B, the molecular weight units for the protein marker are missing and should be included. Fixed.
2. In Figures 1E and 1F, readability would be improved by including x-axis labels on all graphs, rather than only on the bottom panels. Fixed.
3. The protein structures shown in Figures 2C and 2D sh7w b bʃybb ould be explicitly labeled as dimers to avoid confusion. Fixed.
4. In Figures 3A-D, using fluorescently labeled CM2 would help validate both the interaction with the PReM domain and its localization within the scaffold. We have previously tried fluorescently tagging the CM2 domain, but scaffold formation is much less robust. We do not think this invalidates this assay, as the evidence supporting the PReM::CM2 interaction is very strong—including assessing the physiological influence of multiple point mutations in both domains in residues at the heart of the interaction interface identified by crystallography (e.g., see Fig.4, Feng et al., *Cell*, 2017).

5. In Figure 3E, no statistical comparisons are presented between the original PReM construct and other samples. In addition, information regarding sample size and the number of experimental replicates is missing from the figure legend. Fixed.

6. In Figure 3F, the absence of a pixel intensity scale bar makes the data difficult to interpret, as color values corresponding to high and low signal intensities are unclear. Moreover, no additional centrosome marker is included, nor is there evidence that PReM fragment expression levels are comparable across samples. These concerns also apply to Figures 4C and 4D. We now include pixel intensity scales in all relevant Figures. We think we do not need to show additional centrosome markers in our images as centrosomes exhibit a very reproducible behaviour in these embryos so we can be very confident that the objects we show here are genuine centrosomes. Considering expression levels, the images in Fig.4C,D (now 3C,D) are derived from stable transgenic lines so we can measure protein expression levels and show that the 2A and 2E mutants are expressed at similar levels to WT (new Figure S6). The images in 2F are from mRNA injections, so cannot be quantified in this way. However, we have vast experience with this assay (used in >15 publications since 2014) and can tell when, very occasionally, an injected mRNA is not expressed well (as this leads to a lack of general fluorescence in the cytoplasm). In addition, we know that deletions in Cnn do not generally destabilise the protein as we have analysed many such transgenic lines (see, for example, Reviewer Figure 1). Thus, the differences in centrosomal levels observed and quantified in 2F are almost certainly not caused by differences in the stability of the proteins being generated from the injected mRNAs.

Reviewer Figure 1

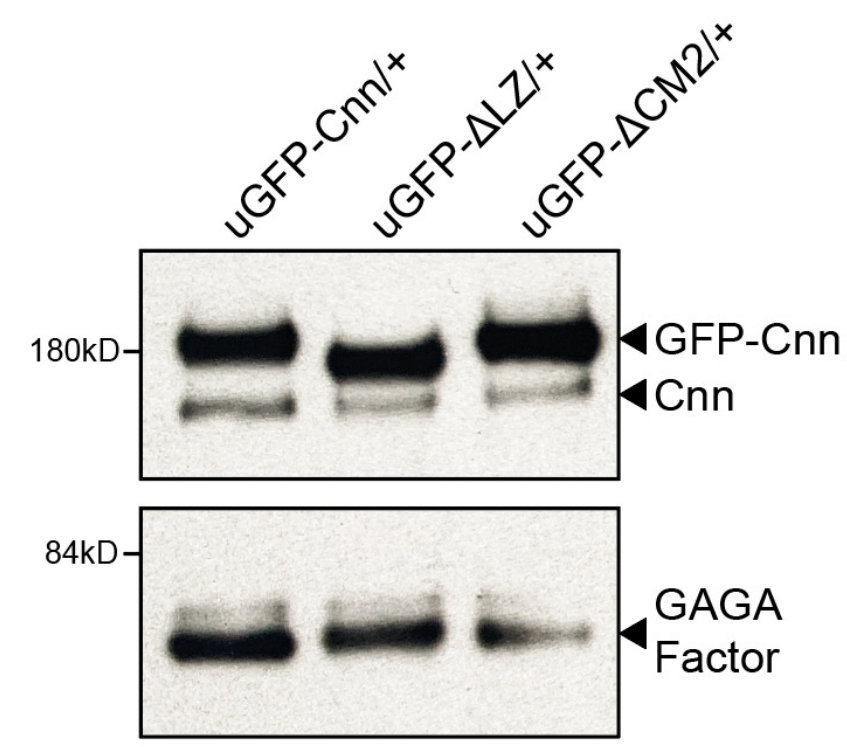

7. In Figure 4A, the interacting residues of PReM and CM2 shown in the red inset would be clearer if residue annotations for each domain were displayed in distinct colors. Additionally, the legends for Figures 4C and 4D do not specify the scale bar length. Fixed.

8. The authors state that interactions between CM2 and PReM-2A462-608 could not be detected in vitro based on SEC chromatograms (Figure 5A), yet the figure does not clearly show this result. The accompanying SDS-PAGE images are too small and lack lane labels, making interpretation difficult (a similar issue applies to Figure 7B). Furthermore, the SEC chromatogram x-axis lacks volume annotations, hindering correlation between chromatographic peaks and SDS-PAGE results (in contrast to Figure 7B, which provides an appropriate example). We thank the reviewer for these points, all of which have now been fixed/adjusted.

We hope the Reviewers will agree that these changes have improved the manuscript and that it would now be suitable for publication in *The EMBO Journal*.

Sincerely,

Dr. Jordan W Raff
University of Oxford
Sir William Dunn School of Pathology
South Parks Road
Oxford OX1 3RE
United Kingdom

12th May 2026

Re: EMBOJ-2026-124117-T
Phosphorylation relieves autoinhibition to drive Cnn centrosome scaffold assembly

Dear Jordan,

Thank you for submitting your revised Review Commons manuscript for consideration by The EMBO Journal. Given the interest of the subject and the constructive nature of the transferred referee reports, I decided to treat the work like a regular revision, and returned it directly to the original referees. Since they all consider the study significantly improved and most initially raised concerns addressed, we would be happy to pursue it further for EMBO Journal publication. Referees 1 and 2 nevertheless still have several issues that I would ask you to incorporate during a final round of minor revision. In addition, I would ask you to also take care of the following editorial points and adjustments to EMBO Journal format:
GENERAL:

- Please download and complete our author checklist (link provided below).

- Since we mandate addition of ORCID identifiers for all (co-)corresponding authors, please remind Dr. Hyman to add an ORCID to his author profile in our submission system. This needs to be added by Dr. Hyman personally and can unfortunately not be done by others on his behalf.

- Please provide suggestions for a short 'blurb' text prefacing and summing up the conceptual aspect of the study in two sentences (max. 250 characters), followed by 3-5 one-sentence 'bullet points' with brief factual statements of key results of the paper; they will form the basis of an editor-written 'Synopsis' accompanying the online version of the article. Please also upload a synopsis image, which can be used as a "visual title" for the synopsis section of your paper. The image should be in PNG or JPG format, and please make sure that it remains in the modest dimensions of (exactly) 550 pixels wide and 300 pixels high.

- Please make sure to include any relevant funding information both in the text (Acknowledgement section) and in our submission system.

- Please consider making the title somewhat more explicit and widely accessible (with our broad readership in mind), e.g. "Phosphorylation relieves centrosomin autoinhibition to promote its centrosomal scaffold assembly", or "Plk1 phosphorylation relieves centrosomin autoinhibition to promote its centrosomal scaffold assembly"?

- You shall also receive a separate message from our Source Data curation team, with instructions on how to prepare and upload relevant image and numerical raw data.

TEXT:

- Please upload the manuscript text as an editable DOCX file, without figures included, but legends amended at the end.

- Please adjust the order as well as the headers of the different manuscript sections: Title page with complete author information, Abstract, Keywords, Introduction, Results, Discussion, Methods, Data Availability, Acknowledgements, Disclosure and Competing Interests Statement, References, Main Figure Legends, Tables, Expanded Figure Legends.

- Please double-check to make sure that each figure panel (of main and expanded view figures) is called out in the text at least once and in sequential order (if referring to a multi-panel figure as a whole, consider simply referencing e.g. "Fig. 7A-D" first)

- Please note that Materials and Methods need to be described in the main text using our 'Structured Methods' format (for detail, see https://www.embopress.org/page/journal/14693178/authorguide#structuredmethods). The in-text "Methods" section should contain method and protocol descriptions (ideally using a step-by-step protocol format to facilitate adoption of the methodologies across labs), while all key reagents, experimental models, software and relevant equipment - including their sources and relevant identifiers - should be listed in a separately uploaded Reagents and Tools Table, a template for which can be downloaded from the above section of our Author Guidelines.

- Please consider moving some of the material in the included tables into the Reagents and Tool table, and to convert others into separately-uploaded Expanded View tables (callout: Table EV1/2/3...). Furthermore, since the present 'Table S1' is not in table format, please convert it into an additional main or expanded view figure.

- Please include a Disclosure and competing interests statement (next to the Acknowledgment section) - for details, see https://www.embopress.org/competing-interests

- Please carefully go through the reference list and make sure that each reference is complete with citation year, journal name, volume/page/elocator numbers - some of these informations are currently missing in several entries. Also, please adjust the format for citation of preprints as specified in our author guidelines:
The citation in the text should be: "(preprint: NAME1 et al, YEAR)"
The citation in the reference list: "NAME1, NAME2, ... (YEAR) ARTICLE TITLE. bioRxiv/medRxiv/ResearchSquare(...) doi: XXX"

- Please make sure to have deposited all structural data in the relevant external repositories at the time of resubmission, and make sure to include the respective accession codes as well as database URLs in the "Data Availability" section at the end of the Material and Methods.

DATA:

- Please upload all main Figures as individual, image-only files with sufficient resolution/quality for production.

- Please refer to our author guide (https://link.springer.com/journal/44318/submission-guidelines#cms-Expanded-View-data) regarding "supplementary information". The current "Figures S1-7" should best be converted into Expanded View Figures, and renamed Figure EV1-7, both in the legends and in the in-text callouts.

- Please rename the supplementary movie as Expanded View movie (in-text callouts "Movie EV1"). Its legends should be moved into an individual text, and then combined with the respective movie file into a separate ZIP file and uploaded as such.

- Finally, during routine pre-acceptance checks, our data editors have raised the following queries regarding figures, data, and legends; I would appreciate if you briefly answered to them in the cover letter of your final submission, and made the requested text modifications with changes/additions highlighted via the "Track changes" option, to facilitate our final checking:
1. Please define the annotated p values ****/***/**/* as well as provide the exact p-values for the same in the legend of figures 8E, F as appropriate.
2. Please note that the exact p values should be provided in the legends of figures 2E, F; 4B, 5B, C; 7C
3. Please indicate the statistical test used for data analysis in the legends of figures 8E, F
4. Please note that information related to n is missing in the legends of figures 8D, E, F
5. Please note that the error bars are not defined in the legends of figures 8D, F

Should you need additional guidance/feedback regarding this final adjustments, please do not hesitate to contact us directly. Thank you again for the opportunity to consider this work for The EMBO Journal, and I look forward to receiving your final version!

With kind regards,

Hartmut

Hartmut Vodermaier, PhD
Senior Editor, The EMBO Journal
h.vodermaier@embojournal.org

*** PLEASE NOTE: All revised manuscript are subject to initial checks for completeness and adherence to our formatting guidelines. Revisions may be returned to the authors and delayed in their editorial re-evaluation if they fail to comply to the following requirements. As a first step please read our guidelines for revised submissions:
https://link.springer.com/journal/44318/submission-guidelines#cms-Revised-submissions

1) Every manuscript requires a Data Availability section (even if only stating that no deposited datasets are included). Primary datasets or computer code produced in the current study have to be deposited in appropriate public repositories prior to resubmission, and reviewer access details provided in case that public access is not yet allowed.

2) Each figure legend must specify

- size of the scale bars that are mandatory for all micrograph panels
- the statistical test used to generate error bars and P-values
- the type error bars (e.g., S.E.M., S.D.)
- the number (n) and nature (biological or technical replicate) of independent experiments underlying each data point
- Figures may not include error bars for experiments with n<3; scatter plots showing individual data points should be used instead.

3) Revised manuscript text (including main tables, and figure legends for main and EV figures) has to be submitted as editable text file (e.g., .docx format). We encourage highlighting of changes (e.g., via text color) for the referees' reference.

4) Each main and each Expanded View (EV) figure should be uploaded as individual production-quality files (preferably in .eps, .tif, .jpg formats). For suggestions on figure preparation/layout, please refer to our Figure Preparation Guidelines:
https://media.springernature.com/original/springer-cms/rest/v1/content/27825798/data/v1

5) Point-by-point response letters should include the original referee comments in full together with your detailed responses to them (and to specific editor requests if applicable), and also be uploaded as editable (e.g., .docx) text files.

6) Please complete our Author Checklist, and make sure that information entered into the checklist is also reflected in the manuscript; the checklist will be available to readers as part of the Review Process File.

7) All authors listed as (co-)corresponding need to deposit, in their respective author profiles in our submission system, a unique ORCiD identifier linked to their name. Please see our Guide to Authors for detailed instructions.

8) Please note that supplementary information at EMBO Press has been superseded by the 'Expanded View' for inclusion of additional figures, tables, movies or datasets; with up to five EV Figures being typeset and directly accessible in the HTML version of the article.

9) To facilitate reproducibilty and cross-laboratory adoption of methodologies, please structure the Materials & Methods section as outlined in our guide to authors, including a completed Reagents and Tools Table.

10) Digital image enhancement is acceptable practice, as long as it accurately represents the original data and conforms to community standards. If a figure has been subjected to significant electronic manipulation, this must be clearly noted in the figure legend and/or the 'Materials and Methods' section. The editors reserve the right to request original versions of figures and the original images that were used to assemble the figure. Finally, we generally encourage uploading of numerical as well as gel/blot image source data.

At EMBO Press we ask authors to provide source data for the main manuscript figures. You will receive a separate email with instructions for providing source data with your revised manuscript, including how to upload and organize the files.

In the interest of ensuring the conceptual advance provided by the work, we recommend submitting a revision within 3 months (10th Aug 2026). Please discuss the revision progress ahead of this time with the editor if you require more time to complete the revisions. Use the link below to submit your revision:

Link Not Available

Referee #1:

Main Points:

1) From previous studies, it seems to me that for the residues potentially relevant for the hairpin regulation there is direct evidence of phosphorylation only for S567 (mass spec, phospho-antibody). Have the authors tested single site mutants (S567A and E)? Also, have they tested D mutations? If so, this should be commented on and shown. If not, it should be tested, in particular since the 2E phospho-mimetic is not functioning properly in vivo. If S571 is indeed crucial, it should be demonstrated that it is also phosphorylated. Otherwise it is possible that the mutation of this residue simply impairs important interactions (e.g. PReM-CM2, others), independent of phosphorylation.
As requested, we have now tested individual S567A and S571A mutations and found that they both perturb Cnn scaffold assembly, but to a lesser extent than the 2A double mutant (New Fig.S3A). We also now confirm by MS that recombinant Polo can phosphorylate both S567 and

S571 in vitro, and we have examined the behaviour of a 2D mutant and find that it behaves very similarly to the 2E mutant (New Fig.S3B).

>I don't find any mass spec data regarding in vitro kinase assays anywhere (see also point 3 below). I found a table S1 that seems to indicate phosphorylated residues for a subset of kinase assays by orange dot symbols, but there is no explanation how to interpret this table. In any case, the authors need to provide primary mass spec data in an excel format that led them to conclude which residues are specifically phosphorylated by incubation with recombinant kinase, as is standard for this type of experiment.

3) Regarding the worm PReM and CM2 domains, the authors mention that they have tested in vitro phosphorylation by PLK-1, but I could not find any data showing this. They should demonstrate successful phosphorylation or test candidate site by phospho-mimetic mutation. It is possible that the worm proteins depend more strongly on phosphorylation to relieve autoinhibition compared to the fly proteins.
This is a good point, and we apologise for this omission. We now state that we confirmed by MS analysis that the recombinant worm PLK-1 we used in these in vitro experiments phosphorylates the putative SPD-5 PReM domain on the three sites (S627, S653 and S658) known to be important for promoting SPD-5 scaffold assembly in vivo (Figure Legend, Figure 6). Thus, the lack of detectable binding between these proteins is not due to the lack of phosphorylation.

> As I said, this was already stated in the previous manuscript version, but corrresponding mass spec data were not included. The revised manuscript still lacks these data (see also comment in point 1 above).

Minor Point:
4). Fig. 6C, D: the labeling of the chimeric constructs using "+" symbols is confusing, since it suggests that separate proteins were expressed. If I understand this correctly, with the current labeling, deltaCM2+DmCM2 means WT? The authors should write the full name of the wildtype or chimeric construct in each case and use a more standard/less confusing nomenclature. Also, I suggest to start the panels and graphs with the WT sample.
We thank the Reviewer for this suggestion and have re-labelled this Figure to clarify this point. We understand the point about putting the WT panels first in Figure 6C,D (now Figure 5C,D) but think that this is not the correct comparison to emphasise. We are testing the ability of the various CM2 domains to "rescue" the lack of a CM2 domain, so we feel Drosophila Cnn lacking CM2 is the correct baseline for this comparison.

> The images in panel C still have the old labeling. Also, for consistency, I would use the same labeling in the graphs.

Referee #2:

In their study, Mohamad et al. providing compelling structural and cell biology data that reveals an elegant mechanism for PCM assembly in flies. Their data suggest that the scaffold protein Cnn exists in an autoinhinbited state, which is relieved by Polo phosphorylation on helices in the PReM domain. Upon phosphorylation, these helices are displaced, opening up an interaction motif that can dock with the C-terminal CM2 domain. They further show that this specific mechanism of PReM-CM2 interaction may be similar in humans but does not exist in C. elegans. Thus, they demonstrate unique differences in the mechanism of PCM scaffold assembly across species. However, to me, this is not the most exciting point. I feel that their data, combined with the work of the Oegema, Conduit, Woodruff and Qi labs, make a compelling argument that there is a unifying design principle underlying PCM scaffold assembly and activation across distant species: Polo Kinase mediated relief of autoinhibition, which effectively unfolds the scaffold and reveals key binding sites for other scaffold molecules and the microtubule nucleating complexes.
Overall, I think the study is well executed and the conclusions are mostly supported by the data. The authors have improved the writing, data presentation, and scope of the paper significantly since the initial submission. There are still a few corrections and considerations needed. Once these are addressed, I support publication of this paper in the EMBO Journal.

Specific Comments.

Figure 6A. The AF3 prediction for the SPD-5 dimer being anti-parallel is likely wrong. In Rios et al., 2024 the cross-linking data clearly support a parallel dimer configuration. This was demonstrated with Rosetta modeling (which uses the cross-links as distance constraints to build the model) and AF2 (selecting the best model using the cross-links). Please revise.

Figure 6B. Consider enlarging the images. It is hard to see the text.

Figure 8. The authors need to specifically mention in the main text and Figure that they used 4% PEG (MW: 20 kDa) to induce condensate formation. There is some debate about whether PEG is physiological or not. Furthermore, I'm not aware of anyone

using 20 kDa PEG before. Most labs have used PEG in the 3-8 kDa range. This information is important when comparing experiments across papers, especially since depletion attraction forces are affected by the molecular mass of the crowding agent.

Figure 8. ". . .could not purify sufficient quantities of soluble CDK5RAP2 for our studies, perhaps because we did not retain an MBP-tag (Rios et al, 2025)."

This statement is incorrect. Rios et al. (2025) used MBP-Selector columns (MBP nanobody linked to beads) to purify MBP-3C-CDK5RAP2, then eluted using 3C protease. Thus, the protein used in their assays did not include MBP.

The likely reason that the authors could not get sufficient CDK5RAP2 protein is because they used Amylose beads (MBP-Trap is the same thing) and tried to elute using maltose. Amylose beads are notorious for sticking non-specifically to large coiled-coil proteins.

Please revise the statement. Just say "we could not purify sufficient quantities of soluble CDK5RAP2". Anyway, I don't think it is necessary for the authors to study purified CDK5RAP2. I think it's enough to simply mention that others have seen CDK5RAP2 can form condensates.

Figure 8. This figure is missing information about sample size and what the error bars indicate.

Figure 8. "Thus, although the PReM and CM2 domains are not strictly required for condensate formation in vitro, they appear to modulate the behaviour of the molecules within the Cnn condensates, but not the SPD-5 condensates."

Mutating the CM2 and PReM domains have a significant effect on SPD-5 condensate size, which is similar to the Cnn condensates. One possibility is that these domains affect the rate of "aging" or "hardening", by which a condensate naturally solidifies over time. Interactions mediated by CM2 and PReM could lock the scaffold proteins in place, which is known to halt condensate growth; this concept was proposed by Ranganathan and Shakhnovich (2020) and demonstrated for PLK-1-driven SPD-5 hardening in Amato et al., 2025. Differences in the rate of condensate aging might also explain some of the differences they see between full-length Cnn and SPD-5. It is possible that Cnn ages faster, which would explain why Cnn condensates are smaller, less spherical, and less dynamic than SPD-5 condensates.

Abstract and Discussion. The authors seem to focus on the differences between assembly mechanisms of SPD-5 and Cnn, owing to the differences in needing the CM2-PReM interaction. However, my takeaway from their results is that there is a powerful unifying principle at work: PCM assembly is driven through Polo Kinase-mediated opening of scaffold proteins. Thus, despite the differences in domain architecture and amino acid sequence, there exists a common engineering principle. To me, that should be the final point made in the Abstract and Discussion.

The authors touch on this point in the discussion by mentioning the mechanism of gamma TuRC binding. They should also discussion the XL-MS study from Rios et al. (2024) which showed how extensively SPD-5 restructures into an open configuration after phosphorylation (see data for SDS-Monomers in Figure 1).

I leave it to the discretion of the authors whether they want to change the abstract or not.

Referee #3:

The authors has addressed our concerns, and I have no further comments.

***

Rev_Com_number: RC-2025-03287
New_manu_number: EMBOJ-2026-124117-T
Corr_author: Raff
Title: Phosphorylation relieves autoinhibition to drive Cnn centrosome scaffold assembly

SIR WILLIAM DUNN SCHOOL OF PATHOLOGY

University of Oxford, South Parks Road, Oxford OX1 3RE

Professor Jordan Raff
César Milstein Chair of Cancer Biology
Tel: (+44) 01865 275533 (direct)
jordan.raff@path.ox.ac.uk

Melissa Wright
PA to Professor Raff
Tel: (+44) 01865 27559
Fax: (+44) 01865 275515
melissa.wright@path.ox.ac.uk

8th July 2026

Dear Hartmut,

Thank you for sending us the Reviewer's comments on our revised manuscript, and apologies that it has taken us so long to properly comply with your formatting and data-deposition instructions. We believe that we are now fully compliant with your instructions and, perhaps most importantly, all raw data is now available and has been released with links in the Data Availability section. We believe we did provide a link in our original resubmission to a Dropbox folder with the raw data, but perhaps this didn't work for some reason? As you indicated the paper was unlikely to go back to the Reviewers, we have now deposited everything in the appropriate repository and provided links in the Data Availability section (and ticked the "opt out" option for each Figure).

We are of course delighted that you have decided to consider the manuscript further for publication at *EMBO Journal*, and I address the Reviewer's remaining concerns below.

Reviewer #1

The Reviewer requested the primary MS data in an Excel format. We apologise for this omission as our MS Facility suggested that it was now common practice that they permanently hold the raw data and provide a link to this in the final version of the accepted paper. We now provide accessible summary Figures (Fig EV4 and EV7), Excel spreadsheets with the primary data for each construct analysed (deposited in Zenodo), and a link to the raw MS data (held at the ProteomeXchange Consortium); all links provided in the Data Availability section.

The Reviewer also noted some labelling issues in Fig 5C that we have corrected.

Reviewer #2

Specific comments:

Figure 6A. The AF3 prediction for the SPD-5 dimer being anti-parallel is likely wrong. In Rios et al., 2024 the cross-linking data clearly support a parallel dimer configuration. This was demonstrated with Rosetta modeling (which uses the cross-links as distance constraints to build the model) and AF2 (selecting the best model using the cross-links). Please revise.

Throughout the paper we use AlphaFold3 (AF3) for our structural predictions and the top 5 predictions are all closely related versions of this anti-parallel conformation. In contrast, as the reviewer describes, Rios et al., used AF2ColabFold and Rosetta and selected the model that best fitted their cross-linking data. Intriguingly, when we revisited this, we found that even without this guidance AF2 selects a parallel conformation while AF3 selects an antiparallel conformation. We do not understand the basis for this discrepancy, but we now mention this

in the Figure legend, and explain why we continue to show the AF3 anti-parallel prediction to maintain methodological consistency throughout our paper.

Figure 6B. Consider enlarging the images. It is hard to see the text.

Done.

Figure 8. The authors need to specifically mention in the main text and Figure that they used 4% PEG (MW: 20 kDa) to induce condensate formation. There is some debate about whether PEG is physiological or not. Furthermore, I'm not aware of anyone using 20 kDa PEG before. Most labs have used PEG in the 3-8 kDa range. This information is important when comparing experiments across papers, especially since depletion attraction forces are affected by the molecular mass of the crowding agent.

We now highlight the use of 4% PEG (MW: 20kDa) in the main text and explain our rationale for using it in the Materials and Methods (p45, para.,2).

Figure 8. ". . .could not purify sufficient quantities of soluble CDK5RAP2 for our studies, perhaps because we did not retain an MBP-tag (Rios et al, 2025)."

This statement is incorrect. Rios et al. (2025) used MBP-Selector columns (MBP nanobody linked to beads) to purify MBP-3C-CDK5RAP2, then eluted using 3C protease. Thus, the protein used in their assays did not include MBP.

The likely reason that the authors could not get sufficient CDK5RAP2 protein is because they used Amylose beads (MBP-Trap is the same thing) and tried to elute using maltose. Amylose beads are notorious for sticking non-specifically to large coiled-coil proteins.

Please revise the statement. Just say "we could not purify sufficient quantities of soluble CDK5RAP2". Anyway, I don't think it is necessary for the authors to study purified CDK5RAP2. I think it's enough to simply mention that others have seen CDK5RAP2 can form condensates.

We apologise for quoting the Rios et al., paper incorrectly. We have now rewritten this section along the lines suggested (p15, para.3).

Figure 8. This figure is missing information about sample size and what the error bars indicate.

We apologise for this omission and now supply this data in the Figure legend.

Figure 8. "Thus, although the PReM and CM2 domains are not strictly required for condensate formation in vitro, they appear to modulate the behaviour of the molecules within the Cnn condensates, but not the SPD-5 condensates."

Mutating the CM2 and PReM domains have a significant effect on SPD-5 condensate size, which is similar to the Cnn condensates. One possibility is that these domains affect the rate of "aging" or "hardening", by which a condensate naturally solidifies over time. Interactions mediated by CM2 and PReM could lock the scaffold proteins in place, which is known to halt condensate growth; this concept was proposed by Ranganathan and Shakhnovich (2020) and demonstrated for PLK-1-driven SPD-5 hardening in Amato et al., 2025. Differences in the rate of condensate aging might also explain some of the differences they see between

full-length Cnn and SPD-5. It is possible that Cnn ages faster, which would explain why Cnn condensates are smaller, less spherical, and less dynamic than SPD-5 condensates.

These are all good points, and we now clarify throughout the manuscript that the deletion of CM2 and PReM differentially effects the *dynamics* of the SPD-5 and Cnn condensates. The Reviewer's explanations for several aspects of these condensate's behaviours are very plausible, but without further experiments are speculative. We think this level of speculation is probably not warranted by the data—and would slightly distract from our main conclusions—so we have not commented on these points further.

Abstract and Discussion. The authors seem to focus on the differences between assembly mechanisms of SPD-5 and Cnn, owing to the differences in needing the CM2-PReM interaction. However, my takeaway from their results is that there is a powerful unifying principle at work: PCM assembly is driven through Polo Kinase-mediated opening of scaffold proteins. Thus, despite the differences in domain architecture and amino acid sequence, there exists a common engineering principle. To me, that should be the final point made in the Abstract and Discussion.

The authors touch on this point in the discussion by mentioning the mechanism of gamma TuRC binding. They should also discussion the XL-MS study from Rios et al. (2024) which showed how extensively SPD-5 restructures into an open configuration after phosphorylation (see data for SDS-Monomers in Figure 1).

I leave it to the discretion of the authors whether they want to change the abstract or not.

These are all good points, and we have now rejigged several parts of the manuscript (including the Abstract) to focus on this aspect more clearly.

We hope you will agree that these changes have improved the manuscript and that it will now be acceptable for publication at EMBO Journal.

With best wishes,

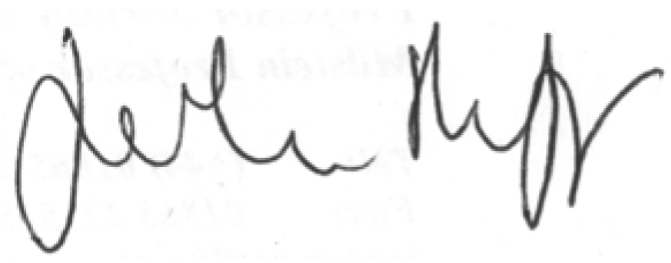

EMBO Press Author Checklist

Corresponding Author Name: Jordan Raff	
Journal Submitted to: The EMBO Journal	
Manuscript Number: EMBOJ-2026-124117R	

USEFUL LINKS FOR COMPLETING THIS FORM
The EMBO Journal - Author Guidelines
EMBO Reports - Author Guidelines
Molecular Systems Biology - Author Guidelines
EMBO Molecular Medicine - Author Guidelines

Reporting Checklist for Life Science Articles (updated January
This checklist is adapted from Materials Design Analysis Reporting (MDAR) Checklist for Authors. MDAR establishes a minimum set of requirements in transparent reporting in the life sciences (see Statement of Task: 10.31222/osf.io/9sm4x). Please follow the journal's guidelines in preparing your
Please note that a copy of this checklist will be published alongside your article.

Abridged guidelines for figures
1. Data
The data shown in figures should satisfy the following conditions:
- the data were obtained and processed according to the field's best practice and are presented to reflect the results of the experiments in an accurate and unbiased manner.
- ideally, figure panels should include only measurements that are directly comparable to each other and obtained with the same assay.
- plots include clearly labeled error bars for independent experiments and sample sizes. Unless justified, error bars should not be shown for technical
- if n<5, the individual data points from each experiment should be plotted. Any statistical test employed should be justified.
- Source Data should be included to report the data underlying figures according to the guidelines set out in the authorship guidelines on Data

2. Captions
Each figure caption should contain the following information, for each panel where they are relevant:
- a specification of the experimental system investigated (eg cell line, species name).
- the assay(s) and method(s) used to carry out the reported observations and measurements.
- an explicit mention of the biological and chemical entity(ies) that are being measured.
- an explicit mention of the biological and chemical entity(ies) that are altered/varied/perturbed in a controlled manner.
- the exact sample size (n) for each experimental group/condition, given as a number, not a range;
- a description of the sample collection allowing the reader to understand whether the samples represent technical or biological replicates (including how many animals, litters, cultures, etc.).
- a statement of how many times the experiment shown was independently replicated in the laboratory.
- definitions of statistical methods and measures:
  - common tests, such as t-test (please specify whether paired vs. unpaired), simple χ2 tests, Wilcoxon and Mann-Whitney tests, can be unambiguously identified by name only, but more complex techniques should be described in the methods section;
  - are tests one-sided or two-sided?
  - are there adjustments for multiple comparisons?
  - exact statistical test results, e.g., P values = x but not P values < x;
  - definition of 'center values' as median or average;
  - definition of error bars as s.d. or s.e.m.

Please complete ALL of the questions below.
Select "Not Applicable" only when the requested information is not relevant for your study.

Materials

Newly Created Materials	Information included in the manuscript?	In which section is the information available? (Reagents and Tools Table, Materials and Methods, Figures, Data Availability Section)
New materials and reagents need to be available; do any restrictions apply?	Yes	Materials and Methods

Antibodies	Information included in the manuscript?	In which section is the information available? (Reagents and Tools Table, Materials and Methods, Figures, Data Availability Section)
For **antibodies** provide the following information:<br>- Commercial antibodies: RRID (if possible) or supplier name, catalogue number and or/clone number<br>- Non-commercial: RRID or citation	Yes	Materials and Methods

DNA and RNA sequences	Information included in the manuscript?	In which section is the information available? (Reagents and Tools Table, Materials and Methods, Figures, Data Availability Section)
**Short novel DNA or RNA including primers, probes**: provide the sequences.	Yes	Materials and Methods

Cell materials	Information included in the manuscript?	In which section is the information available? (Reagents and Tools Table, Materials and Methods, Figures, Data Availability Section)
**Cell lines:** Provide species information, strain. Provide accession number in repository **OR** supplier name, catalog number, clone number, and/**OR** RRID.	Not Applicable	
**Primary cultures:** Provide species, strain, sex of origin, genetic modification status.	Not Applicable	
Report if the cell lines were recently **authenticated** (e.g., by STR profiling) and tested for mycoplasma contamination.	Not Applicable	

Experimental animals	Information included in the manuscript?	In which section is the information available? (Reagents and Tools Table, Materials and Methods, Figures, Data Availability Section)
**Laboratory animals or Model organisms:** Provide species, strain, sex, age, genetic modification status. Provide accession number in repository **OR** supplier name, catalog number, clone number, **OR** RRID.	Yes	
**Animal observed in or captured from the field:** Provide species, sex, and age where possible.	Not Applicable	
Please detail **housing and husbandry conditions**.	Yes	Materials and Methods

Plants and microbes	Information included in the manuscript?	In which section is the information available? (Reagents and Tools Table, Materials and Methods, Figures, Data Availability Section)
**Plants:** provide species and strain, ecotype and cultivar where relevant, unique accession number if available, and source (including location for collected wild specimens).	Not Applicable	
**Microbes:** provide species and strain, unique accession number if available, and source.	Not Applicable	

Human research participants	Information included in the manuscript?	In which section is the information available? (Reagents and Tools Table, Materials and Methods, Figures, Data Availability Section)
If collected and within the bounds of privacy constraints report on age, sex and gender or ethnicity for all study participants.	Not Applicable	

Core facilities	Information included in the manuscript?	In which section is the information available? (Reagents and Tools Table, Materials and Methods, Figures, Data Availability Section)
If your work benefited from core facilities, was their service mentioned in the acknowledgments section?	Yes	Acknowledgements

Design

Study protocol	Information included in the manuscript?	In which section is the information available? (Reagents and Tools Table, Materials and Methods, Figures, Data Availability Section)
If study protocol has been **pre-registered, provide DOI in the manuscript**. For clinical trials, provide the trial registration number **OR** cite DOI.	Not Applicable	
Report the **clinical trial registration number** (at ClinicalTrials.gov or equivalent), where applicable.	Not Applicable	

Laboratory protocol	Information included in the manuscript?	In which section is the information available? (Reagents and Tools Table, Materials and Methods, Figures, Data Availability Section)
Provide DOI OR other citation details if **external detailed step-by-step protocols** are available.	Not Applicable	

Experimental study design and statistics	Information included in the manuscript?	In which section is the information available? (Reagents and Tools Table, Materials and Methods, Figures, Data Availability Section)
Include a statement about **sample size** estimate even if no statistical methods were used.	Yes	Figures
Were any steps taken to minimize the effects of subjective bias when allocating animals/samples to treatment (e.g. **randomization procedure**)? If yes, have they been described?	Yes	Figures
Include a statement about **blinding** even if no blinding was done.	Yes	Figures
Describe **inclusion/exclusion criteria** if samples or animals were excluded from the analysis. Were the criteria pre-established?<br><br>If sample or data points were omitted from analysis, report if this was due to attrition or intentional exclusion and provide justification.	Not Applicable	
For every figure, are **statistical tests** justified as appropriate? Do the data meet the assumptions of the tests (e.g., normal distribution)? Describe any methods used to assess it. Is there an estimate of variation within each group of data? **I**s the variance similar between the groups that are being statistically compared?	Yes	Figures

Sample definition and in-laboratory replication	Information included in the manuscript?	In which section is the information available? (Reagents and Tools Table, Materials and Methods, Figures, Data Availability Section)
In the figure legends: state number of times the experiment was **replicated** in laboratory.	Yes	Figures
In the figure legends: define whether data describe **technical or biological replicates**.	Yes	Figures

Ethics

Ethics	Information included in the manuscript?	In which section is the information available? (Reagents and Tools Table, Materials and Methods, Figures, Data Availability Section)
Studies involving **human participants**: State details of **authority granting ethics approval** (IRB or equivalent committee(s), provide reference number for approval.	Not Applicable	
Studies involving **human participants**: Include a statement confirming that **informed consent** was obtained from all subjects and that the experiments conformed to the principles set out in the WMA Declaration of Helsinki and the Department of Health and Human Services Belmont Report.	Not Applicable	
Studies involving **human participants:** For publication of **patient photos**, include a statement confirming that consent to publish was obtained.	Not Applicable	
Studies involving experimental **animals**: State details of **authority granting ethics approval** (IRB or equivalent committee(s), provide reference number for approval. Include a statement of compliance with ethical regulations.	Not Applicable	
Studies involving **specimen and field samples:** State if relevant **permits** obtained, provide details of authority approving study; if none were required, explain why.	Not Applicable	

Dual Use Research of Concern (DURC)	Information included in the manuscript?	In which section is the information available? (Reagents and Tools Table, Materials and Methods, Figures, Data Availability Section)
Could your study fall under dual use research restrictions? Please check biosecurity documents and list of **select agents and toxins** (CDC): https://www.selectagents.gov/sat/list.htm	Not Applicable	
If you used a select agent, is the security level of the lab appropriate and reported in the manuscript?	Not Applicable	
If a study is subject to dual use research of concern regulations, is the name of the **authority granting approval and reference number** for the regulatory approval provided in the manuscript?	Not Applicable	

Reporting

The MDAR framework recommends adoption of discipline-specific guidelines, established and endorsed through community initiatives. Journals have their own policy about requiring specific guidelines and recommendations to complement MDAR.

Adherence to community standards	Information included in the manuscript?	In which section is the information available? (Reagents and Tools Table, Materials and Methods, Figures, Data Availability Section)
State if relevant guidelines or checklists (e.g., **ICMJE, MIBBI, ARRIVE, PRISMA**) have been followed or provided.	Not Applicable	
For **tumor marker prognostic studies,** we recommend that you follow the **REMARK** reporting guidelines (see link list at top right). See author guidelines, under 'Reporting Guidelines'. Please confirm you have followed these guidelines.	Not Applicable	
For **phase II and III randomized controlled trials**, please refer to the **CONSORT** flow diagram (see link list at top right) and submit the CONSORT checklist (see link list at top right) with your submission. See author guidelines, under 'Reporting Guidelines'. Please confirm you have submitted this list.	Not Applicable	

Data Availability

Data availability	Information included in the manuscript?	In which section is the information available? (Reagents and Tools Table, Materials and Methods, Figures, Data Availability Section)
Have **primary datasets** been deposited according to the journal's guidelines (see 'Data Deposition' section) and the respective accession numbers provided in the Data Availability Section?	Yes	Materials and Methods
Were **human clinical and genomic datasets** deposited in a public access-controlled repository in accordance to ethical obligations to the patients and to the applicable consent agreement?	Not Applicable	
Are **computational models** that are central and integral to a study available without restrictions in a machine-readable form? Were the relevant accession numbers or links provided?	Not Applicable	
If publicly available data were reused, provide the respective **data citations in the reference list**.	Yes	Materials and Methods
