# Supplementary material for: Polo/PLK1 phosphorylation relieves Centrosomin/Cnn autoinhibition to promote centrosome scaffold assembly
Source: EMBO J. 2026 Jul 30;45(17):6320–56. doi: 10.1038/s44318-026-00878-x (PMC13534702; doi:10.1038/s44318-026-00878-x)
Supplement: Supplementary file 2 [file 44318_2026_878_MOESM7_ESM.pdf]

Expanded View Figures

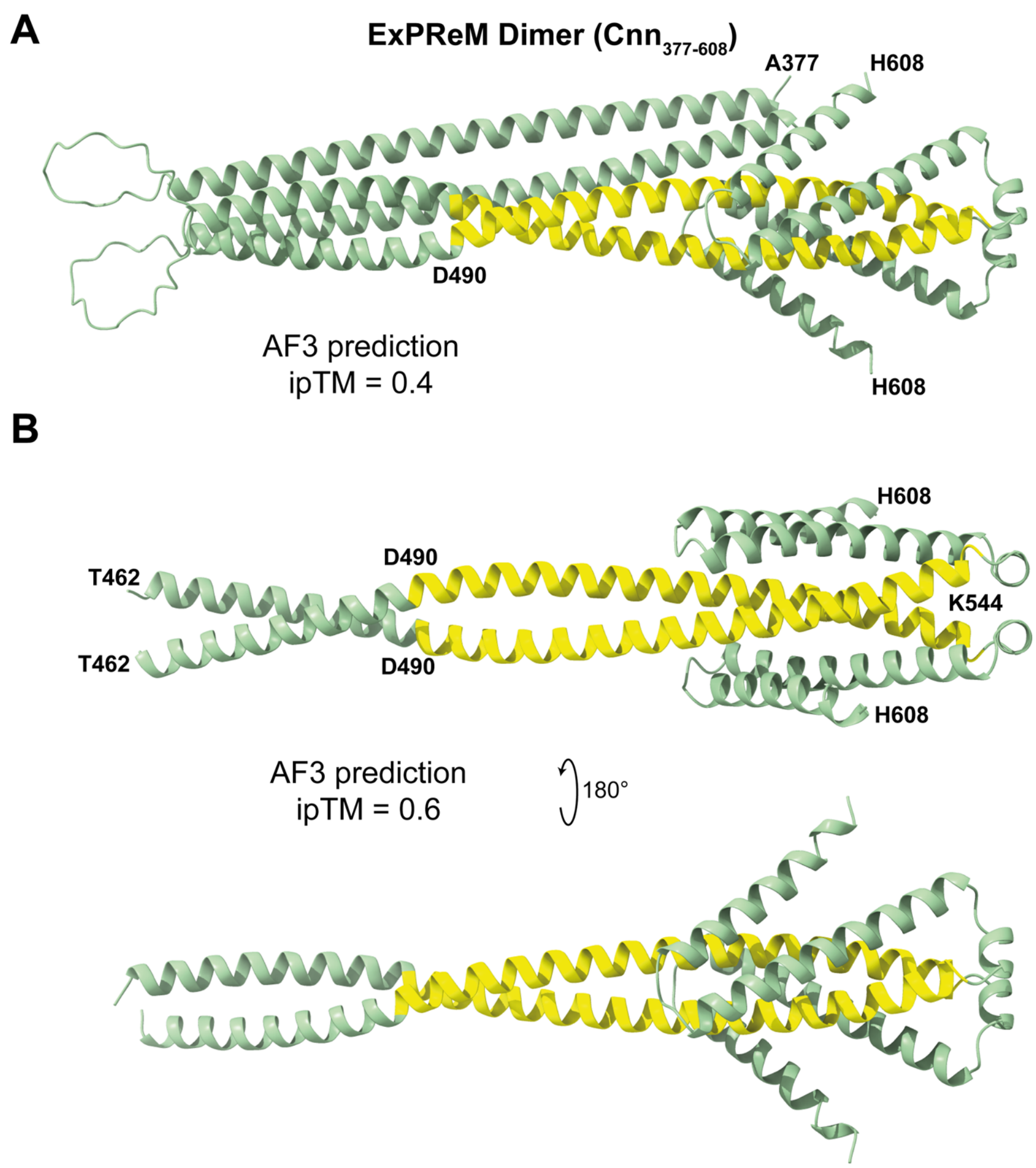

Figure EV1. Predicted structures of PReM domain dimers.

Ribbon representations of AF3 predictions of the Extended PReM (ExPReM) domain dimer ($Cnn_{377\text{-}608}$) (**A**), and what appears to be the "core" helical hairpin structure of the PReM domain ($Cnn_{462\text{-}608}$) (incorporating H2 + H3 + H4 from the ExPReM structure) (**B**). The LZ is highlighted in yellow, and additional helices in green. See also Movie EV1.

 © The Author(s)

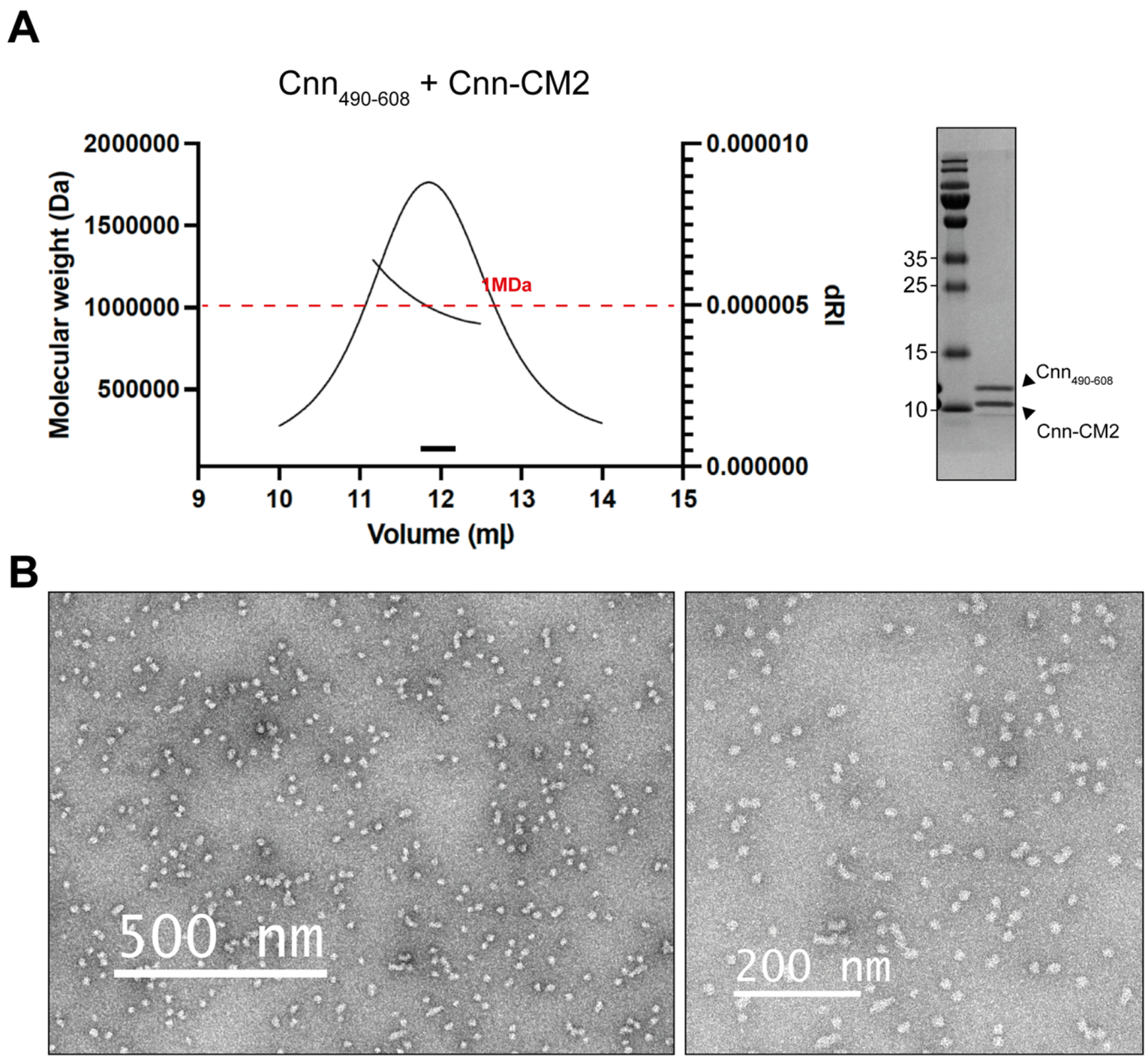

Figure EV2. Cnn$_{490-608}$ forms high MW (~1 MDa) complexes when mixed with CM2, rather than extended scaffolds.

(**A**) Traces of a SEC MALS experiment (the fraction highlighted by the black bar is shown on the associated SDS gel) showing that Cnn$_{490-608}$ forms high MW complexes of ~ 1 MDa when mixed with the CM2 domain in vitro. (**B**) Transmission EM images of the Cnn$_{490-608}$::CM2 complexes.

© The Author(s)

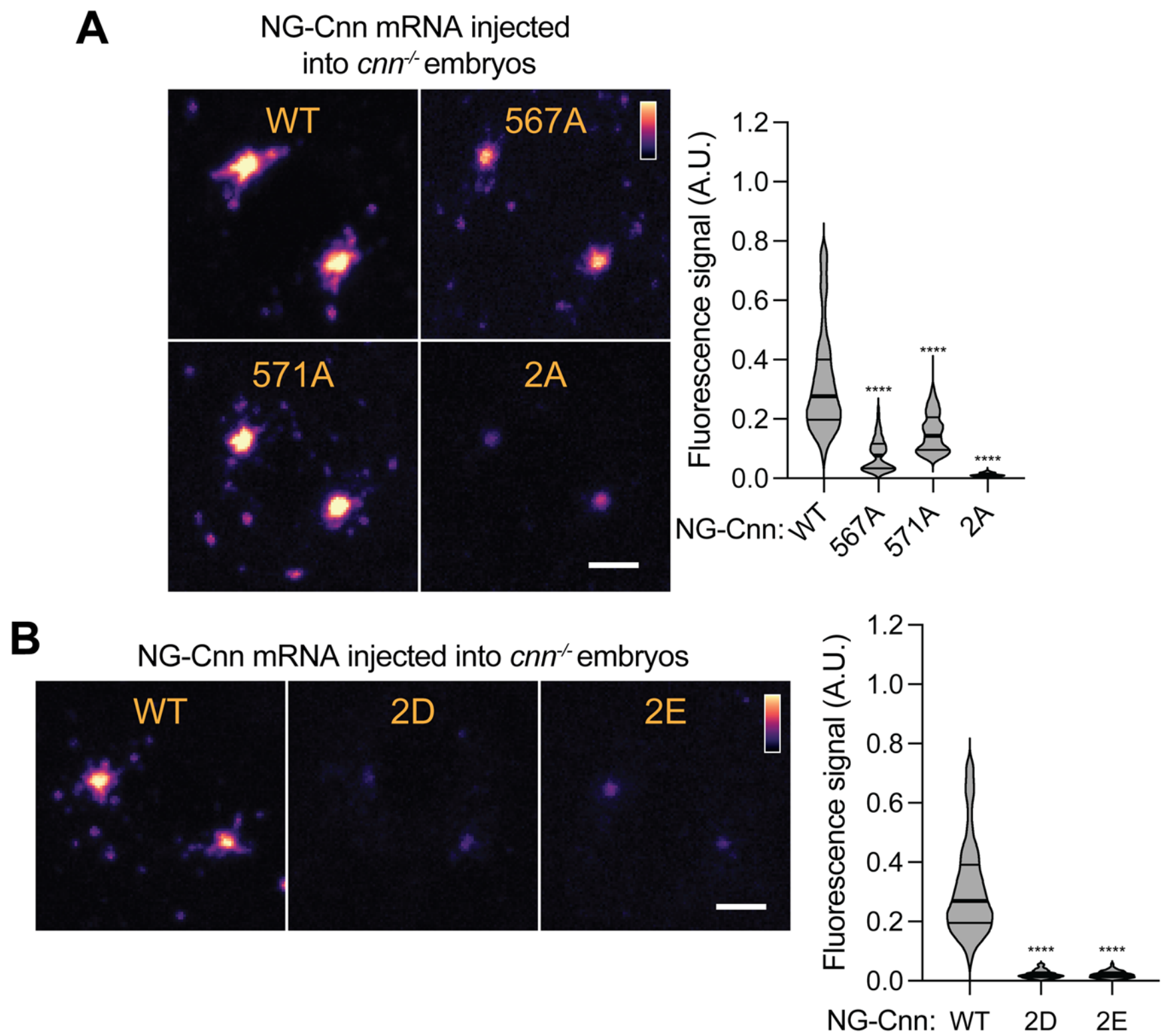

Figure EV3. Single S567A and S571A substitutions perturb Cnn scaffold assembly in vivo.

Images illustrate, and violin plots quantify, the in vivo centrosomal fluorescence intensity (mean±first and third quartile) of NG-fusions of WT, S567A, S571A or S567A,S571A (2A) Cnn proteins expressed from mRNAs injected into *cnn*$^{-/-}$ embryos (**A**) or WT, S567D,S571D (2D), or S567E,S571E (2E) proteins expressed from mRNAs injected into embryos laid by heterozygous *cnn* mutant females (*cnn*$^{+/-}$ embryos) (**B**). Note that image brightness was adjusted slightly differently for the 567A, 2A, 2D, and 2E constructs to allow visualisation of centrosomes, which were otherwise too dim to be clearly visualised. Statistical significance was assessed using a one-way ANOVA analysis with Dunnett's multiple comparison test in GraphPad Prism (****$P < 0.0001$). $N = > 10$ embryos, $n = >500$ centrosomes for each NG-fusion. Scale bar = 2 µm.

© The Author(s)

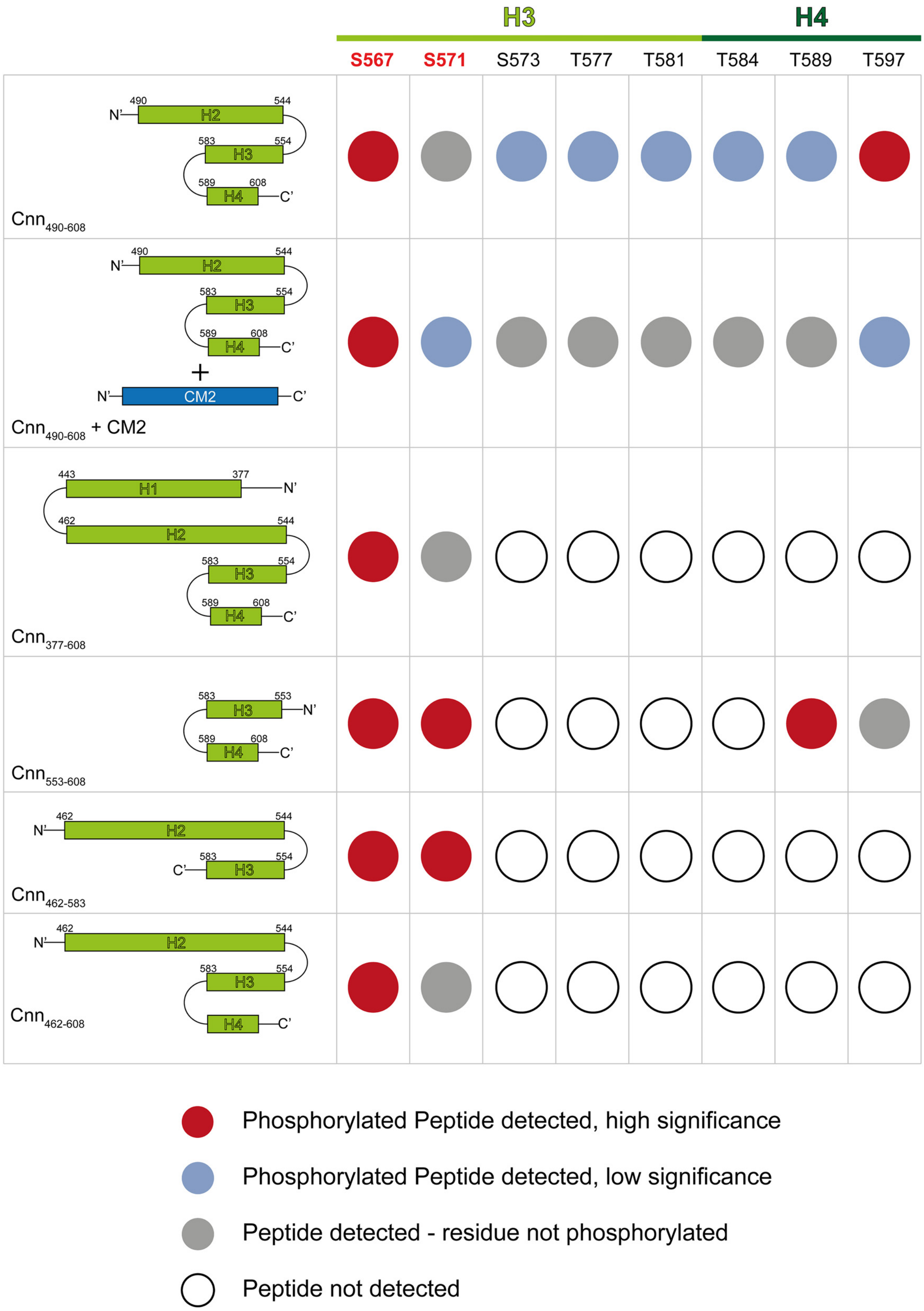

Figure EV4. Summary of Mass Spec (MS) analysis of in vitro Polo-dependent phosphorylation of PReM domain constructs.

Schematics on the left indicate the recombinant constructs that were mixed with purified *Drosophila* Polo kinase and then analysed by MS to identify phosphorylated residues. The position of the conserved Ser/Thr residues in the C-terminal region of the PReM (containing helix H3 and H4) are indicated above (S567 and S571, the most relevant for this study, are highlighted in red), and a coloured dot indicates the result of the MS analysis. Red dot indicates the phosphorylated peptide was identified with high significance; blue dot indicates the phosphorylated peptide was identified but with low significance; grey dot indicates that the peptide was identified with high significance, but it was not phosphorylated. No dot indicates that no peptide covering the residue was identified.

© The Author(s)

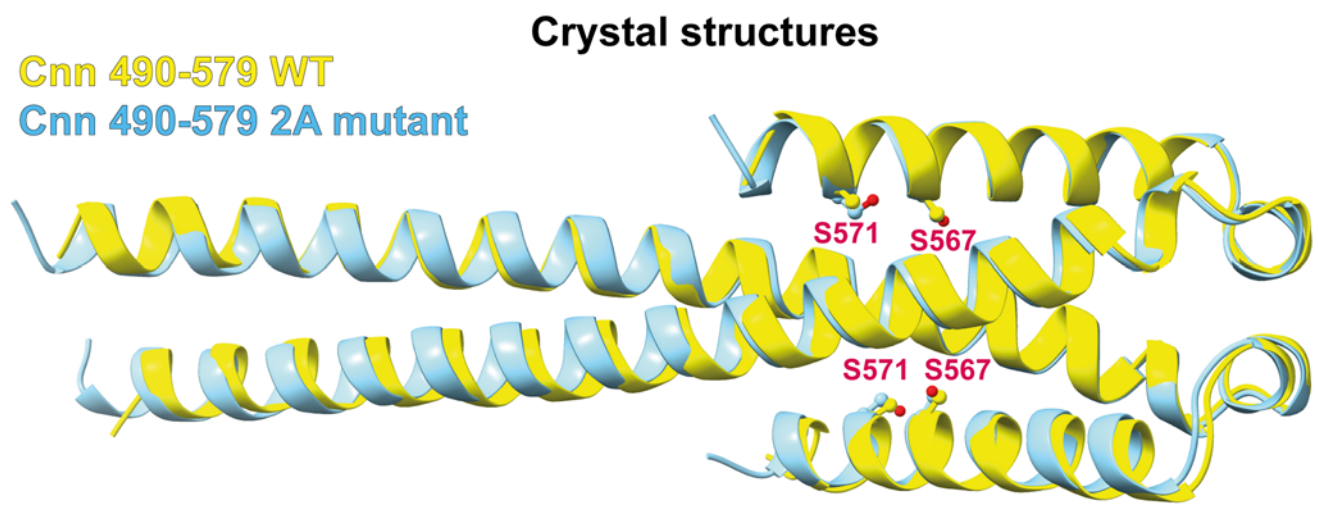

Figure EV5. Cnn-2A mutations do not perturb the helical hairpin fold of the LZ+ domain.

An overlay of the crystal structures of the WT (yellow) and 2A mutant (blue) $Cnn_{490\text{-}579}$ LZ+ domains reveals that the mutations do not significantly alter the helical hairpin structure.

 © The Author(s)

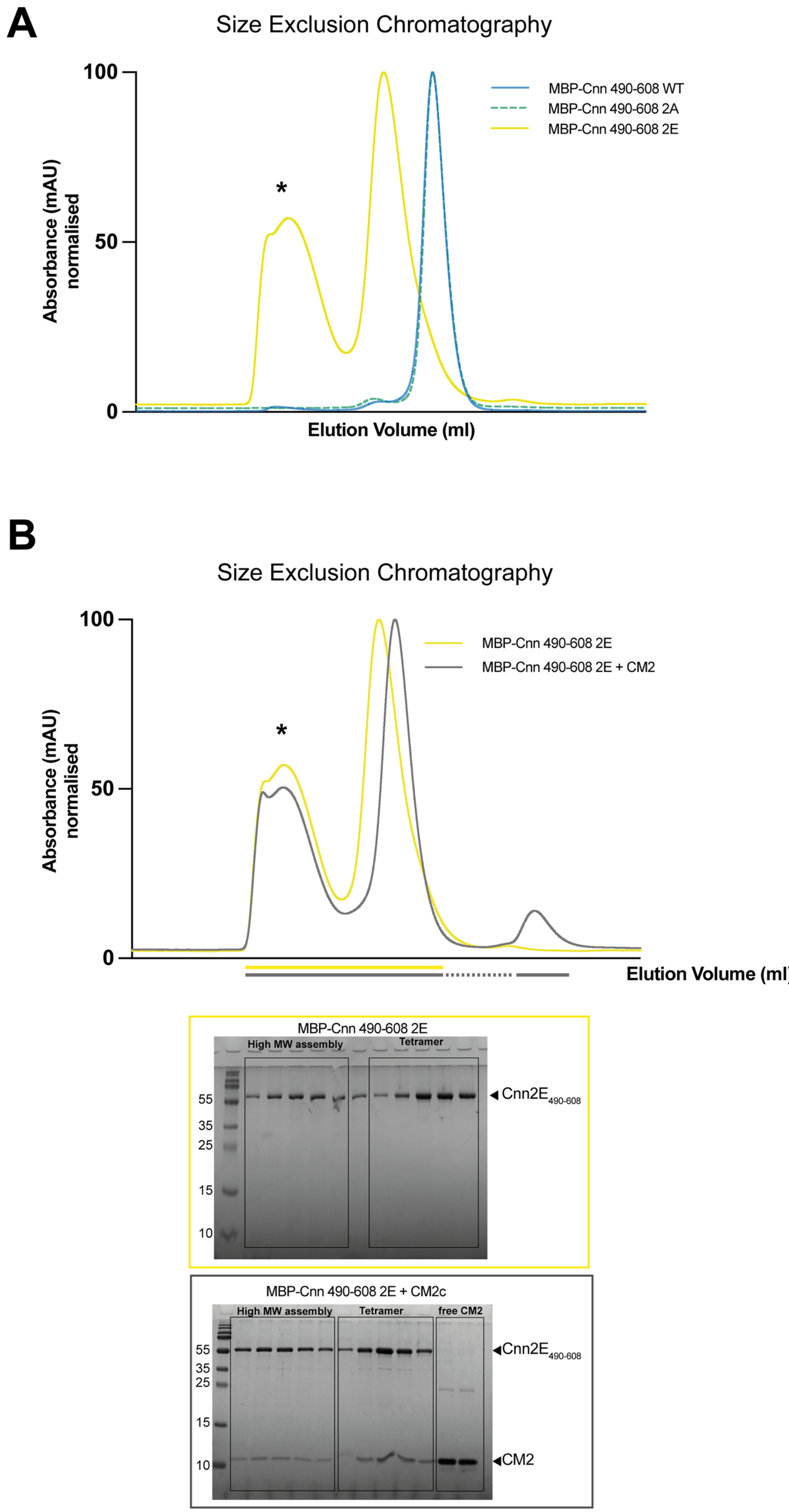
A
Size Exclusion Chromatography
100
50
0
Absorbance (mAU) normalised
Elution Volume (ml)
*
MBP-Cnn 490-608 WT
MBP-Cnn 490-608 2A
MBP-Cnn 490-608 2E
B
Size Exclusion Chromatography
100
50
0
Absorbance (mAU) normalised
Elution Volume (ml)
*
MBP-Cnn 490-608 2E
MBP-Cnn 490-608 2E + CM2
MBP-Cnn 490-608 2E
High MW assembly
Tetramer
55
35
25
15
10
Cnn2E490-608
MBP-Cnn 490-608 2E + CM2c
High MW assembly
Tetramer
free CM2
55
35
25
15
10
Cnn2E490-608
CM2

Figure EV6. Size-exclusion chromatography analysis of MBP-Cnn$_{490–608}$ 2A and 2E variants and their interaction with CM2.

(**A**) Graph shows overlay of size-exclusion chromatography (SEC) profiles of WT (blue), 2A (dotted green) and 2E (yellow) variants of MBP-Cnn$_{490-608}$. As shown previously, the WT protein elutes as a single well-behaved dimer species (Feng et al, 2017), and we found the 2A mutant behaved similarly. In contrast, the 2E mutant exhibits two main peaks: a high-molecular-weight peak that elutes close to the void volume (asterisk) and a second peak that runs close to the WT and 2A dimer peak, but is shifted slightly towards earlier elution volumes (indicating a higher molecular weight than a dimer—probably a tetramer). We conclude that the 2E substitutions allow MBP-Cnn$_{490-608}$ to interact with itself in ways that the WT and 2A proteins do not. (**B**) Graph shows overlay of SEC profiles of MBP–Cnn$_{490-608}$ 2E either alone (yellow)—same data as shown in (**A**)—or when incubated with CM2 (black). Insets below show SDS-gels of selected fractions from the elution profiles (as indicated by the solid lines underneath each SEC trace). The addition of CM2 alters the elution profile of MBP–Cnn$_{490-608}$ 2E slightly. The void peak largely overlaps with the peak of the 2E protein alone, although the SDS-gel indicates that the CM2 protein is also incorporated into these higher molecular species. The "tetramer" peak also contains CM2 but is shifted slightly towards later elution volumes. This indicates a lower molecular weight than the postulated 2E tetramer (probably a 2E::CM2 tetramer). These data show that, in contrast to the 2A mutations, the 2E mutations do not prevent binding to CM2, and they are consistent with the possibility that the 2E mutations help to "open" the hairpin to promote self-interactions. For ease of comparison, absorbance at 280 nm is shown normalised in all graphs.

 © The Author(s)

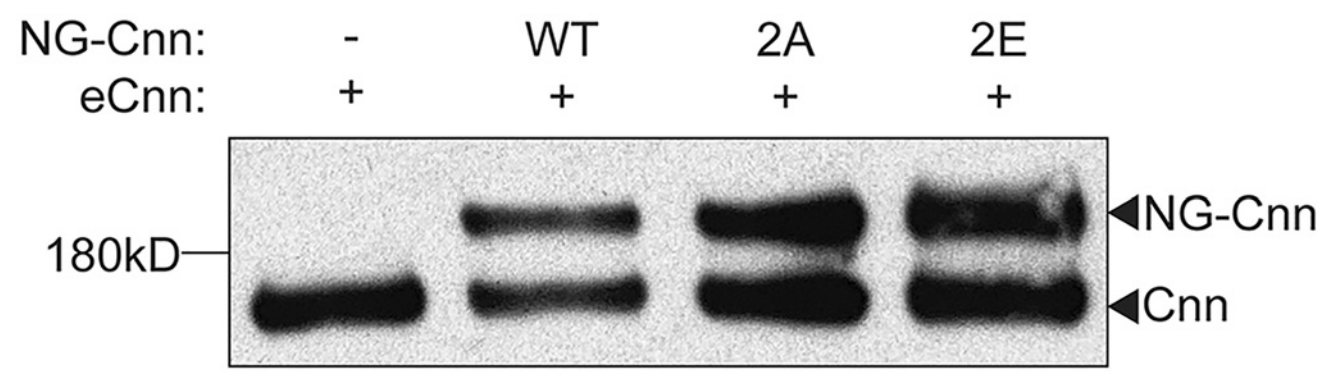

Figure EV7. Cnn2A and Cnn2E are expressed at similar levels to WT Cnn.

Western blot compares the expression levels of endogenous Cnn and the WT, 2A and 2E NG-fusions of Cnn in 0–2 h embryos laid by females expressing one copy of endogenous Cnn and one copy of the NG-fusion transgene. All the NG-fusions are present in embryos at similar levels to the endogenous protein.

© The Author(s)

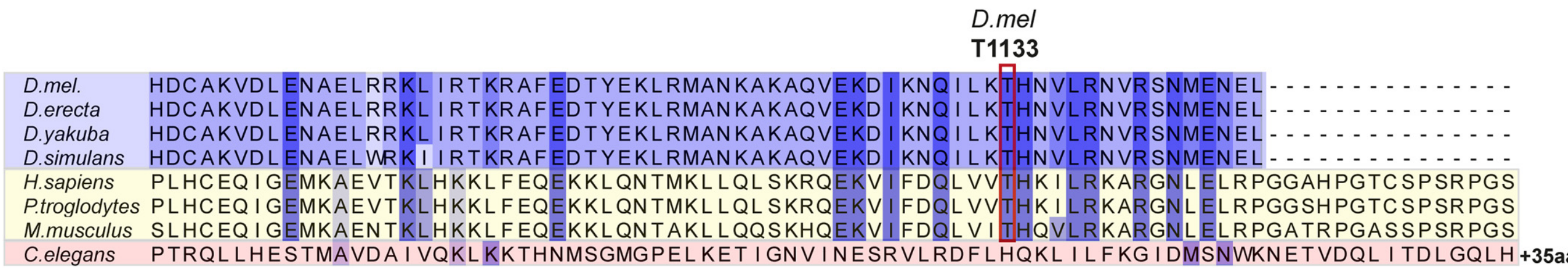

Figure EV8. The *Drosophila* CM2 domain shows sequence homology to the CM2 domain of human CDK5RAP2, but not to the putative CM2 domain of worm SPD-5.

A multiple sequence alignment of the CM2 (and putative CM2) domains of Cnn, CDK5RAP2 and SPD-5 from various species (*Drosophila* species boxed in blue, mammalian species in yellow and *C. elegans* in pink). The conserved Thr (T1133 in *Drosophila*) is highlighted (red box); in *Drosophila*, T1133 binds to the PReM in the same region that is occluded by S567 in the PReM helical hairpin structure (Fig. 4B), with S567 phosphorylation seeming to open the helical hairpin to allow CM2 to bind instead.

© The Author(s)

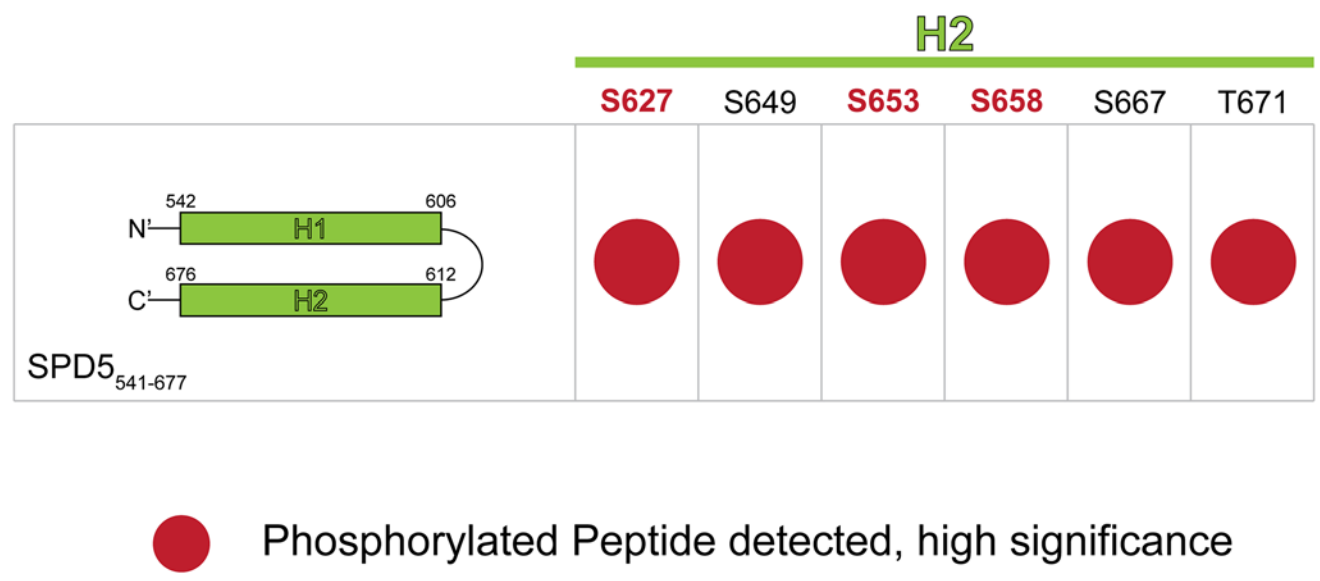

Figure EV9. Summary of a Mass Spec (MS) analysis of the in vitro PLK-1-dependent phosphorylation of a worm SPD-5 PReM domain.

The schematic on the left indicates the recombinant construct that was mixed with purified *C. elegans* PLK-1 kinase and then analysed by MS to identify phosphorylated residues. The position of Ser/Thr residues in the PReM is indicated above (S672, S635 and S658, whose phosphorylation has been shown to be important for SPD-5 scaffold assembly, are highlighted in red). The red coloured dot indicates peptides containing all of these phosphorylated Ser/Thr residues were identified with high significance.

© The Author(s)
