# Supplementary material for: Polo/PLK1 phosphorylation relieves Centrosomin/Cnn autoinhibition to promote centrosome scaffold assembly
Source: EMBO J. 2026 Jul 30;45(17):6320–56. doi: 10.1038/s44318-026-00878-x (PMC13534702; doi:10.1038/s44318-026-00878-x)
Supplement: Supplementary file 3 [file 44318_2026_878_MOESM1_ESM.docx]

**Table EV1***: Drosophila* stocks used in this study

| Allele | Source |
| --- | --- |
| cnn^f04547^ | Exelixis stock no. f04547, Exelixis Stock Centre (Harvard Medical School, Boston, MA). |
| cnn^HK21^ | (Megraw *et al*, 1999; Vaizel-Ohayon & Schejter, 1999) |
| Ubq-NG-Cnn | (Wong *et al*, 2022) |
| Ubq-NG-Cnn-2A | Generated in this study |
| Ubq-NG-Cnn-2E | Generated in this study |
| Jupiter-mCherry | Generated by the Daniel St Johnston lab |
