# Supplementary material for: Polo/PLK1 phosphorylation relieves Centrosomin/Cnn autoinhibition to promote centrosome scaffold assembly
Source: EMBO J. 2026 Jul 30;45(17):6320–56. doi: 10.1038/s44318-026-00878-x (PMC13534702; doi:10.1038/s44318-026-00878-x)
Supplement: Supplementary file 4 [file 44318_2026_878_MOESM2_ESM.docx]

**Table EV2**: *Drosophila* stocks used in specific experiments

| Genotype | Purpose | Fig |
| --- | --- | --- |
| cnn^f04547^ / cnn^HK21^ | For injection of various truncated Cnn mutants | 2F, 5C, S3 |
| cnn^f04547^, Ubq-NG-Cnn / cnn^HK21^ | Comparing the dynamics of Cnn phosphorylation null mutants | 3C |
| cnn^f04547^, Ubq-NG-Cnn-2A / cnn^HK21^ |  |  |
| Ubq-NG-Cnn / + | Comparing the dynamics of Cnn phosphorylation null and mimicking mutants | 3D |
| Ubq-NG-Cnn-2A / + |  |  |
| Ubq-NG-Cnn-2E / + |  |  |
| cnn^f04547^ / cnn^HK21^ ; Jupiter-mCherry / + | To determine whether heterologous CM2 domains could functionally substitute for fly CM2, we examined spindle morphology following injection of Cnn constructs in which the native CM2 was replaced with the *C. elegans* SPD-5 or human CDK5RAP2 CM2 domains. | 5D |
