# Supplementary material for: Polo/PLK1 phosphorylation relieves Centrosomin/Cnn autoinhibition to promote centrosome scaffold assembly
Source: EMBO J. 2026 Jul 30;45(17):6320–56. doi: 10.1038/s44318-026-00878-x (PMC13534702; doi:10.1038/s44318-026-00878-x)
Supplement: Supplementary file 5 [file 44318_2026_878_MOESM3_ESM.docx]

**Table EV3**: *Primers used in this study*

| Index | Primer | Purpose |
| --- | --- | --- |
| s1 | *5'-* GTT GTC ACT CAT GGA ACG GCC GCT GGA G | Amplifying WT-TACC cDNA and adding attB sites on both ends of the cDNA, which allowed the cDNA to be flipped into a pDonor plasmid |
| s2 | *5'-* CAC AAA ACT TTC AAT TCC CAC GAA GAG CTG CAC GC |  |
| s3 | *5’-* AAG CAG TTG CAG ACG GAA GTA AAG AAG AAG ACG GC |  |
| s4 | *5’-* TTC ACG GAG GCG GAG TAC ATG AGG GC |  |
| s5 | *5’-* GAT CAA CAG AAC AGC GCT GTG ATT GGG CAG TTG |  |
| s6 | *5’-* CAA CAC GCC AAG AAC ATC TTT GTG CTT CAG CAG AGA G |  |
| s7 | *5’-* TGT AGC TGT TAT ATT CAG AGT CAT ATT AAG ACT CTT GGA AAG ATC CAA GCT GCG ATC |  |
| s8 | *5’-* TAG GAC CCA GCT TTC TTG TAC AAA GTG GTT GAT GG | CM2 truncation |
| s9 | *5’-* GTC GTG TAC TGT GGC TGC ACC AGT TG | CM2 truncation |
| s10 | *5’-* GCA GCC ACA GTA CAC GAC CCC CTT CAC TGT GAG CAG ATT GGA G | CDK5RAP2 swap |
| s11 | *5’-* GTA CAA GAA AGCT GGG TCT CAG GAG CCT GGT CTG CTG G | CDK5RAP2 swap |
| s12 | *5’-* CTG GTG CAG CCA CAG TAC ACG ACA GCC CTA CTC GAC AAT TGC TCC ACG AAT C | SPD5 swap |
| s13 | *5’-* AGT GGT AAC CAG ATC CGC GGC CGC TCA CTT TTT GCG AAT TTC CTT CAC AGC TCC TCC C | SPD5 swap |
| s14 | *5’-* TTG GCA AGA TCC AAG GCG CGA TCC ACC GCC TTA CGC ATG GCA TTG CG | Cnn S567A and S571A mutagenesis |
| s15 | *5’-* GAG TCT TAA TAT GAC TCT GAA TAT AAC AGC TAC ATC CTT GGC TGA TCA AAG CC | Cnn S567A and S571A or S567E and S571E mutagenesis |
| s16 | *5’-* TTC TCA AGA TCC AAC TCG CGA TCC ACC GCC TTA CGC ATG GCA TTG CG | Cnn S567E and S571E mutagenesis |
| s17 | 5’ - GGA TCG CGC CTT GGA TCT TTC CAA GAG TCT TAA TAT GAC | Cnn S567A mutagenesis |
| s18 | 5’ - CAA GGC GCG ATC CAC CGC CTT ACG C |  |
| s19 | 5’ - CTT GGA TCT TGC CAA GAG TCT TAA TAT GAC TCT GAA TAT AAC AGC TAC ATC C | Cnn S571A mutagenesis |
| s20 | 5’ - CTT GGC AAG ATC CAA GCT GCG ATC CAC CG |  |
| s21 | 5’ - CTT GGA TCT TGA CAA GAG TCT TAA TAT GAC TCT GAA TAT AAC | Cnn S567D and S571D mutagenesis |
| s22 | *5’ - CAA GAT CCA AGT CGC GAT CCA CCG* |  |
| s23 | 5’- AGG AAA ACC TGT ACT TCC AGG GTG CCA GCT CCC AGG AAA TG | NG-pETM14-Dm_Cnn-377 F |
| s24 | 5’- AGG AAA ACC TGT ACT TCC AGG GTC AGT TGC AGA CGG AAG TA | NG-pETM14-Dm_Cnn-403 F |
| s25 | 5’- AGG AAA ACC TGT ACT TCC AGG GTA CGG AGG CGG AGT ACA TG | NG-pETM14-Dm_Cnn-462 F |
| s26 | 5’- AGG AAA ACC TGT ACT TCC AGG GTG ATC AAC AGA ACA GCG CT | NG-pETM14-Dm_Cnn-490 F |
| s27 | 5’- TCG GAT CCG GTA CCT CAT TAG TGG CTT GCA TCA CCT TC | NG-pETM14-Dm_Cnn-608 R |
| s28 | 5’- GAA GTT CTG TTC CAG GGG CCC GCC AGC TCC CAG GAA ATG | pETM44-Dm_Cnn-377 F |
| s29 | 5’- GAA GTT CTG TTC CAG GGG CCC CAG TTG CAG ACG GAA GTA | pETM44-Dm_Cnn-403 F |
| s30 | 5’- GAA GTT CTG TTC CAG GGG CCC ACG GAG GCG GAG TAC ATG | pETM44-Dm_Cnn-462_F |
| s31 | 5’- GAA GTT CTG TTC CAG GGG CCC GAT CAA CAG AAC AGC GCT | pETM44-Dm _Cnn-490 F |
| s32 | 5’- GAT CCG GTA CCT CAT TAG TCA TTC AGA GTC ATA TTA AGA C | pETM44-Dm _Cnn-579 R |
| s33 | 5’- GAT CCG GTA CCT CAT TAG TCG TGG CTT GCA  TCACCTTC | pETM44-Dm_Cnn-608_R |
| s34 | 5’- AGG AAA ACC TGT ACT TCC AGG GTG GCA GCA AAA CCA TCT TC | NG-pETM14-Hs_CDK5RAP2-535 F |
| s35 | 5’- TCG GAT CCG GTA CCT CAT TAT TCT TCA AGG CAA ATA CT | NG-pETM14-Hs_CDK5RAP2-639 R |
| s36 | 5’- GAA GTT CTG TTC CAG GGG CCC CTT CAC TGT GAG CAG ATT | pETM44-Hs_CDK5RAP2-1812 F |
| s37 | 5’- GAT CCG GTA CCT CAT TAG TCT CAG GAG CCT GGT CTG CTG G | pETM44-Hs_CDK5RAP2-1893 R |
| s38 | 5’- TTC CAG GGG CCC CTG GGA CCT ACT CGA CAA TTG CTC | pGEX-6P-1-Ce_SPD5-1061 F |
| s39 | 5’- ACC CGG GAA TTC CGG GGA TCA CTT TTT GCG AAT TTC | pGEX-6P-1-Ce_SPD5-1198 R |
| s40 | 5’- AGG AAA ACC TGT ACT TCC AGG GTT CAC AAA GTC ACA TTC CA | NG-pETM14-SPD5-541 F |
| s41 | 5’- TCGGATCCGGTACCTCATTATTCAGCATCG  GGTGCAAT | NG-pETM14-SPD5-677 R |
| s42 | 5’- GAA GTT CTG TTC CAG GGG CCC CAC GAC TGT GCC AAG GTA | pETM44-Dm_Cnn-1082 F |
| s43 | 5’- GAT CCG GTA CCT CAT TAG TCT TAT AAC TCA TTC TCC AT | pETM44-Dm_Cnn-1148 R |
| s44 | 5’-ATA TCT GAA TGC GGC CGC AAT GGA CCA GTC TAA ACA GGT TTT G | pOEM1-Cnn-WT-mGFP |
| s45 | 5’- CGC TCA AAC ATG GAG AAT GAG TTA GGC GCG CCG ATC AAA TGA |  |
| s46 | 5’-ATA TCT GAA TGC GGC CGC AAT GGA CCA GTC TAA ACA GGT TTT G | pOEM1-Cnn-delPReM-mGFP |
| s47 | 5’- CGT GCG ATG CTT AAG ACT GTA GAG GGC GCG CCA TCA AAT GA |  |
| s48 | 5’- AGTAAGGAGATATCTGAGCTAACCAAGAAGAC  3’- CTGGATCCCGGCGCGCCGCTCGTGTAATGAG  GTTGAGCACAC | pOEM1-Spd-5delCM2L-mGFP:to replace the c-termini of SPD5-WT |
| s49 | 5’- GTG TGC TCA ACC TCA TTA CAC GAG CGG CGC GCC GGG ATC CAG |  |
| s50 | 5’-GATCAACTGGTTGTGgagCACAAGATTCTGCGT | pETM44- Hs_CDK5RAP2 T1863E mutagenesis F |
| s51 | 5’-ACGCAGAATCTTGTGctcCACAACCAGTTGATC | pETM44- Hs_CDK5RAP2 T1863E mutagenesis R |
