# Supplementary material for: Polo/PLK1 phosphorylation relieves Centrosomin/Cnn autoinhibition to promote centrosome scaffold assembly
Source: EMBO J. 2026 Jul 30;45(17):6320–56. doi: 10.1038/s44318-026-00878-x (PMC13534702; doi:10.1038/s44318-026-00878-x)
Supplement: Supplementary file 6 [file 44318_2026_878_MOESM4_ESM.docx]

**Table EV4**: *Plasmids used in this study*

| Index | Plasmid | Purpose | Source |
| --- | --- | --- | --- |
| p1 | pRNA-NG-Cnn | Plasmid contains non-phosphorylatable Cnn for RNA synthesis | Generated in this study |
| p2 | pRNA-NG-CnnΔ376-609 |  | Generated in this study |
| p3 | pRNA-NG-CnnΔ376-402 |  | Generated in this study |
| p4 | pRNA-NG-CnnΔ376-462 |  | Generated in this study |
| p5 | pRNA-NG-CnnΔ376-490 |  | Generated in this study |
| p6 | pRNA-NG-CnnΔ551-609 |  | Generated in this study |
| p7 | pRNA-NG-CnnΔ583-609 |  | Generated in this study |
| p8 | pRNA-NG-CnnΔCM2 |  | Generated in this study |
| p9 | pRNA-NG-Cnn-[CDK5RAP2::CM2] |  | Generated in this study |
| p10 | pRNA-NG-Cnn-[SPD5::CM2] |  | Generated in this study |
| p11 | pUbq-NG-Cnn-2A | Plasmid encodes NeonGreen-tagged Cnn (S567A and S571A), under the control of a polyubiquitin promoter, for the generation of transgenic fly lines through *P*-element mediated transformation | Generated in this study |
| p12 | pUbq-NG-Cnn-2E | Plasmid encodes NeonGreen-tagged Cnn (S567E and S571E), under the control of a polyubiquitin promoter, for the generation of transgenic fly lines through *P*-element mediated transformation | Generated in this study |
| p13 | pRNA-NG-Cnn-2A |  |  |
| p14 | pRNA-NG-Cnn-2E |  |  |
| p15 | pRNA-NG-Cnn-S567A |  |  |
| p16 | pRNA-NG-Cnn-S571A |  |  |
| p17 | pRNA-NG-Cnn-2D |  |  |
| p18 | NG-pETM14-Dm_Cnn 377-608 |  | Generated in this study |
| p19 | NG-pETM14-Dm_Cnn 403-608 |  | Generated in this study |
| p20 | NG-pETM14-Dm_Cnn 462-608 |  | Generated in this study |
| p21 | NG-pETM14-Dm_Cnn 490-608 |  | Generated in this study |
| p22 | pETM44-Dm_Cnn 377-608 |  | Generated in this study |
| p23 | pETM44-Dm_Cnn 462-608 |  | Generated in this study |
| p24 | pETM44-Dm_Cnn 490-608 |  | Generated in this study |
| p25 | pETM44-Dm _Cnn 490-579 |  | Generated in this study |
| p26 | pETM44-Dm _Cnn 490-579-2A | Plasmid encodes MBP -tagged Cnn 490-579 (S567A and S571A mutants) | Generated in this study |
| p27 | NG-pETM14-Hs_CDK5RAP2- PReM 535-639 |  | Generated in this study |
| p28 | pETM44-Hs_CDK5RAP2-CM2 1812-1893 |  | Generated in this study |
| p29 | pETM44- Hs_CDK5RAP2 T1863E CM2 1812-1893 |  | Generated in this study |
| P30 | pGEX-6P-1-Ce_SPD5- CM2 1061-1198 |  | Generated in this study |
| p31 | NG-pETM14-SPD5-PReM 541-677 |  | Generated in this study |
| p32 | pETM44-Dm_Cnn-CM2 1082-1148 |  | Generated in this study |
| p33 | pOEM1-MBP-long-linker_Cnn-WT-mGFP-long-linker-His10 (TH2036-pOCC502) |  | Generated in this study |
| p34 | pOEM1-MBP-long-linker_Cnn-delPReM-mGFP-long-linker-His10 (TH2253-pOCC502) | Fragment-Cnn1-delPReM (403-608)-Bsu36I-PspOMI synthesised by Twist Bioscience in 2022 to generate plasmid encoding Cnn-delPReM construct | Generated in this study |
| p35 | pOEM1-MBP-linker_Cnn-delCM2-mGFP-linker-His6 (TH1592-pOCC120) |  | Generated in this study |
| p36 | pOEM1-MBP-linker_SPD-5-WT-mGFP-linker-His6 (TH0860-pOCC27) |  | (Woodruff *et al*, 2015) |
| p37 | pOEM1-MBP-linker_SPD-5-delPReM (541-677)-mGFP-linker-His6 (TH2475-pOCC27) | Fragment PmlI-AflII (1. Spd5_del541-677_artSpeI) with corresponding deletion and artificial SpeI (by silent mutagenesis) was synthesised by GenScript Biotech in 2024 to generate the SPD-5delPReM deletion construct. | Generated in this study |
| p38 | pOEM1-MBP-linker_SPD-5-delCM2L(1161-1198)-mGFP-linker-His6 (TH2077-pOCC27) |  | Generated in this study |
| p39 | pOEM1-worm_PLK-1-mGFP-linker-His6 (TH1091-pOCC8) |  | (Woodruff *et al*, 2015) |
| P40 | pOEM1-MBP-long-linker_Dm_polo-T182D(CA)-mGFP-long-linker-His10 (TH2194-pOCC502) |  | Generated in this study |
| p41 | pOEM1-MBP--linker_Dm_polo-WT-mcherry-linker-His6 (TH1547-pOCC216) |  | Generated in this study |
| p42 | pOEM1-MBP--linker_Dm_polo-T182D(CA)-untagged-linker-His6 (TH1553-pOCC-127) |  | Generated in this study |
